# Structure–Activity Relationships of 8-Hydroxyquinoline-Derived Mannich Bases with Tertiary Amines Targeting Multidrug-Resistant Cancer

**DOI:** 10.1021/acs.jmedchem.2c00076

**Published:** 2022-05-25

**Authors:** Veronika F. S. Pape, Roberta Palkó, Szilárd Tóth, Miklós J. Szabó, Judit Sessler, György Dormán, Éva A. Enyedy, Tibor Soós, István Szatmári, Gergely Szakács

**Affiliations:** † Institute of Enzymology, Research Centre for Natural Sciences, Eötvös Loránd Research Network, Magyar Tudósok körútja 2, H-1117 Budapest, Hungary; ‡ Department of Physiology, Semmelweis University, Faculty of Medicine, Tűzoltó utca 37-47, H-1094 Budapest, Hungary; § Institute of Organic Chemistry, Research Centre for Natural Sciences, Eötvös Loránd Research Network, Magyar Tudósok körútja 2, H-1117 Budapest, Hungary; ∥ ChemAxon Ltd., Váci út 133, H-1138 Budapest, Hungary; ⊥ TargetEx Ltd., Madách Imre u 31/2., H-2120 Dunakeszi, Hungary; # Department of Inorganic and Analytical Chemistry, MTA-SZTE Lendület Functional Metal Complexes Research Group, University of Szeged, Dóm tér 7, H-6720 Szeged, Hungary; ∇ Institute of Pharmaceutical Chemistry and Stereochemistry Research Group of Hungarian Academy of Sciences, University of Szeged, Eötvös u. 6, H-6720 Szeged, Hungary; ○ Institute of Cancer Research, Medical University of Vienna, Borschkegasse 8a, A-1090 Vienna, Austria

## Abstract

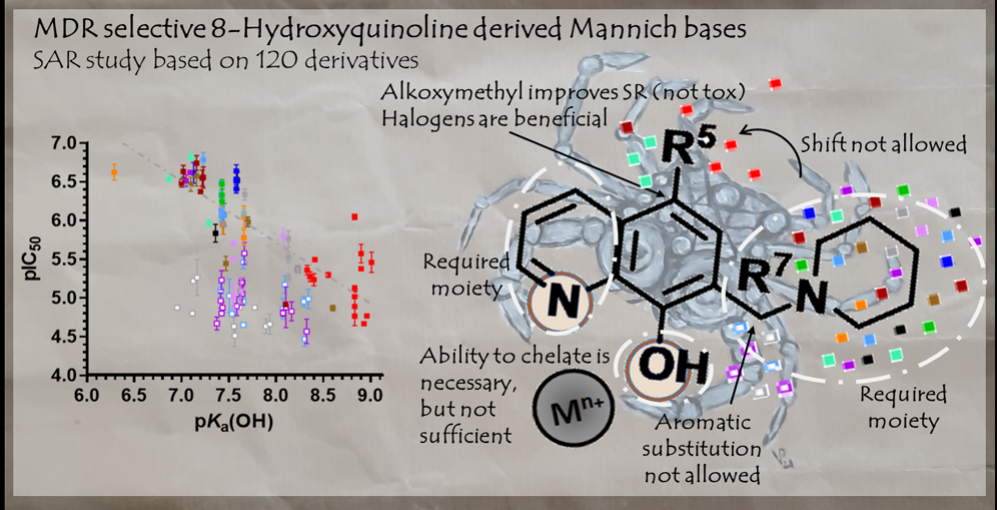

A recently proposed strategy to overcome multidrug resistance (MDR) in cancer is to target the collateral sensitivity of otherwise resistant cells. We designed a library of 120 compounds to explore the chemical space around previously identified 8-hydroxyquinoline-derived Mannich bases with robust MDR-selective toxicity. We included compounds to study the effect of halogen and alkoxymethyl substitutions in R5 in combination with different Mannich bases in R7, a shift of the Mannich base from R7 to R5, as well as the introduction of an aromatic moiety. Cytotoxicity tests performed on a panel of parental and MDR cells highlight a strong influence of experimentally determined p*K*_a_ values of the donor atom moieties, indicating that protonation and metal chelation are important factors modulating the MDR-selective anticancer activity of the studied compounds. Our results identify structural requirements increasing MDR-selective anticancer activity, providing guidelines for the development of more effective anticancer chelators targeting MDR cancer.

## Introduction

The Mannich reaction is a powerful tool in medicinal chemistry, contributing to the synthesis of novel chemical entities or the optimization of the physicochemical properties of drug candidates.^1,2^ Variations of the Mannich reaction are used in the synthesis of anticancer agents, antibacterial and antifungal compounds, antimalarials, and antiviral candidates.^1,2^ A potential substrate of the Mannich reaction is 8-hydroxyquinoline (8-OHQ), which is a privileged structure in many biologically active compounds and several marketed drugs^3−6^ used for the treatment of infectious diseases (5-nitro-8-OHQ), neuropathies (5-chloro-7-iodo-8-OHQ, clioquinol), and cancers. Therapeutic strategies using 8-OHQs target key enzymes such as the iron-containing ribonucleotide reductase involved in DNA synthesis^7,8^ or matrix metalloproteinases involved in metastasis.^9^ Further metalloenzyme targets include cytosolic and nuclear oxygenases,^10^ histone demethylases,^11^ and the HIF prolylhydroxylase.^12^ In addition, metal complexes formed with 8-OHQ ligands possess intrinsic anticancer activity by modulating cellular metal- and redox homeostasis.^4,13−15^ Extensive data from the literature suggest that the diverse biological activities of 8-OHQ derivatives can be fine-tuned by modification of the substitution pattern of the scaffold. Aromatic amide substitution at position 2 on the quinoline ring (R2) was shown to increase lipophilicity and antiviral activity by the electron-withdrawing properties of the anilide substituents.^16^ Introduction of glucoconjugates has been suggested as a prodrug-development strategy^17^ and even resulted in the increased anticancer activity of 8-OHQs against some cancer cell lines.^18^ Substitution at position 5 on the quinoline ring (R5) with electron-withdrawing substituents improved anticancer activity,^19^ while substitution with sulfonic acid (sulfoxine, 8-OH-5-quinolinesulfonic acid) decreased cytotoxicity, probably due to hindered cell permeability.^20^ Mannich bases with R7 substitution of 5-Cl-8-OHQ showed higher activity against matrix metalloproteinases 2 and 9, as compared to derivatives with aminomethyl substitution at R5.^9^

Recently, we have discovered a group of 8-hydroxyquinoline-derived Mannich bases possessing a unique anticancer activity against multidrug-resistant cells.^15,21,22^ A frequent reason for the failure of cancer chemotherapy is the development of therapy resistance,^23,24^ which often extends to structurally and mechanistically unrelated drugs.^25^ While multidrug resistance (MDR) is a multifactorial process,^26^ a common mechanism is linked to the reduced cellular accumulation of xenobiotics mediated by energy-dependent efflux pumps belonging to the family of ATP-binding cassette (ABC) transporters.^25−32^ Of the MDR transporters conferring *in vitro* resistance to cytotoxic and targeted chemotherapy, the contribution of *P*-glycoprotein (*P*-gp, ABCB1) to treatment failure has been widely demonstrated in clinical studies.^33^ Despite promising *in vitro* results, successful clinical translation of MDR transporter inhibition remains elusive.^34−39^ However, *P*-gp is still considered an important target for drug development. An alternative drug development strategy is to exploit the collateral sensitivity of MDR cells by compounds whose toxicity is paradoxically increased by *P*-gp.^40−42^ Based on a pharmacogenomic approach correlating the anticancer profiles measured in the NCI-60 cell panel by the Developmental Therapeutics Program (DTP) of the National Cancer Institute,^43,44^ we identified MDR-selective compounds with robust *P*-gp-dependent toxicity across diverse cell lines.^21,45,46^ Whereas MDR-selective compounds identified by the pharmacogenomic approach are relatively diverse, an enrichment of metal chelators such as isatin-β-thiosemicarbazones^46^ and 8-hydroxyquinoline-derived Mannich bases was observed, suggesting that complex formation with endogenous metal ions could be key to the cytotoxicity of at least a subset of the MDR-selective compounds.^15,21,22,42,46^ In particular, the abundance of the 8-hydroxyquinoline scaffold is striking, as represented by the 7-diethylaminomethyl derivative NSC693872 (**1**), the 7-pyrrolidin-1-yl-methyl derivative NSC693871 (**2**),^46^ and the 7-piperidin-1-yl-methyl derivative NSC57969 (**3**)^21^ (Table 1). Earlier work has established key features linked to the *P*-gp-potentiated activity of isatin-β-thiosemicarbazones.^47−49^ Inspired by some of these structure–activity relationships, our aim was to identify structural features mediating the MDR-selective activity of 8-hydroxyquinoline-derived Mannich bases.

**Table 1:**
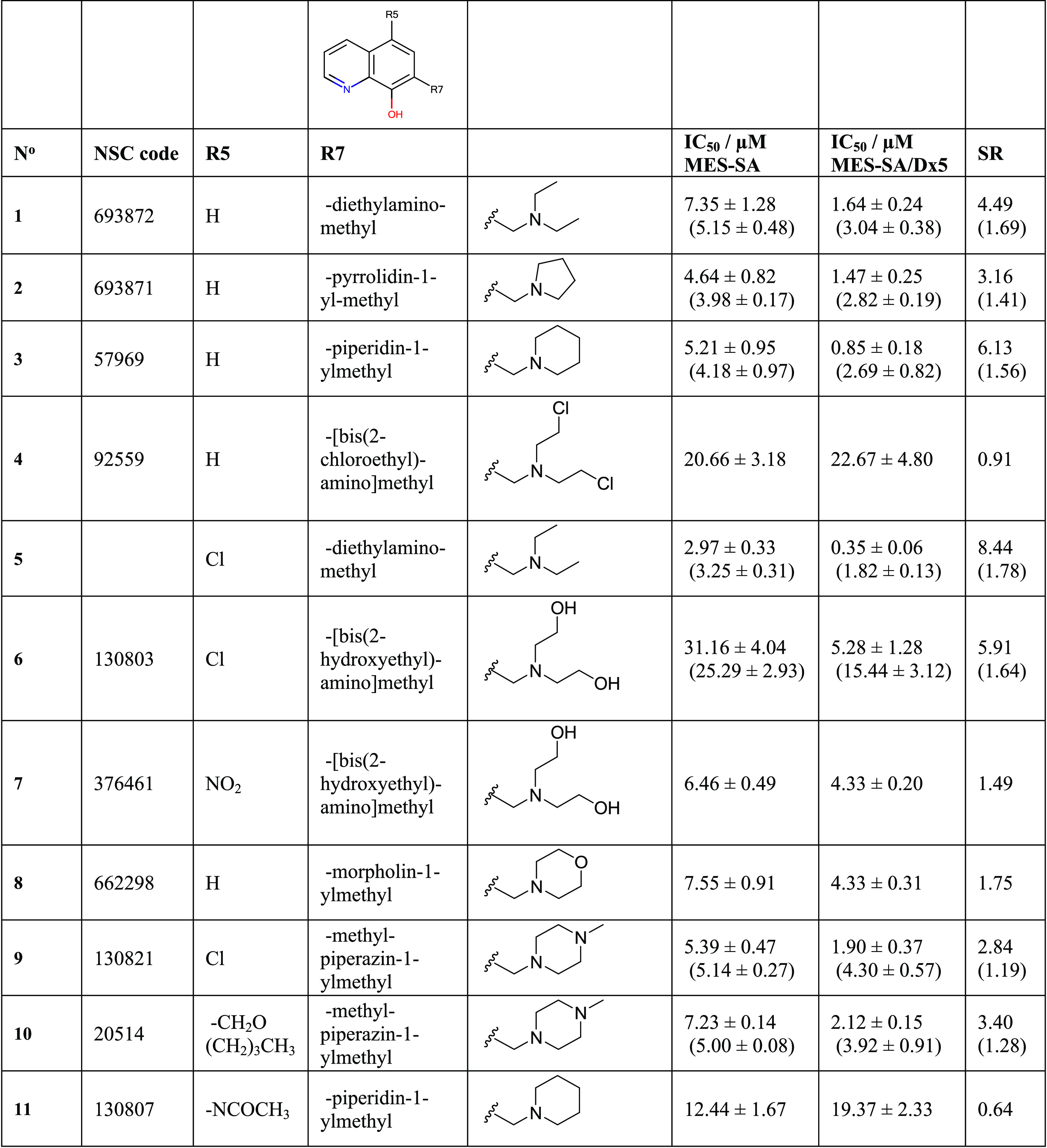
Initial SAR of 8-OHQ Derivatives Obtained from the NCI DTP Drug Repository Listed by Their NSC Codes (Commercially Available Compound **5** Is Included as a Structural Counterpart to **1**)^a^

a Data represent mean values with standard deviation obtained from 2 to 53 independent PrestoBlue assays for MES-SA and MES-SA/Dx5 cells in the absence and presence (values in parentheses) of 1 μM of the *P*-gp inhibitor tariquidar (TQ). MDR-selectivity ratio (SR) is defined as the fraction of IC_50_ values obtained in *P*-gp negative *vs* positive cells. See Table S1 for toxicity data on further MDR cell lines.

## Results and Discussion

Based on the four MDR-selective analogues identified in our earlier studies, we performed a substructure search in the DTP database retrieving 84 8-hydroxyquinoline-derived Mannich bases with an aminomethyl substituent in position R7. Six of the 13 derivatives containing a tertiary amine were available from DTP (Tables 1 and S1). To characterize MDR-selective activity, the toxicity of the compounds was tested against parental and MDR cells including the uterine sarcoma cell line MES-SA and its doxorubicin-selected MDR counterpart MES-SA/Dx5, as well as the epidermoid carcinoma cell line A431 and its derivative overexpressing *P*-gp due to retroviral infection.^46^ MDR selectivity was expressed as the ratio of IC_50_ values obtained in *P*-gp negative (parental) *vs* positive (MDR) cells (selectivity ratio (SR)). To rule out cell-specific effects and to prove that the observed MDR-selective toxicity is indeed linked to the function of the MDR efflux pump, toxicity studies were repeated in the presence of the *P*-gp inhibitor tariquidar.^15,21,46^ The small set of compounds that were made available for testing by DTP allowed preliminary structure–activity analyses. In comparison to the lead compound **1**, the introduction of chlorine atoms to the side chain ethyl groups (as represented by compound NSC92559 (**4**)) decreased toxicity and abrogated selective toxicity. In contrast, the introduction of a chlorine atom in position R5 of the scaffold (**5**) increased both toxicity and selectivity. In the presence of a chloro-substituent in R5, the introduction of the hydroxy groups to the ethyl chains did not change selectivity but decreased the overall toxicity (NSC130803, **6**). Replacement of the chloro-substituent in R5 by the even stronger electron-withdrawing nitro-group (NSC376461, **7**) slightly increased toxicity in both cell lines, however, eliminating selectivity. Similar to the results obtained with derivatives of **1**, the introduction of heteroatoms to the side chain of the highly selective **3** is detrimental, as evidenced by the decreased selectivity ratios of the morpholino-derivative NSC662298 (**8**) and the methyl-piperazino-derivatives NSC130821 (**9**) and NSC20514 (**10**) with chloro- or butoxymethyl-substituents in R5, respectively. Selective toxicity of **3** is also eliminated by the introduction of an electron-withdrawing acetamide group in position R5 (as observed in NSC130807, **11**).

To systematically analyze the validity of these initial conclusions, we compiled a focused library containing 110 commercially available and 10 newly synthesized compounds with variations at the R5 and R7 of the 8-hydroxyquinoline scaffold. The compound library was designed to study the effect of halogen and alkoxymethyl substitution in R5 in combination with different Mannich bases in R7, a shift of the Mannich base from R7 to R5, as well as an introduction of an aromatic moiety. In a disjunctive approach, we aimed to identify minimal requirements for MDR-selective activity.

### Synthesis of 8-Hydroxyquinoline-Derived Mannich Bases

**Scheme 1:**
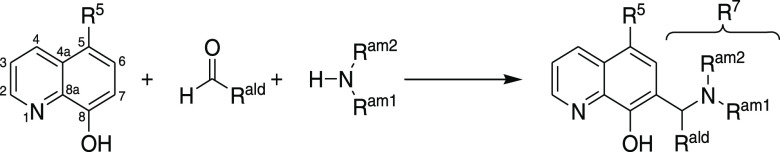
General Synthetic Scheme (ald = Aldehyde, am = Amine)

Since 8-hydroxyquinoline can be interpreted as an *N*-containing 1-naphthol analogue, its active position (C-7) can be aminoalkylated using the corresponding aldehyde and amine (Scheme 1).

### Effect of R5 and R7 Substitutions on the MDR-Selective Toxicity of 8-Hydroxyquinoline-Derived Mannich Bases

**Figure 1:**
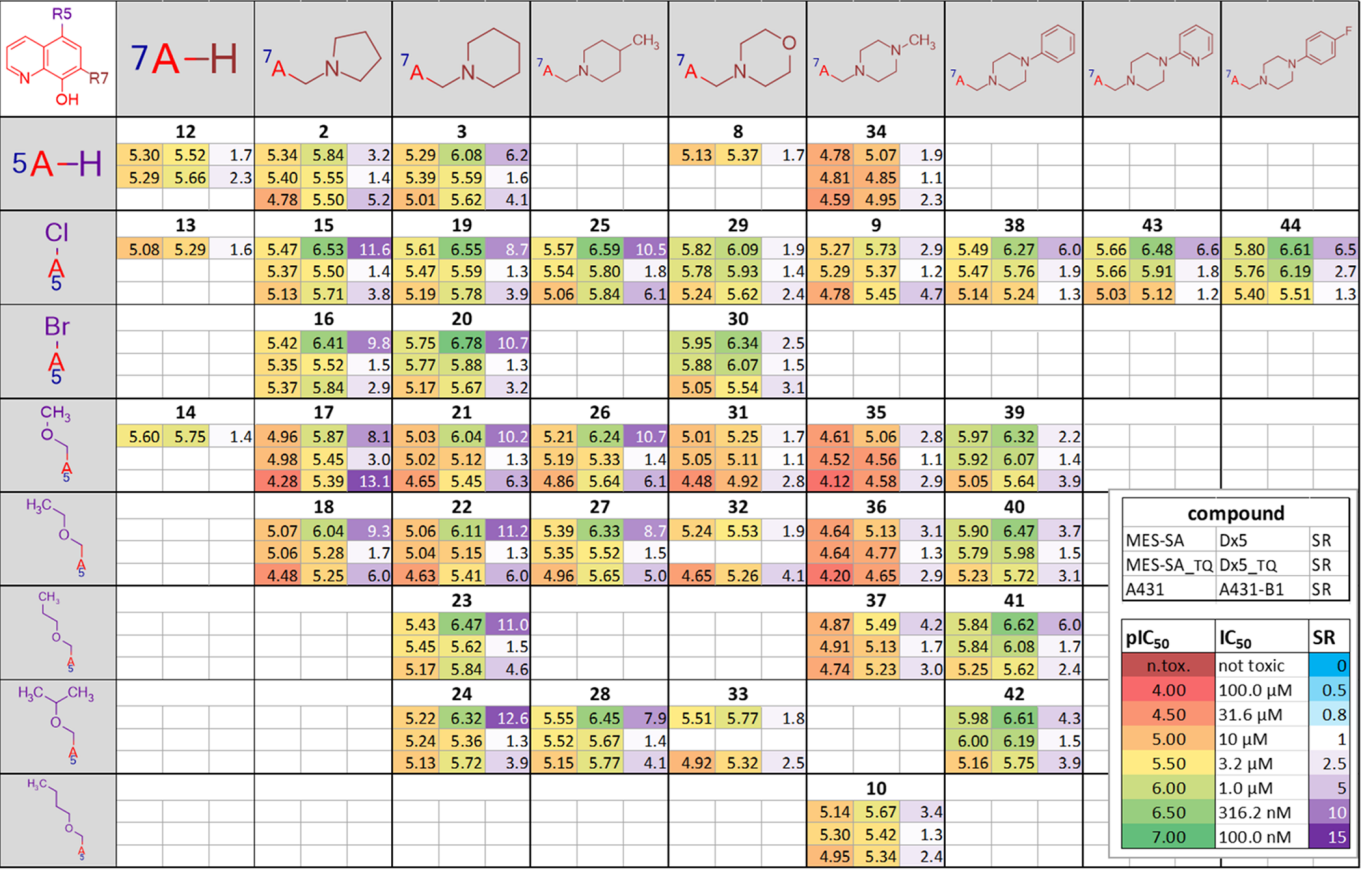
SARM of 8-hydroxyquinoline-derived Mannich bases substituted at positions R5 and R7. Each field shows the average pIC_50_ values obtained from 2 to 53 independent PrestoBlue viability assays^54^ for MES-SA and MES-SA/Dx5 cells in the absence (first row) and presence (second row) of 1 μM of the *P*-gp inhibitor TQ and for A431 and A431-B1 cells (third row). MDR-selectivity ratio (SR) is defined as the fraction of IC_50_ values obtained in *P*-gp negative *vs* positive cells. Color codes indicate toxicity and selectivity (see Table S2 for more details). The SARM figure was created using Instant J Chem for Excel.^55^

Introduction of electron-withdrawing or donating substituents has an impact on the proton dissociation constants (p*K*_a_ values) of the hydroxyl group and the quinolinium nitrogen, which were shown to be related to the iron and copper binding abilities and the MDR-selective toxicity of 8-hydroxyquinoline-derived Mannich bases.^15,50^ To evaluate the effect of R5 and R7 substitutions, a series of compounds carrying Mannich bases derived from pyrrolidine, piperidine, 4-methyl-piperidine, morpholine, and substituted piperazines in R7, with no substitution *vs* bromo-, chloro-, or alkoxy-substitution in R5, were tested in the MES-SA/MES-SA/Dx5 model,^51^ as well as in parental A431 cells and A431 cells retrovirally expressing *P*-gp.^46^ Cytotoxicity data are summarized in the structure–activity matrix (SARM) shown in Figure 1.^49,52,53^

**Figure 2:**
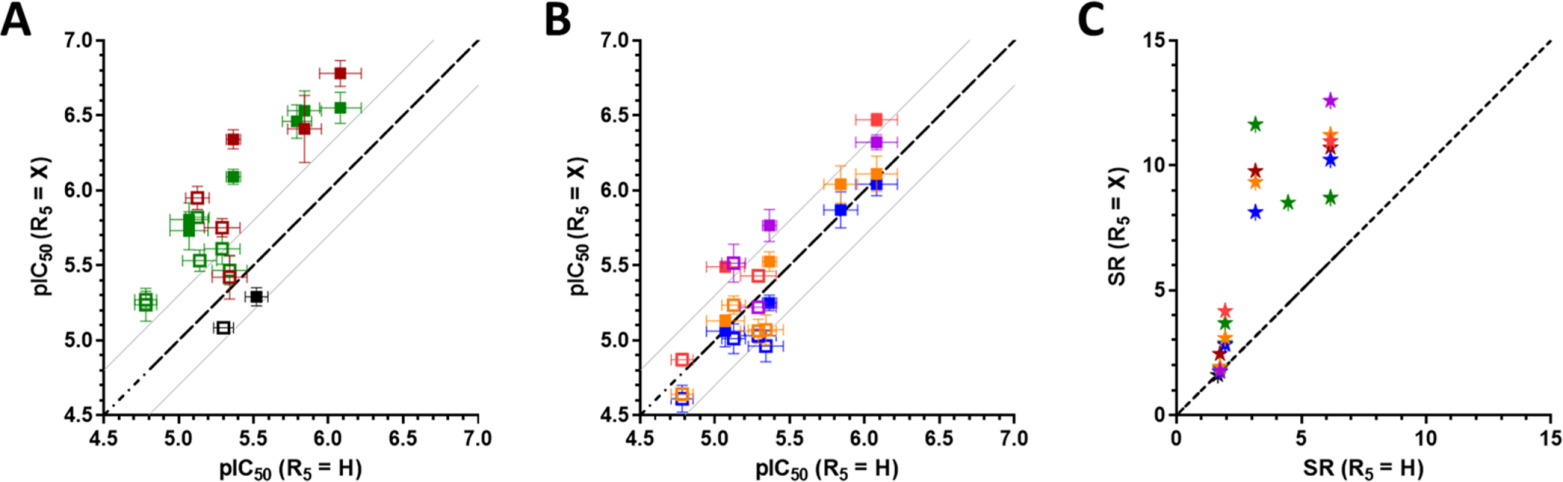
Matched molecular pairs (MMPs) showing the effect of R5 substitution on toxicity (A, B) and selectivity (C). Bisecting lines reflect values with equal potency of compounds with and without substituents in R5. Toxicity is shown as pIC_50_ values of MMPs with different substituents in R5 (substituents on *y*-axis, H on *x*-axis) against MES-SA (open symbols) and MES-SA/Dx5 cells (filled symbols). (A) Mannich bases substituted with chlorine (green), bromine (brown), and 5-chloro-substitution of the 8-OHQ scaffold (black). (B) Effect of alkoxymethyl groups −CH_2_OCH_3_ (blue), −CH_2_OCH_2_CH_3_ (orange), −CH_2_O(CH_2_)_2_CH_3_ (red), and −CH_2_OCH(CH_3_)_2_ (purple). (C) Selectivity ratios of MMPs according to the introduced color scheme.

The toxicity patterns revealed by the structure–activity matrix confirm several initial conclusions. The columns of the SARM shown in Figure 1 indicate that the MDR-selective toxicity of cyclic alkylamine derivatives bearing a pyrrolidine, piperidine, or methyl-piperidine moiety is comparable to that of the diethylamine derivatives listed in Table 1 (**1**, **5**). In contrast, the introduction of further heteroatoms, as in the case of the morpholine (**8**, **29**, **30**, **31**, **32**, **33**) and piperazine derivatives (**34**, **9**, **35**, **36**, **37**, **10**), decreases MDR-selective toxicity. Interestingly, the introduction of an additional aromatic moiety at the piperazine-nitrogen, as seen in **38**, **39**, **40**, **41**, **42**, as well as in the pyridine derivative **43** and the fluoro-substituted derivative **44**, seems to restore toxicity and partly also the selectivity of the derivatives. In agreement with the increased activity of **5** over **1** (observed in the DTP set shown in Table 1), comparison of the different rows in the SARM (Figure 1) reveals that halogen substituents in R5 increase toxicity. Interestingly, this effect is more pronounced in MDR cells, and therefore R5-halogen-substituted derivatives show increased selectivity as compared to their unsubstituted counterparts (Figure 2A,C). R5 substitution with alkoxymethyl groups (Figure 2B) decreases toxicity against MES-SA cells while modestly increasing toxicity against MES-SA/Dx5 cells (Figure 2B), therefore also resulting in an increased selectivity of the substituted derivatives (Figure 2C). Matched molecular pairs (MMPs), differing only in the substitution pattern of R5 (Figure 2) underline this observation.

**Figure 3:**
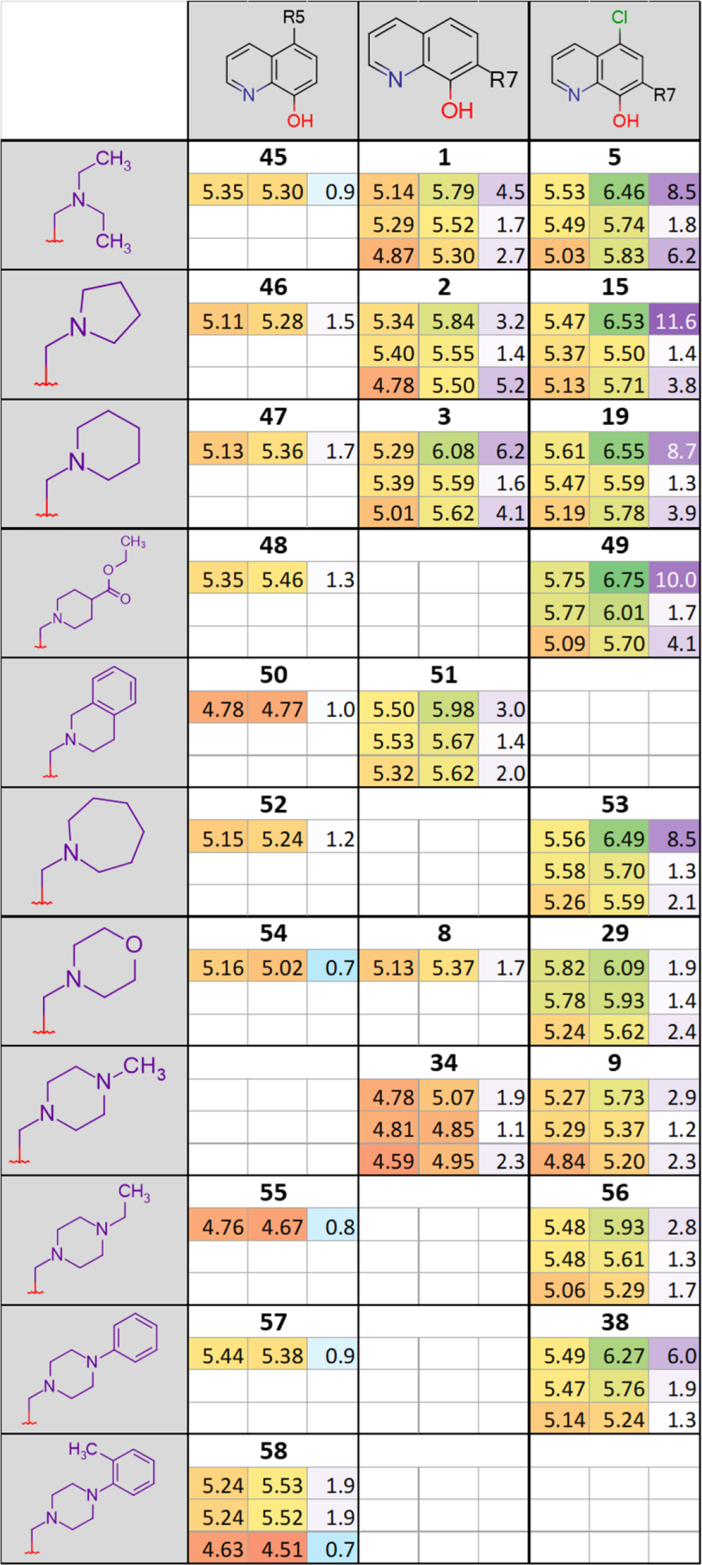
SARM^55^ of 8-hydroxyquinoline-derived Mannich bases with substitutions shifted from R7 to R5. Each field shows the average pIC_50_ values obtained from 2 to 53 independent PrestoBlue assays^54^ for MES-SA and MES-SA/Dx5 cells in the absence (first row) and presence (second row) of 1 μM of the *P*-gp inhibitor TQ and for A431 and A431-B1 cells (third row). MDR-selectivity ratio (SR) is defined as the fraction of IC_50_ values obtained in *P*-gp negative *vs* positive cells. Color codes, applied as in Figure 1, indicate toxicity and selectivity (see Table S3 for more details).

Next, we characterized derivatives, in which the substituent in R7 is shifted to the R5 position. As apparent from the SARM in Figure 3, this modification abrogates both toxicity and MDR selectivity for all 10 derivatives with this modification (**45**, **46**, **47**, **48**, **50**, **52**, **54**, **55**, **57**, **58**). However, in accordance with data shown in Figures 1 and 2, the chloro-substitution increases the toxicity and selectivity of these derivatives as well.

### Disjunctive Approach

**Figure 4:**
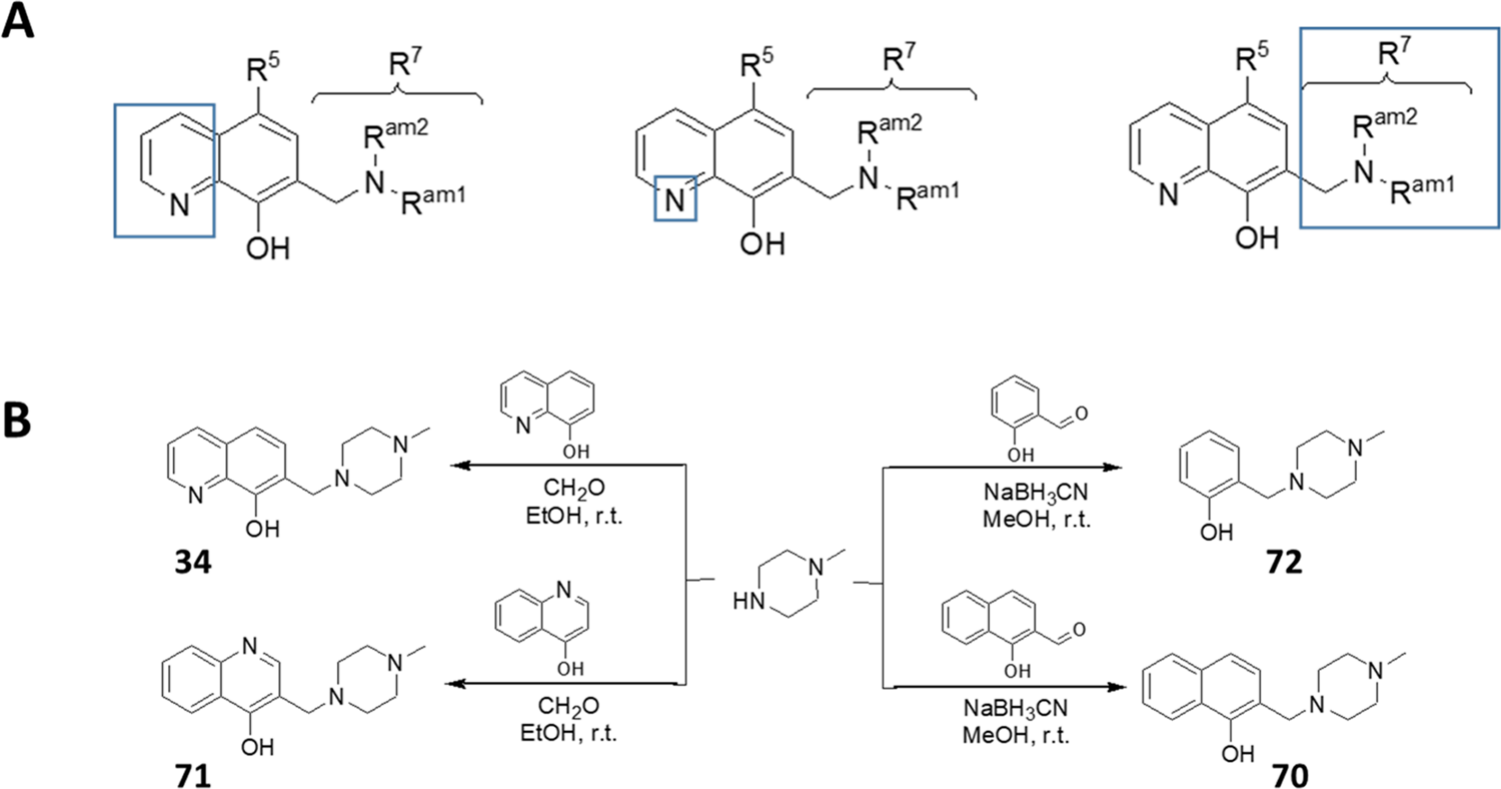
Disjunctive approach (A) and synthetic scheme (B) to obtain compounds **34** and **71** by Mannich relation or compounds **72** and **70** via reductive amination.

The results presented in Figure 3 clearly show the importance of the methylene-bridged amine residue in R7. To identify further structural requirements that are necessary for the MDR-selective toxicity of the studied 8-hydroxyquinoline-derived Mannich bases, we characterized compounds either lacking the pyridine ring of the quinoline-substructure, or the quinoline nitrogen, or the substitution in R7 (see Figure 4A), and compounds with different connectivities. The compound set compiled by this disjunctive approach^56^ contained commercially available as well as newly synthesized compounds allowing systematic comparisons. Synthesis was based on either a Mannich reaction or a reductive amination procedure, as detailed in Figure 4B.

As apparent from Table 2, the deletion of the pyridine ring from the 8-hydroxyquinoline scaffold results in the inactivation of **3**, **34**, and **8**. Due to the removal of the quinoline nitrogen from the bidentate {N,O} donor set, the phenol-derived Mannich bases (**59**, **72**, and **65**, respectively) are not able to chelate metal ions. Deletion (**70**) or shifting (**71**) of the quinoline nitrogen to the position para to the hydroxyl group reduces toxicity (as compared to **34**). Notably, the consequence is again that these derivatives are not able to form stable metal complexes. Derivatives substituted in R5 (**60**, **61**, **62**, **63**, **73**, **74**) and other nonchelating derivatives such as isoquinolin-7-ol (**64**) or naphthalen-2-ols (**69** and **75**) also lack toxicity. Interestingly, the unsubstituted 8-hydroxyquinoline core structure (**12**) and its R5-substituted derivatives **13** and **14** are not selective. Taken together, these results indicate that the presence of a chelating group is a necessary but not sufficient prerequisite for the MDR-selective toxicity of 8-hydroxyquinoline-derived Mannich bases.

**Table 2:**
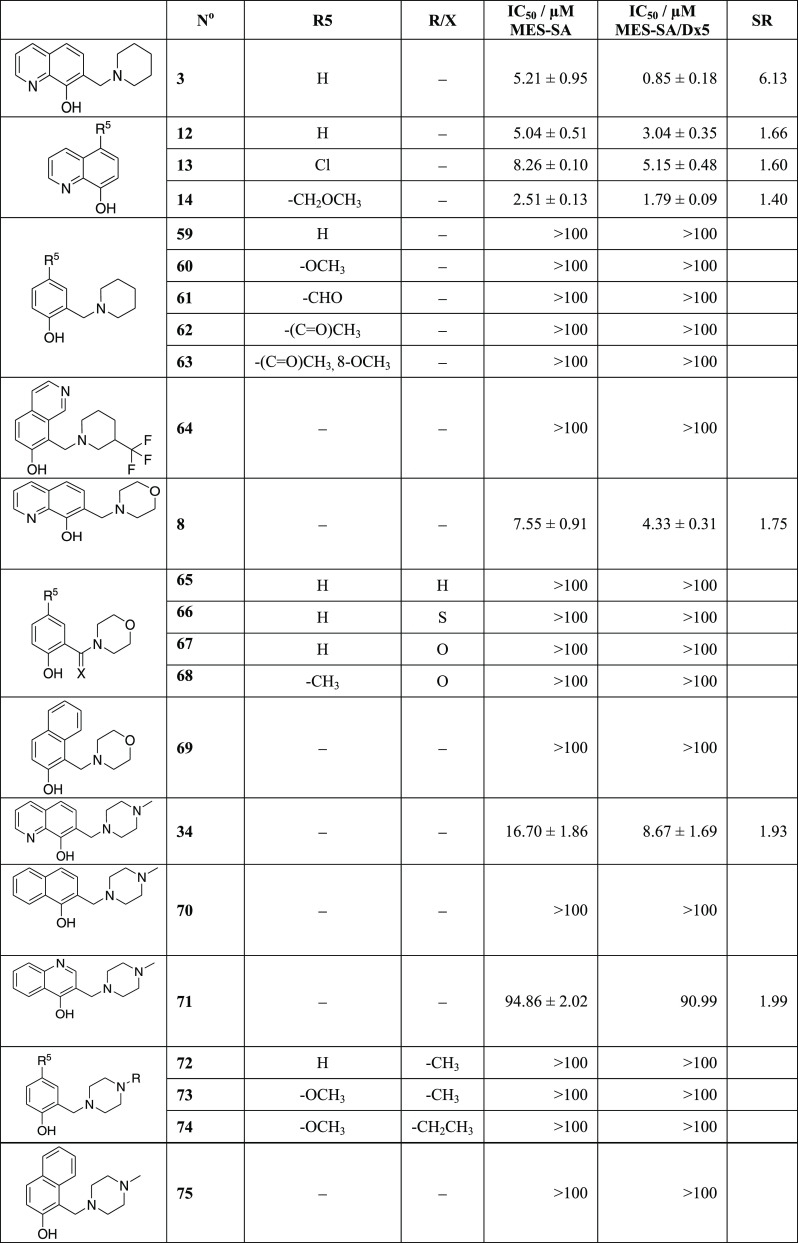
Disjunctive Approach Results in Nontoxic Derivatives^a^

a Data represent mean values with standard deviation obtained from **2** to **53** independent PrestoBlue assays for MES-SA and MES-SA/Dx5 cells in the absence and presence (values in parentheses) of 1 μM of the *P*-gp inhibitor TQ. MDR-selectivity ratio (SR) is defined as the fraction of IC_50_ values obtained in *P*-gp negative *vs* positive cells.

### Further Modifications of R7

Next, we investigated the effect of modifications at R7 by substitutions of the pyrrolidine or the piperidine rings (Table 3).

**Table 3:**
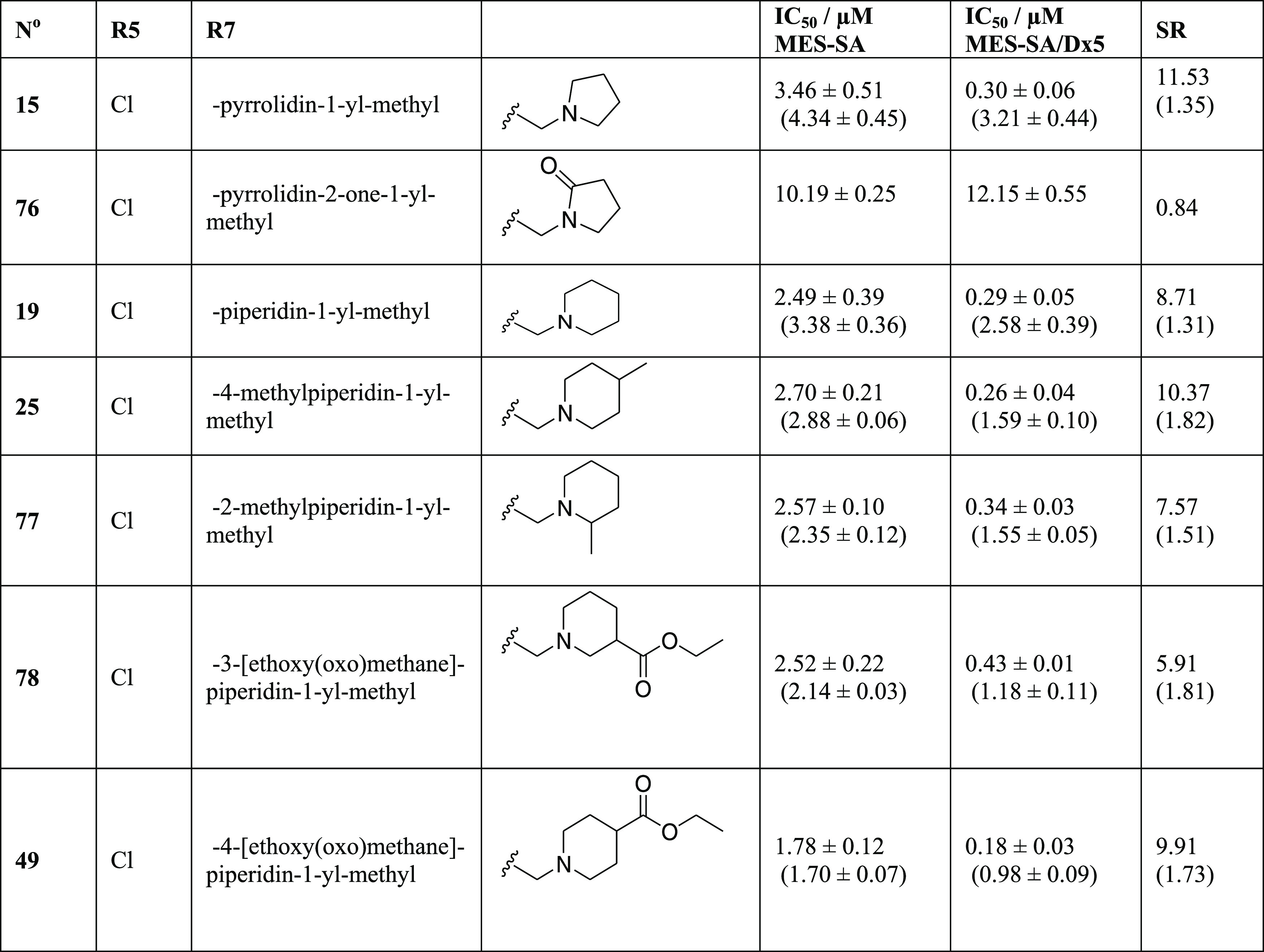
Further Derivatives with R5 Chloro-Substitution and Decorations of Pyrrolidine and Piperidine Rings in R7^a^

a Data represent mean IC_50_ values with standard deviation obtained from 2 to 10 independent PrestoBlue assays for MES-SA and MES-SA/Dx5 cells in the absence and presence (values in parentheses) of 1 μM of the *P*-gp inhibitor TQ. MDR-selectivity ratio (SR) is defined as the fraction of IC_50_ values obtained in *P*-gp negative *vs* positive cells.

Significantly lowering the basicity via amide bond formation in the pyrrolidine ring of **15** decreased toxicity and abrogated selectivity (**76**). In contrast, the introduction of an electron-donating methyl group (**25** and **77**) or of an electron-withdrawing ethyl-ester (**78** and **49**) attached to the piperidino-derivative **19** had no significant effect.

#### Introduction of an Aromatic Ring

**Scheme 2:**
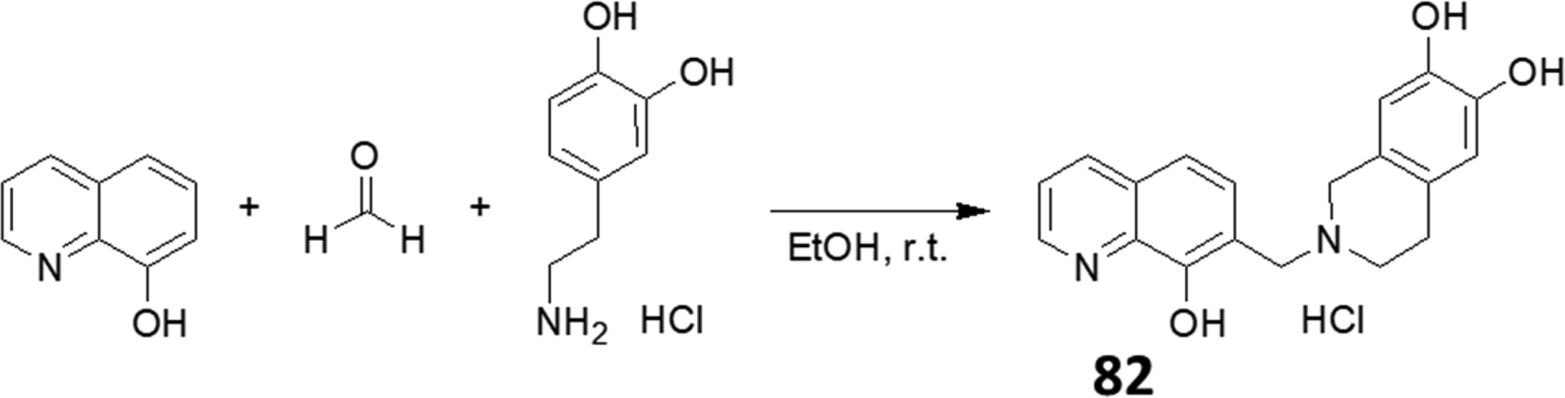
Synthesis of **82** via the Pictet–Spengler Reaction

As shown in Figure 1, the introduction of an aromatic ring to the slightly selective piperazine derivative **9** restored (selective) toxicity (**38**). To further investigate the effect of aromatic rings on the activity of the Mannich bases, we studied compounds with aromatic moieties in different distances to the 8-hydroxyquinoline core structure. **51** and **81** were synthesized starting from 1,2,3,4-tetrahydroisoquinoline and 8-hydroxyquinoline (**51**) or 5-bromo-8-hydroxyquinoline (**81**) using the standard synthetic route described above. **82** was obtained by a Pictet–Spengler condensation (Scheme 2).^57,58^

**Figure 5:**
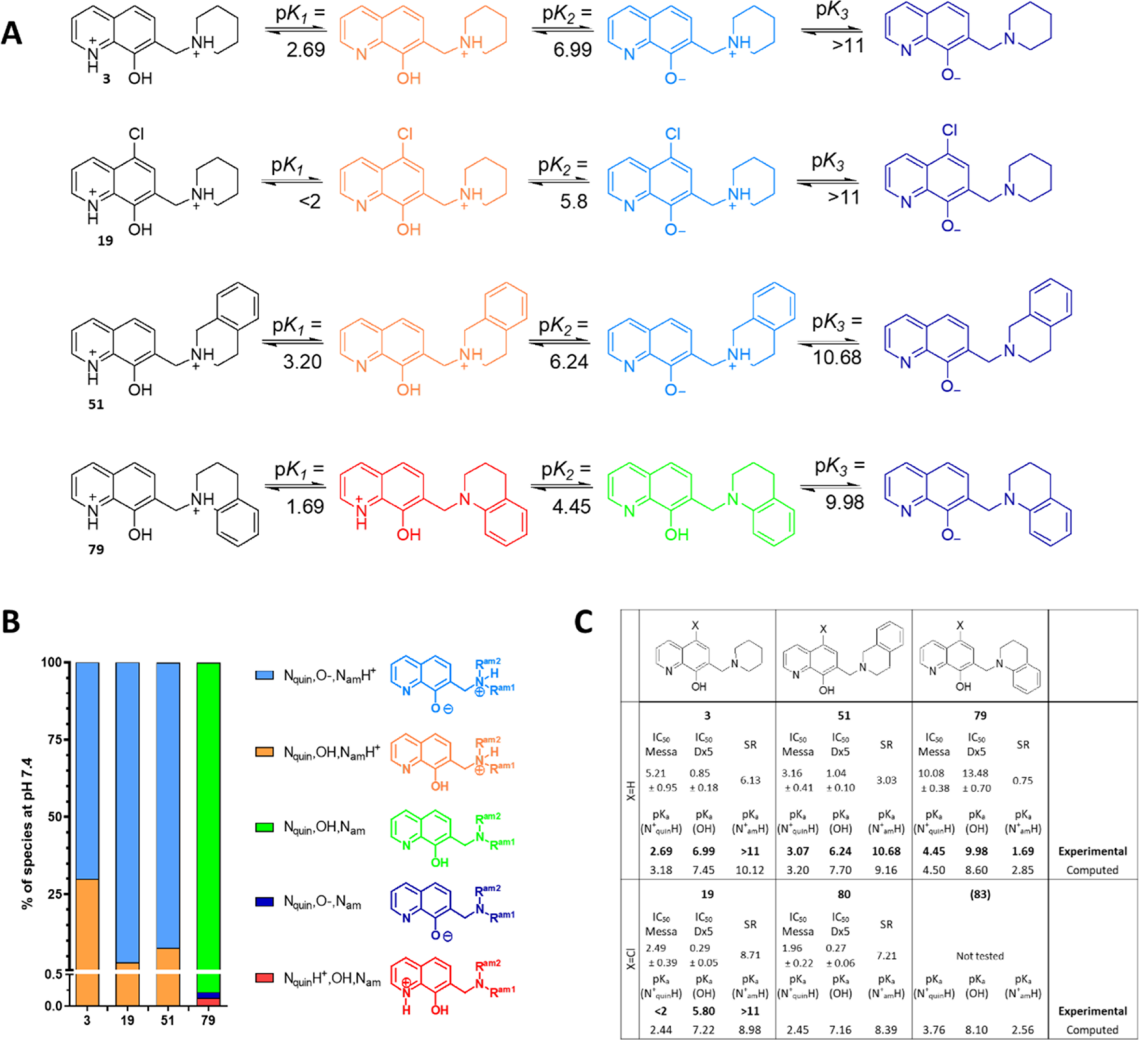
Relation of p*K*_a_ values of the donor atom moieties and MDR-selective toxicity. (A) Deprotonation processes of derivatives with annulated aromatic rings to the piperidine ring of **3** and its 5-chloro-derivative **19**. The p*K*_a_ values (with a standard deviation of ±0.03.) were determined spectrophotometrically, as described in the Experimental Section.^20,50^ (B) Distribution of species present at physiological pH, as calculated from experimental data (color code is consistent with panel A). (C) Experimentally determined and computed^55^ p*K*_a_ values are shown together with cytotoxicity data. Computed data of **80** and **83** are included to demonstrate the effect of substituents on estimated p*K*_a_ values.

The results summarized in Table 4 indicate that depending on the position and substitution pattern, annulation of the piperidine with an aromatic ring has variable effects on MDR-selective toxicity. 3,4-Benzo-piperidin-1-ylmethyl derivatives with different R5 substituents (**51**, **80**, **81**) are comparable to their respective piperidine derivatives (**3**, **19**, **20**) in terms of toxicity and selectivity. However, a dihydroxyl-substitution of the aromatic ring has detrimental effects on both toxicity and selectivity (**82**). While the 3,4-annulation of a benzene ring has only minor effects, the (selective) toxicity of 2,3-benzo-piperidin-1-ylmethyl derivative **79** (in which the aromatic moiety is in closer connectivity to the nitrogen of the Mannich base) is significantly reduced as compared to **3**. While the introduction of the aromatic rings in compounds **51**, **79**, and **80** is unlikely to cause a steric hindrance of the metal binding moiety (Figure S2A), these modifications withdraw electrons from the metal binding donor atoms. As a result, changes in the p*K*_a_ values of the donor atom moieties are to be expected, influencing metal binding properties and the anticancer activity of the ligands.^50^ To explore this relation, we determined the p*K*_a_ values of compounds **19**, **51**, and **79** by UV–vis spectrophotometry (the p*K*_a_ value of compound **3** was published earlier)^15,50^ (Figure 5A). Introduction of the aromatic ring as a 2,3-benzo-piperidine moiety has a weaker effect on p*K*_a_ values (compare **3***vs***51**) than the annulation to form a 3,4-benzo-piperidinyl derivative (compare **3***vs***79**). Furthermore, the introduction of an electron-withdrawing chloro-substituent at R5 decreases the p*K*_a_ values of the hydroxyl group as well as of the quinolinium nitrogen. This is in line with observations on the reference compound 8-hydroxyquinoline (**12**) and its 5-chloro-derivative (**13**) (experimentally determined data for **12**: p*K*_a_(N_quin_H^+^) = 4.99, p*K*_a_(OH) = 9.51 and for **13**: p*K*_a_(N_quin_H^+^) = 3.8, p*K*_a_(OH) = 7.6).^59^ In solution, compounds **3**, **19**, and **51** are mostly found in their neutral but zwitterionic form at pH 7.4 (Figure 5B). In this state, it is likely that a hydrogen bond between the phenolato oxygen and the protonated alkylamine nitrogen is present, as observed in the X-ray structure of **3**.^50^ In comparison, 2,3-benzene annulation to the piperidine ring in compound **79** had a more pronounced effect on the p*K*_a_ value of the alkylamine nitrogen, resulting in its deprotonation in the strongly acidic pH range. Consequentially, the alkylamine and quinoline nitrogens of compound **79** are deprotonated at physiological pH, while the OH group is still protonated due to its high p*K*_a_ (Figure 5B). The higher p*K*_a_ values of the OH and the quinolinium nitrogen in **79** (as compared to compounds **3**, **19**, and **51**) most likely decrease the metal binding ability via the {N,O} donor set, which might contribute to its surprisingly low SR. Intriguingly, these modifications have different consequences in parental and MDR cells, revealing an inverse correlation between p*K*_a_ values of donor atoms and MDR-selective activity (Figure 5C).^15,50^ As observed for the nonchlorinated compounds (**3***vs***51** and **79**), the introduction of an aromatic ring as 2,3-benzo-piperidine moiety in derivatives with chloro-substituent in R5 (compare **19***vs***80**) has a lower effect on p*K*_a_ values as compared to the 3,4-benzo-piperidinyl derivative (compare **19***vs***83**) (Figure 5C). Furthermore, the introduction of a chloro-substituent in R5 lowers the p*K*_a_ values of the hydroxyl group as well as that of the quinolinium nitrogen also for compounds **80** and **83** (based on the estimated p*K*_a_ values by the Marvin calculator^55^).

**Table 4:**
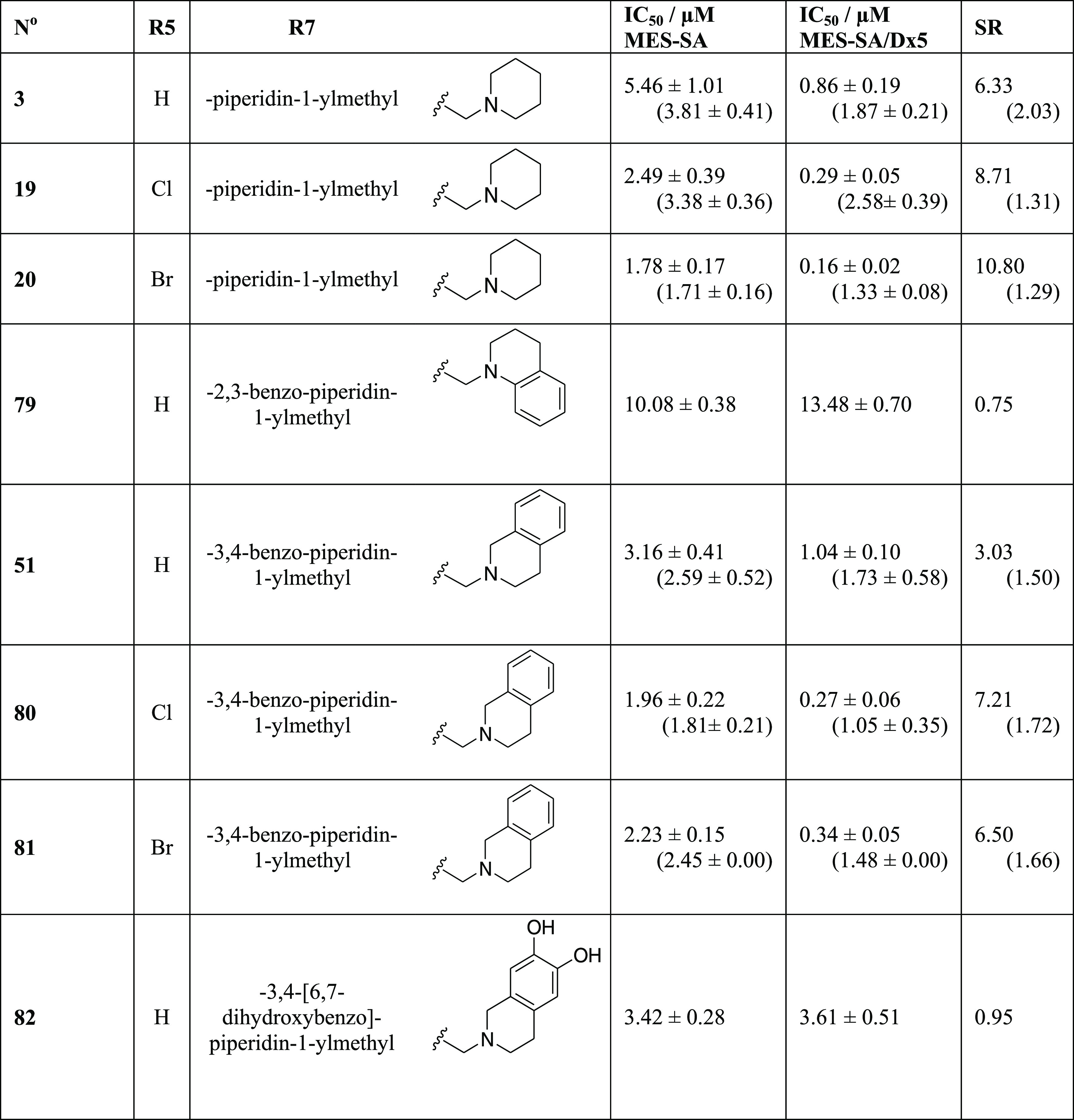
Derivatives Containing Annulated Aromatic Ring Moieties^a^

a Data represent mean IC_50_ values with standard deviation obtained from 2 to 10 independent PrestoBlue assays for MES-SA and MES-SA/Dx5 cells in the absence and presence (values in parentheses) of 1 μM of the *P*-gp inhibitor TQ. MDR-selectivity ratio (SR) is defined as the fraction of IC_50_ values obtained in *P*-gp negative *vs* positive cells.

To assess the validity of computed values, we determined the p*K*_a_ values of 17 additional compounds by UV–vis spectrometry (Table S4).

**Figure 6:**
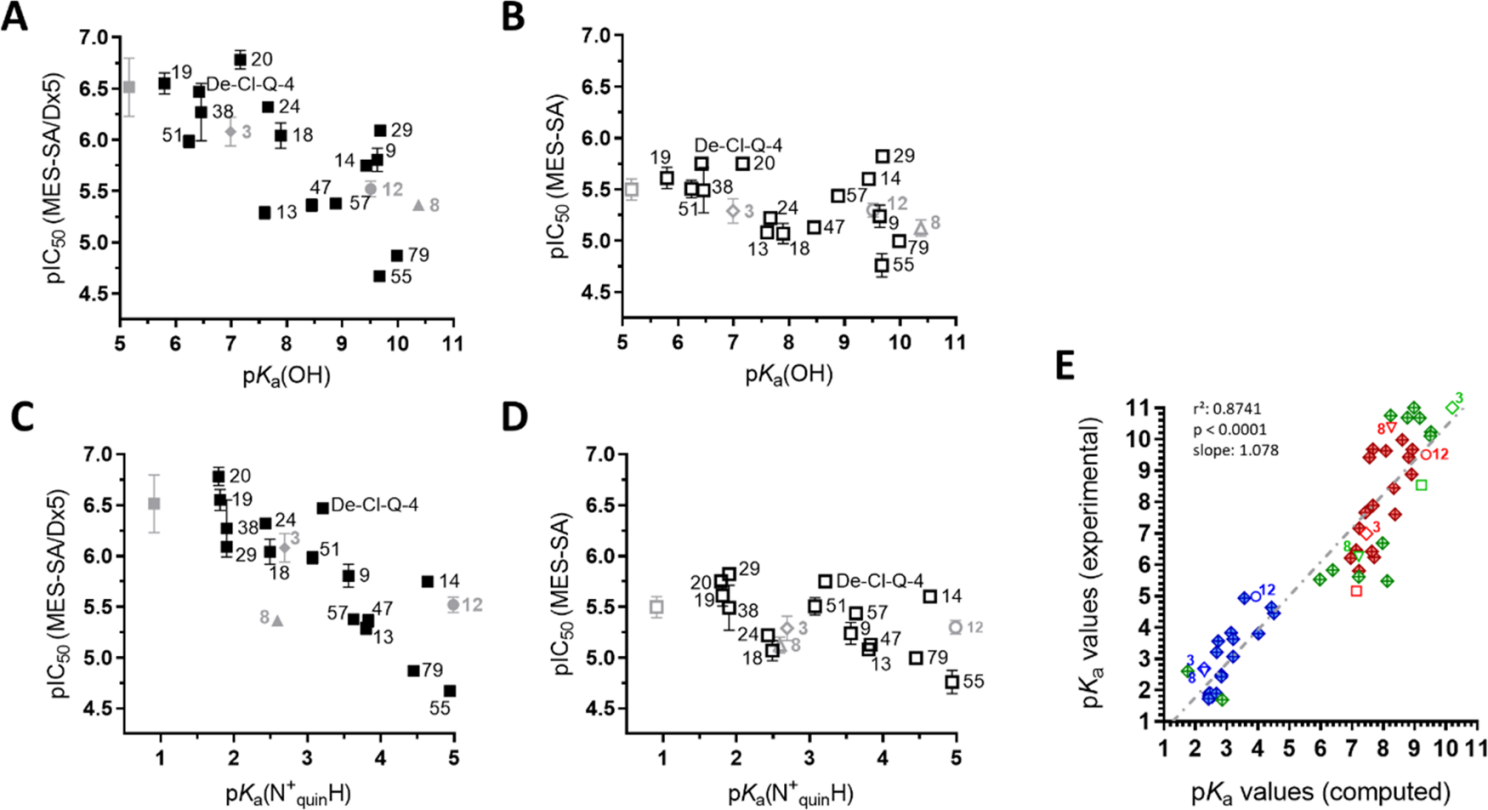
Correlation of toxicity displayed as pIC_50_ values obtained in MDR MES-SA/Dx5 (A, C, filled symbols) and parental MES-SA cells (B, D, open symbols) with p*K*_a_ values of the hydroxyl group (A, B) and the quinolinium nitrogen (C, D). Data are shown for compounds **3** (gray diamond), **8** (gray triangle), **12** (gray circle), and **Q-4** (gray squares), as determined previously,^50^ as well as from derivatives **13**,^59^**29**,^15^**De-Cl-Q-4**,^15^ and for **9**, **13**, **14**, **18**, **19**, **20**, **24**, **38**, **47**, **51**, **55**, **57**, **79** (black squares, described here). Representative spectra of the differently protonated species of compounds **38** and **9** are shown in Figure S1. (E) Correlation of experimentally determined and computed p*K*_a_ values (quinolinium nitrogen: blue, hydroxyl group: red, alkylamine moieties: green). Values indicated by open symbols and numbers are taken from reference (50).

Correlation of spectrophotometrically determined and modeled data indicates that computed p*K*_a_ values are correctly estimated (*r*^2^ = 0.87, *a* = 1.078). Slight deviations are probably due to the formation of the aforementioned hydrogen bond between the phenolato oxygen and the protonated alkylamine nitrogen,^50^ which is not taken into account by the chemoinformatic approach (a more detailed discussion is provided in the Supplementary Information). The experimental results confirm the differential effect of p*K*_a_ values on the toxicity of compounds against drug-sensitive and MDR cells. Whereas the toxicity of the compounds against *P*-gp negative MES-SA cells is largely unaffected by the different p*K*_a_ values (Figure 6B,D), multidrug-resistant MES-SA/Dx5 cells become increasingly sensitive as the p*K*_a_ values of the hydroxyl group or the quinoline nitrogen are decreased (Figure 6A,C). Deprotonation of potential donor atoms has a significant influence on the metal binding ability of ligands and the stability of the complexes.^60^ Our previous work has characterized the deprotonation and metal binding properties toward iron(III) and copper(II) of a subset of 8-OHQ derivatives with increasing MDR-selective activity (compounds **12**, **8**, **3**, and a further derivative **Q-4**).^15,50^ Based on the observed relation of deprotonation characteristics and MDR-selective toxicity (Figure 6), and a previously reported relation of donor atom p*K*_a_ values and metal binding ability,^50,60^ these results suggest that subtle differences in metal chelation properties can significantly alter the MDR-selective anticancer activity of 8-hydroxyquinoline-derived Mannich bases.

**Figure 7:**
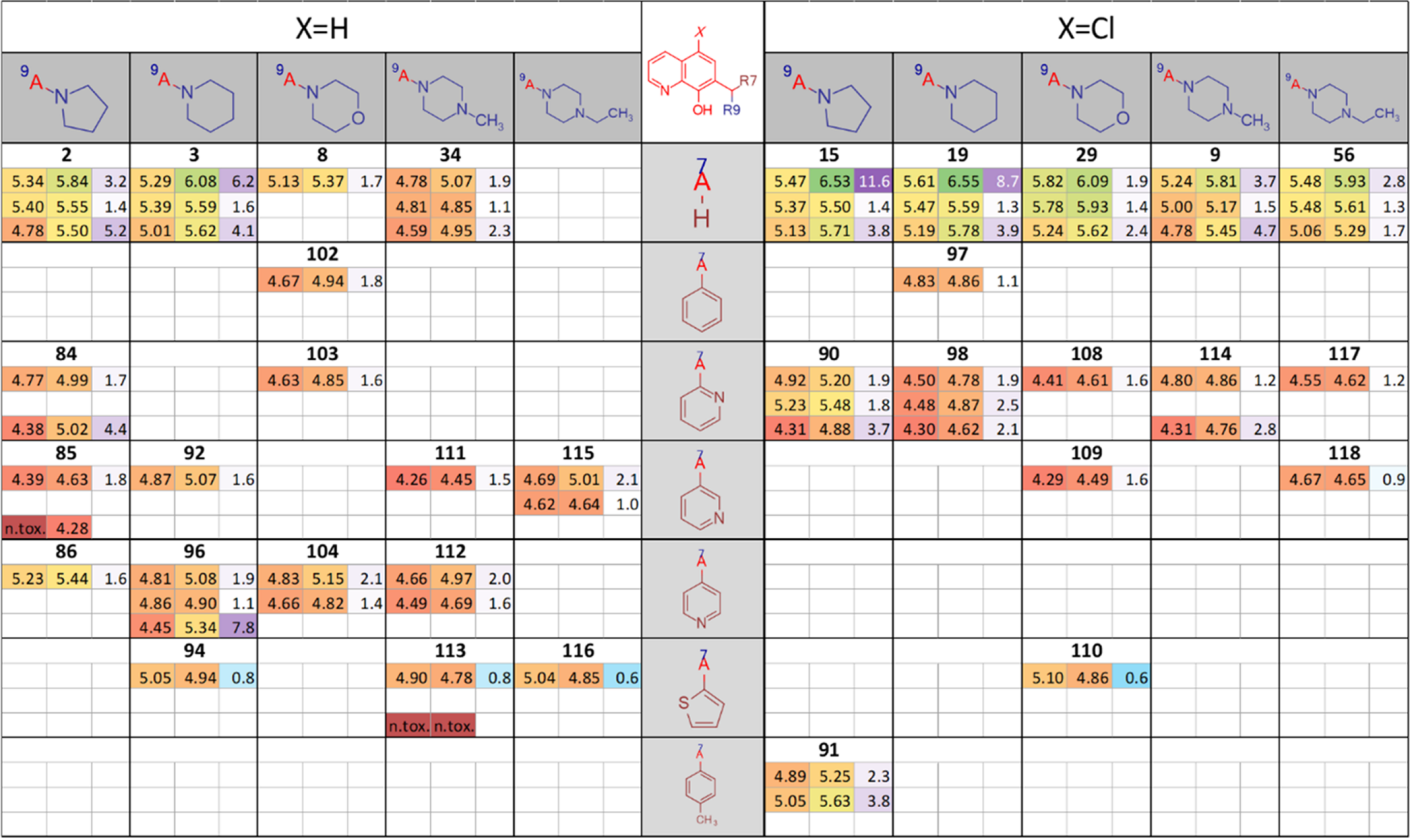
SARM^55^ comparing the effect of aromatic moieties introduced at the methylene carbon in 8-hydroxyquinoline derivatives with (right) and without (left) a chloro-substituent in R5. pIC_50_ values and selectivity ratio values are color-coded as in Figure 1. Corresponding IC_50_ values are summarized in Table S5.

Another way to introduce an aromatic moiety to the 8-OHQ scaffold is to target the methylene carbon (e.g., by the use of aromatic aldehydes in the Mannich reaction). In a series of 8-OHQ-derived HIF prolylhydroxylase inhibitors, compounds with branched aromatic substituents in R7 showed enhanced activity.^12^ However, as shown in Figure 7, this modification decreases toxicity and abrogates selectivity for derivatives with and without chloro-substitution in R5. The same effect could be confirmed by further R5-unsubstituted derivatives containing an aromatic moiety introduced at the methylene carbon (Table S5). Interestingly, for the MMPs of compounds with and without chloro-substitution in R5 that bear an aromatic ring at the methylene bridge, no clear effect of the chlorine could be observed (Figures 7 and S3).

**Figure 8:**
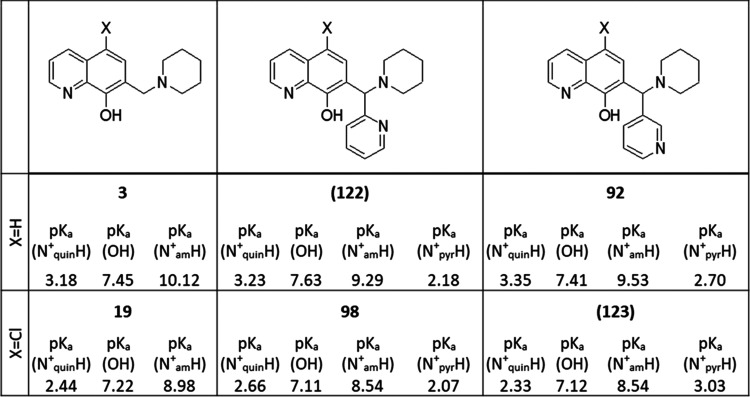
Introduction of aromatic rings to the methylene bridge of Mannich bases **3** and **19**. Calculated p*K*_a_ values of the heteroatoms of derivatives with (**19**, **98**, (**123**)) and without (**3**, (**122**), **92**) chloro-substituents in R5 (compounds (**122**) and (**123**) were not tested in cytotoxicity experiments).

Due to the closer proximity to the chelating moiety, an aromatic ring at the methylene bridge has a larger impact on the steric hindrance of the 8-hydroxyquinoline core structure, as compared to the ring annulation (Figure S2B). Interestingly, based on calculated p*K*_a_ values, the effect of the aromatic ring introduced to the methylene carbon is smaller as compared to that of ring annulation (Figure 8; compare **3***vs* (**122**) and *vs***92**, as well as **19***vs***98** and *vs* (**123**)). We experimentally determined the p*K*_a_ values of two derivatives with aromatic substitution at methylene carbon (compounds **97** and **108**; see Table S4 and Figure S4).

### Impact of Chemical Properties

**Figure 9:**
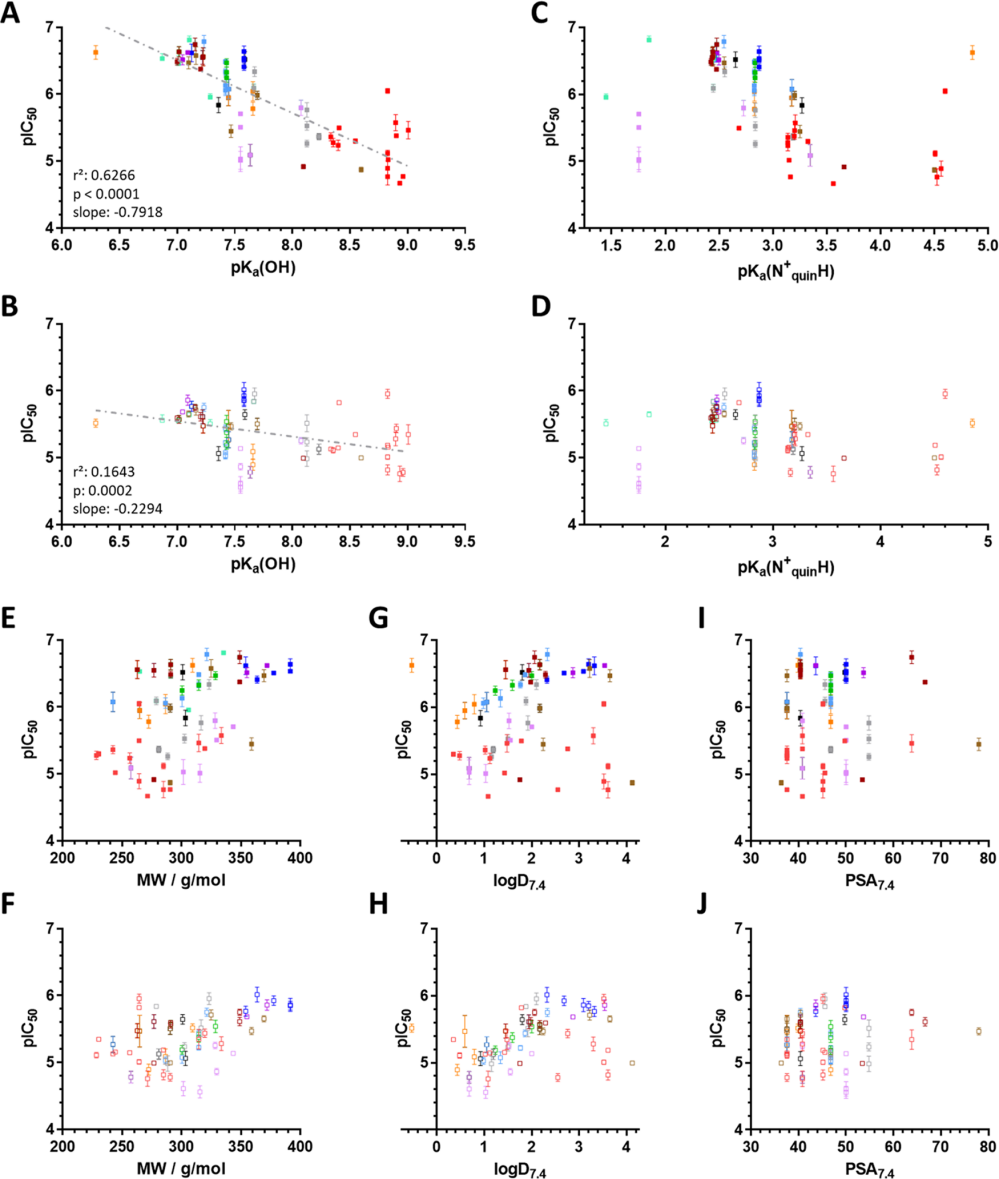
Impact of the computed^55^ chemical properties p*K*_a_(OH) (A, B), p*K*_a_(N_quin_^+^H) (C, D), molecular weight MW (E, F), log *D* at pH 7.4 (G, H), and the polar surface area at pH 7.4 (I, J) on the toxicity profile of 79 8-hydroxyquinoline derivatives against MES-SA/Dx5 (A, C, E, G, I) and MES-SA (B, D, F, H, J) cells. Linear correlation coefficients are shown in panels (A) and (B). Color coding distinguishes the following compound classes: R5R7-substituted derivatives from SARM (Figure 1): 7-pyrrolidenyl-methyl derivatives (orange), 7-piperidinyl-methyl derivatives (light blue), 7-(4-methyl-piperazin)-1-yl-methyl derivatives (green), 7-morpholinyl-methyl derivatives (light purple), 7-(4-phenyl-piperazin)-1-yl-methyl derivatives (blue), substituted 7-(4-methyl-piperazin)-1-yl-methyl derivatives (purple), 7-tetrahydroisoquinolinyl-methyl derivatives (light brown), and 7-pyrrolidenyl- and 7-piperidinyl-methyl derivatives with further ring decoration (bordeaux). Derivatives with R5 substitution only (red), with R7 substitutions (black), and with R7 substitutions and a chloro-substituent in R5 (cyan).

To systematically investigate the influence of acid–base properties on MDR-selective toxicity, p*K*_a_ values were calculated for all compounds involved in this study. As compared to the parental cells, MES-SA/Dx5 cells are more sensitive to changes in the calculated p*K*_a_ values of the hydroxyl group (compare slopes in Figure 9A,B), indicating that the acid–base and metal-chelating properties are important factors modulating the MDR-selective anticancer activities of 8-hydroxyquinoline-derived Mannich bases (Figure 9A–D). Interestingly, compounds in which the substituent is shifted from R7 to R5 (displayed in red) show the highest calculated p*K*_a_ values and the lowest selective toxicity. A similar, yet less pronounced trend is observed for the p*K*_a_ values of the quinolinium nitrogen (Figure 9C,D). In contrast, other chemical properties, such as molecular weight (Figure 9E,F), distribution coefficient (log* D*; Figure 9G,H), and polar surface area (Figure 9I,J) at physiological pH, did not show such clear trends, suggesting that these properties are not main drivers of the MDR-selective toxicity of 8-hydroxyquinoline-derived Mannich bases. These results also imply that the detrimental effect of an aromatic moiety in the methylene group (as demonstrated by the examples in Figure 7 and Table S5) cannot be explained by the alteration of the calculated chemical properties (Figure S5).

## Conclusions

**Figure 10:**
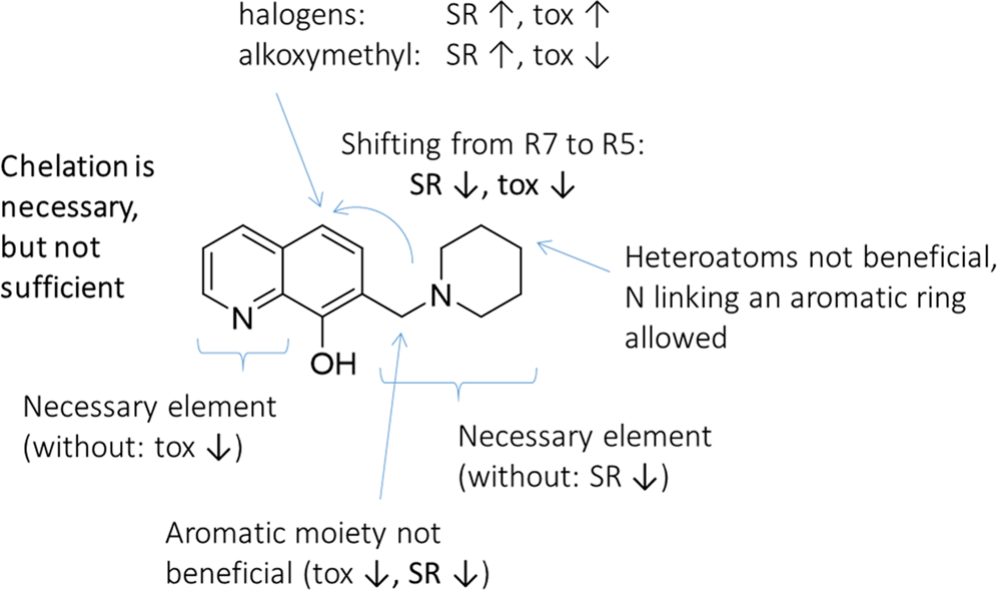
Conclusions of the study.

Our recent work has identified several 8-hydroxyquinoline-derived Mannich bases with increased toxicity against a panel of MDR cells. Here, our aim was to explore the chemical space around previously identified MDR-selective derivatives NSC693871, NSC693872, and NSC57969 by characterizing the MDR-selective toxicity of a library consisting of 120 derivatives. The conclusions are summarized in Figure 10. We find that metal chelation is necessary but not sufficient for MDR-selective activity. A reproducible increase of MDR selectivity could be achieved by the introduction of diverse substituents in R5, including halogens that increase both toxicity and selectivity, and alkoxymethyl groups that increase selectivity but decrease toxicity. Shifting the methylene-bridged amine from R7 to R5 results in less toxic and nonselective derivatives. We find that heteroatoms introduced to the alkyl-amines in R7 disrupt selectivity, which can, however, be restored by the introduction of an aromatic ring to piperazine derivatives. The effect of aromatic ring annulation on a piperidine ring strongly depends on connectivity. While derivatives with aromatic rings in the α,β-position to the amine (resulting in a 2,3-benzo-piperidin-1-ylmethyl residue) are less toxic and lose selectivity, β,δ-annulation (resulting in 3,4-benzo-piperidin-1-ylmethyl derivatives) does not reduce MDR-selective activity. In contrast, the introduction of an aromatic ring at the methylene bridging carbon diminishes toxicity and selectivity. The observed trends in this structure–activity relationship can be explained by changes in the p*K*_a_ values of the donor atom moieties. Correlations shown in Figures 9 and S4 confirm a recently suggested trend that was based on measurements performed with four derivatives,^15^ indicating that the acid–base properties and metal-chelating ability are important factors modulating the MDR-selective anticancer activities of 8-hydroxyquinoline-derived Mannich bases. Taken together, our results identify structural requirements increasing the toxicity and MDR-selective activity of 8-hydroxyquinoline-derived Mannich bases, providing guidelines for the development of more effective anticancer chelators targeting MDR cancer.

## Experimental Section

### Synthesis

#### Materials and Methods

All reagents and solvents purchased from commercial vendors were used without further purification. Concentration of reaction mixtures refers to rotary evaporation under reduced pressure carried out at 40 °C. Thin-layer chromatography (TLC) was performed on Merck Silica gel 60 F_254_-precoated TLC plates (0.25 mm thickness) and visualized at 254 nm. Silica gel flash chromatography was performed using silica gel (0.040–0.063 mm) from Merck. NMR spectral data were obtained at ambient temperature unless otherwise specified. ^1^H (^13^C) NMR spectra were recorded at 300 (**75**) or 500 (**125**) MHz (Instrument: Varian UNITY-INOVA 300 MHz, Varian INOVA 500 MHz or Bruker DRX-500 spectrometer) in CDCl_3_ or DMSO-*d*_6_. Chemical shifts are reported and shown in parts per million (ppm) and referenced against CDCl_3_ (7.26 ppm for ^1^H and 77.0 ppm for ^13^C) or DMSO (2.50 ppm for ^1^H and 39.5 ppm for ^13^C). Melting points were measured by the OptiMelt Automated Melting Point System or by a Hinotek X-4 melting point apparatus and are uncorrected.

Purity of all compounds was ≥95% as determined by HPLC-MS, using an AB Sciex 3200QTrap tandem mass spectrometer and a PS Series200 HPLC system. Ionization mode: ESI in positive ion mode. Column: Kinetex C18, 150 mm × 4.6 mm 5 μm. UV: 254 nm. Mobile phase: A: 0.1% formic acid in water, B: 0.1% formic acid in acetonitrile. Flow rate: 0.6 mL/min. Preparative reversed phase HPLC was performed on a Waters Sunfire column (19 mm × 50 mm, C18, 5 μm) with a 10 min mobile phase gradient of 10% acetonitrile/water to 90% acetonitrile/water with 0.1% TFA as buffer using 214 and 254 nm as detection wavelengths.

Chemical properties p*K*_a_(OH), p*K*_a_(N_quin_^+^H), molecular weight, log* D* at pH 7.4, and polar surface area at pH 7.4 were calculated with Marvin calculator from ChemAxon (https://www.chemaxon.com).^55^

##### 5-Bromo-7-(pyrrolidin-1-ylmethyl)quinolin-8-ol (**16**)

A solution of pyrrolidine (91 μL, 0.078 g, 1.1 mmol) and 37% formaldehyde (40 μL, 0.033 g, 1.1 mmol) was stirred for 1 h prior to the addition of 5-bromo-8-hydroxyquinoline (0.224 g, 1 mmol, in 4 mL ethanol) and subsequent reaction at room temperature for 4 days. Upon removal of the solvent in vacuo, the crude product was dissolved in dichloromethane and washed with 10% NaOH solution (1×), brine, and water. The organic phase was dried over Na_2_SO_4_, concentrated under reduced pressure, and washed with cold ethanol. Compound **16** was isolated as green crystals in a yield of 18% (0.05 g). Mp 119–121 °C. ^1^H NMR (500 MHz, CDCl_3_; Figure S10) δ = 1.88 (s, 4H, CH_2_-N-(CH_2_-C*H*_2_)_2_), 2.70 (s, 4H, CH_2_-N-(C*H*_2_-CH_2_)_2_), 3.98 (s, 2H, C*H*_2_-N-(CH_2_-CH_2_)_2_), 7.47 (dd, *J* = 8.4 *Hz*, 4.0 *Hz*, 1H_ar_, *H-*3), 7.52 (s, 1H_ar_, *H-*6), 8.41 (d, *J* = 8.4 *Hz*, 1H_ar_, *H-*4), 8.87 (d, *J* = 3.1 *Hz*, 1H_ar_, *H-*2), 10.18 (br s, 1H, OH). ^13^C NMR (126 MHz, CDCl_3_; Figure S11) δ = 23.82 (2 C_aliphatic_, pyrrolidin: CH_2_-N-(CH_2_-*C*H_2_)_2_), 53.88 (2 C_aliphatic_, pyrrolidin: CH_2_-N-(*C*H_2_-CH_2_)_2_), 57.51 (*C*H_2_-N-(CH_2_-CH_2_)_2_), 109.24 (C_q,ar_, *C-*5), 120.11 (C_q,ar_, *C-*4*a*), 122.33 (C-H_ar_, *C-*3), 127.37 (C_q,ar_, *C-*7), 130.61 (C-H_ar_, *C-*6), 135.35 (C-H_ar_, *C-*4), 140.23 (C_q,ar_, *C-*8*a*), 149.40 (C-H_ar_, *C-*2), 153.45 (C_q,ar_, *C-*8). LCMS RT = 4.04 min, HPLC shown in Figure S8. ESI^+^*m*/*z*: 307.04 [M + H^+^].

##### 5-Bromo-7-(piperidin-1-ylmethyl)quinolin-8-ol (**20**)

Compound **20** was synthesized according to reference (61) and isolated as green crystals with a yield of 31%. NMR data are in accordance with those published in reference (61). Mp 122–123 °C. ^1^H NMR (500 MHz, CDCl_3_; Figure S12): δ = 1.51 (s, 2H, CH_2_-N-(CH_2_-CH_2_)_2_C*H*_2_), 1.72–1.63 (m, 4H, CH_2_-N-(CH_2_-C*H*_2_)_2_CH_2_), 2.59 (s, 4H, CH_2_-N-(C*H*_2_-CH_2_)_2_CH_2_), 3.83 (s, 2H, C*H*_2_-N-(CH_2_-CH_2_)_2_CH_2_), 7.44–7.48 (m, 2H_ar_, *H-*6, *H-*3), 8.41 (dd, *J* = 8.5, 1.4 Hz, 1H_ar_, *H-*4), 8.89 (dd, *J* = 4.0, 1.3 Hz, 1H_ar_, *H-*2). ^13^C NMR (126 MHz, CDCl_3_; Figure S13) δ = 23.02 (CH_2_-N-(CH_2_-CH_2_)_2_*C*H_2_), 24.90 (CH_2_-N-(CH_2_-*C*H_2_)_2_CH_2_), 53.21 (CH_2_-N-(*C*H_2_-CH_2_)_2_CH_2_), 60.16 (*C*H_2_-N-(CH_2_-CH_2_)_2_CH_2_), 108.26 (C_q,ar_, *C-*5), 117.90 (C_q,ar_, *C-*4*a*), 121.35 (C-H_ar_, *C-*3), 126.47 (C_q,ar_, *C-*7), 129.77 (C-H_ar_, *C-*6), 134.32 (C-H_ar_, *C-*4), 139.35 (C_q,ar_, *C-*8*a*), 148.51 (C-H_ar_, *C-*2), 152.99 (C_q,ar_, *C-*8).

##### 7-((4-Methylpiperazin-1-yl)methyl)quinolin-8-ol (**34**)

A solution of 1-methyl-piperazine (573 μL, 0.517 g, 5.16 mmol) and 37% formaldehyde (465 μL, 0.384 g, 4.47 mmol) in ethanol (5 mL) was stirred for 1 h prior to the addition of 8-hydroxyquinoline 0.5 g, 3.44 mmol, in 5 mL ethanol. The mixture was stirred at room temperature for 12 h. Upon solvent removal, the crude product was taken up with dichloromethane and washed with 10% NaOH solution (1×), brine, and water and purified by flash chromatography (silica gel, eluent: CH_2_Cl_2_/CH_3_OH = 96:4). Compound **34** was isolated as white crystals (0.41 g, 46% yield). Mp. 120–122 °C. ^1^H NMR (500 MHz, CDCl_3_; Figure S14) δ = 2.28 (s, 3H, C*H*_3_), 2.51 (br s, 4H, CH_2_-N-(CH_2_-C*H*_2_)_2_-NCH_3_), 2.65 (br s, 4H, CH_2_-N-(C*H*_2_-CH_2_)_2_-NCH_3_), 3.87 (s, 2H, C*H*_2_-N-(CH_2_-CH_2_)_2_-NCH_3_), 7.18–7.25 (m, 2H_ar_, *H-*3, *H-*6), 7.34 (dd, *J* = 8.2 *Hz*, 4.1 *Hz*, 1H_ar_, *H-*4), 8.04 (d, *J* = 8.0 *Hz*, 1H_ar_, *H-*5), 8.85 (d, *J* = 3.0 *Hz*, 1H_ar_, *H-*2), 11.20 (br s, 1H, O*H*). ^13^C NMR (126 MHz, CDCl_3_; Figure S15) δ = 46.04 (*C*H_3_), 52.82 (2 aliphatic CH_2_: CH_2_-N-(*C*H_2_-CH_2_)_2_-NCH_3_), 55.09 (2 aliphatic CH_2_: CH_2_-N-(CH_2_-*C*H_2_)_2_-NCH_3_), 60.53 (*C*H_2_-N-(CH_2_-CH_2_)_2_-NCH_3_), 117.47 (C-H_ar_, *C-*5), 117.92 (C_q,ar_, *C-*7), 121.30 (C-H_ar_, *C-*3), 127.73 (C-H_ar_, *C-*6), 128.54 (C_q,ar_, *C-*4*a*), 135.69 (C-H_ar_, *C-*4), 139.41 (C_q,ar_, *C-*8*a*), 148.99 (C-H_ar_, *C-*2), 153.25 (C_q,ar_, *C-*8). LCMS RT = 1.23 min. HPLC shown in Figure S7. ESI^+^*m*/*z*: 258.2 [M + H^+^].

##### 7-((3,4-Dihydroisoquinolin-2(1*H*)-yl)methyl)quinolin-8-ol (**51**)

A mixture of 1,2,3,4-tetrahydroisoquinoline (148 μL, 0.156 g, 1.177 mmol), 8-hydroxyquinoline (0.172 g, 1.177 mmol), and 37% formaldehyde (55 μL, 0.043 g, 1.49 mmol) in ethanol (10 mL) was stirred at room temperature for 1 day. The solvent was removed in vacuo, and the residue was crystallized with Et_2_O (12 mL) and recrystallized with *i*-Pr_2_O (10 mL). The titled compound was isolated as white crystals. (0.232 g, 68%). Mp 155–157 °C. ^1^H NMR (500 MHz, CDCl_3_; Figure S16) δ = 2.91–3.06 (m, 4H, C*H*_2_*-*3′, C*H*_2_-4′), 3.86 (s, 2H, C*H*_2_*-1*′), 4.08 (s, 2H, C*H*_2_-N-(CH_2_-CH_2_)_2_-N), 7.00 (d, *J* = 7.2 Hz, 1H_ar_, *H-*7′), 7.11–7.17 (m, 3H_ar_, *H-*5′, *H-*6′, *H-*8′), 7.30 (d, *J* = 8.3 Hz, 1H_ar_, *H-*6), 7.35–7.42 (m, 2H_ar_, *H-*3, *H-*5), 8.11 (d, *J* = 8.0 Hz, 1H_ar_, *H-*4), 8.86 (br s, 1H_ar_, *H-*2). ^13^C NMR (126 MHz, CDCl_3_; Figure S17) δ = 28.3 (CH_2_, *C-*4′), 50.36 (CH_2_, *C-1*′), 55.63 (CH_2_, *C-*3′), 59.94 (Ar_8OHQ_-*C*H_2_-N), 117.68 (C-H_ar_, *C-*5), 117.94 (C_q,ar_, *C-*7), 121.53 (C-H_ar_, *C-*3), 125,11 (C_q,ar_, *C-*4*a*), 126.05 (C-H_ar_, *C-*7′), 126.36 (C_q,ar_, *C-*8′*a*), 126.69 (C-H_ar_, *C-*6′), 128.36 (C-H_ar_, *C-*5′), 128,58 (C_q,ar_, *C-*8*a*), 135.88 (C-H_ar_, *C-*4), 139.28 (C_q,ar_, *C-*4*a*), 148.95 (C-H_ar_, *C-*2), 152.98 (C_q,ar_, *C-*8).

##### 2-((4-Methylpiperazin-1-yl)methyl)naphthalen-1-ol (**70**)

HC1 (0.4 mL of 5 N) in methanol (1:1) was added to a solution of 1-methyl-piperazine (554 μL, 0.500 g, 5.00 mmol) in methanol (30 mL), followed by 1-hydroxy-2-naphthaldehyde (0.172 g, 1.00 mmol) in 10 mL of methanol. The solution was stirred under a nitrogen atmosphere for 10 min before solid sodium cyanoborohydride (62.8 mg, 1.00 mmol) was added, and the solution was stirred overnight at room temperature. The mixture was evaporated, and the residue was purified by column chromatography (silica gel, eluent: EtOAc/CH_3_OH = 2:1). The product was isolated as beige crystals (0.16 g, 62%). Mp 79–81 °C. ^1^H NMR (500 MHz, CDCl_3_; Figure S18) δ = 2.33 (s, 3H, C*H*_3_), 2.68 (br s, 8H, CH_2_-N-(C*H*_2_-C*H*_2_)_2_-NCH_3_), 3.87 (s, 2H, C*H*_2_-N-(CH_2_-CH_2_)_2_-NCH_3_), 7.08 (d, *J* = 8.3 Hz, 1H_ar_, *H-*3), 7.29 (d, *J* = 8.3 Hz, 1H_ar_, *H-*4), 7.37–7.49 (m, 2H_ar_, *H-*6, *H-*7), 7.70–7.77 (m, 1H_ar_, *H-*5), 8.2–8.26 (m, 1H, *H-*8). ^13^C NMR (126 MHz, CDCl_3_; Figure S19) δ = 46.00 (C*H*_3_), 52.70 (2 aliphatic CH_2_: CH_2_-N-(*C*H_2_-CH_2_)_2_-NCH_3_), 56.08 (2 aliphatic CH_2_: CH_2_-N-(CH_2_-*C*H_2_)_2_-NCH_3_), 61.69 (*C*H_2_-N-(CH_2_-CH_2_)_2_-NCH_3_), 113.61 (C_q,ar_, *C-*2), 118.51 (C-H_ar_, *C-*4), 122.15 (C-H_ar_, *C-*8), 125.00 (C-H_ar_, *C-*6), 125,06 (C_q,ar_, *C-*8*a*), 126.13 (C-H_ar_, *C-*7), 126.72 (C-H_ar_, *C-*5), 127.48 (C-H_ar_, *C-*3), 134.11 (C_q,ar,_*C-*4*a*), 153.57 (C_q,ar_, *C-1*).

##### 3-((4-Methylpiperazin-1-yl)methyl)quinolin-4-ol (**71**)

A mixture of 1-methyl-piperazine (344 μL, 0.310 g, 3.1 mmol), 4-hydroxyquinoline (0.3 g, 2.06 mmol), and 37% formaldehyde (280 μL, 0.230 g, 2.68 mmol) in ethanol (5 mL) was stirred at room temperature for 20 h. Upon removal of the solvent in vacuo, the crude product was crystallized with *n*-hexane (15 mL) and recrystallized with *i*-Pr_2_O (10 mL). Compound **71** was isolated as white crystals (0.29 g, 54%). Mp 164–166 °C. ^1^H NMR (500 MHz, DMSO-*d*_6_; Figure S20) δ = 2.13 (s, 3H, C*H*_3_), 2.35 (d, *J* = 54.9 Hz, 8H), CH_2_-N-(C*H*_2_-C*H*_2_)_2_-NCH_3_, 3.36 (s, 2H, C*H*_2_-N-(CH_2_-CH_2_)_2_-NCH_3_), 7.29 (t, *J* = 7.5 Hz, 1H_ar_, *H-*6), 7.53 (d, *J* = 8.2 Hz, 1H_ar_, *H-*5), 7.61 (t, *J* = 7.6 Hz, 1H_ar_, *H-*7), 7.84 (s, 1H_ar_, *H-*2), 8.1 (d, *J* = 8.0 Hz, 1H_ar_, *H-*8). ^13^C NMR (126 MHz, DMSO-*d*_6_; Figure S21) δ = 45.75 (C*H*_3_), 52.50 (2 aliphatic CH_2_: CH_2_-N-(*C*H_2_-CH_2_)_2_-NCH_3_, 53.55 (*C*H_2_-N-(CH_2_-CH_2_)_2_-NCH_3_)), 54.83 (2 aliphatic CH_2_: CH_2_-N-(CH_2_-*C*H_2_)_2_-NCH_3_), 116.31 (C_q,ar_, *C-*3), 118.25 (C-H_ar_, *C-*5), 122.74 (C-H_ar_, *C-*6), 124.83 (C_q,ar_, *C-*4*a*), 125.06 (C-H_ar_, *C-*8), 131.17 (C-H_ar_, *C-*7), 138.49 (C-H_ar_, *C-*2), 139.7 (C_q,ar_, *C-*8*a*), 176.09 (C_q,ar_, *C-*4).

##### 2-((4-Methylpiperazin-1-yl)methyl)phenol (**72**)

To a solution of 1-methyl-piperazine (554 μL, 0.500 g, 5.00 mmol) in methanol (30 mL) was added 0.4 mL of 5 N HC1 in methanol (1:1) followed by salicylic aldehyde (0.122 g, 1.00 mmol) in 10 mL of methanol. The solution was stirred under nitrogen for 10 min and then solid sodium cyanoborohydride (62.8 mg, 1.00 mmol) was added, and the solution was stirred overnight at room temperature. The mixture was acidified with concentrated hydrochloric acid (pH of about 2), and the methanol was removed under reduced pressure. Water (10 mL) was then added, and the solution was basified with potassium hydroxide and extracted with ether. The ether phase was washed with saturated aqueous sodium chloride, dried (Na_2_SO_4_), and the solvent was evaporated. The residue was isolated as a light yellow oil (0.12 g, 58%). ^1^H NMR (500 MHz, CDCl_3_; Figure S22) δ = δ = 2.31 (s, 3H, C*H*_3_), 2.57 (br s, 8H, CH_2_-N-(C*H*_2_-C*H*_2_)_2_-NCH_3_), 6.78 (t, *J* = 7.4, 1H_ar_, *H-*4), 6.82 (d, *J =* 8.1 Hz, 1H_ar_, *H-*6), 6.98 (d, *J* = 7.0 Hz, 1H_ar_, *H-*3), 7.17 (t, *J* = 8.1, 1H_ar_, *H-*5). ^13^C NMR (126 MHz, CDCl_3_; Figure S23) δ = 46.03 (C*H*_3_), 52.65 (2 aliphatic CH_2_: CH_2_-N-(*C*H_2_-CH_2_)_2_-NCH_3_), 55.09 (2 aliphatic CH_2_: CH_2_-N-(CH_2_-*C*H_2_)_2_-NCH_3_, 61.52 (*C*H_2_-N-(CH_2_-CH_2_)_2_-NCH_3_)), 116.21 (C-H_ar_, *C-*6), 119.28 (C-H_ar_, *C-*4), 121,31 (C_q,ar_, *C-*2), 128.79 (C-H_ar_, *C-*5), 128.96 (C-H_ar_, *C-*3), 157,90 (C_q,ar_,*C-1*).

##### 5-Bromo-7-((3,4-dihydroisoquinolin-2(1*H*)-yl)methyl)quinolin-8-ol (**81**)

A solution of 1,2,3,4-tetrahydroisoquinoline (123 μL, 0.130 g, 0.981 mmol) and 37% formaldehyde (46 μL, 0.036 g, 1.24 mmol) was stirred in EtOH (2 mL) for 1 h. Upon the addition of 5-bromo-8-hydroxyquinoline (0.200 g, 0.892 mmol, in 3 mL EtOH), the reaction mixture was stirred at room temperature for 2 days. The precipitate was filtered and was washed with cold ethanol. Product **81** was isolated as white crystals (0.195 g, 59%). Mp 156–159 °C. ^1^H NMR (500 MHz, CDCl_3_; Figure S24) δ = 2.92 (t, *J* = 5.6 *Hz*, 2H_aliph_, C4′*H*_2_), 2.99 (t, *J* = 5.4 *Hz*, 2H_aliph_, C3′*H*_2_), 3.83 (s, 2H_aliph_, C1′*H*_2_), 4.03 (s, 2H_aliph_, methylene, C*H*_2_), 7.00 (d, *J* = 7.1 *Hz*, 1H_ar_, *H-*5′), 7.20–7.09 (m, 3H_ar_, *H-*6′, *H-*7′, *H-*8′), 7.50 (dd, *J* = 8.5 *Hz*, 4.1 *Hz*, 1H_ar_, *H-*3), 7.67 (s, 1H_ar_, *H-*6), 8.44 (d, *J* = 8.4 *Hz*, 1H_ar_, *H-*4), 8.87 (d, *J* = 3.1 *Hz*, 1H_ar_, *H-*2). ^13^C NMR (126 MHz, CDCl_3_; Figure S25) δ = 27.85 (*C-*4*’*), 49.51 (*C-*3′), 54.75 (*C-1*′), 57.83 (*C*H_2_, methylene), 108.74 (C_q,ar_, *C-*5), 118.44 (C_q,ar_, *C-*4*a*), 121.51 (C-H_ar_, *C-*3), 125.03 (C-H_ar_, *C-*6′), 125.67 (C-H_ar_, *C-*7′), 125.75 (C-H_ar_, *C-*8′), 126.42 (C_q,ar_, *C-*7), 127.83 (C-H_ar_, *C-*5′), 130.34 (C-H_ar_, *C-*6), 132.65 (C_q,ar_, *C-*8*a*′), 132.78 (C_q,ar_, *C-*4*a*′), 134.50 (C-H_ar_, *C-*4), 138.96 (C_q,ar_, *C-*8*a*), 148.32 (C-H_ar_, *C-*2), 151.71 (C_q,ar_, *C-*8). LCMS RT = 4.10 min. ESI^+^*m*/*z*: 370.2 [M + H^+^].

##### 2-((8-Hydroxy-quinolin-7-yl)methyl)-1,2,3,4-tetrahydroisoquinoline-6,7-diol Hydrochloride (**82**)

A solution of 3-hydroxytyramine hydrochloride (0.417 g, 2.2 mmol) and 35% formaldehyde (241 μL, 0.257 g, 3 mmol) in ethanol (3 mL) was stirred for 1 h. Upon the addition of 8-hydroxy-quinoline (0.29 g, 2 mmol) in 3 mL ethanol, the mixture was stirred at room temperature for 24 h. The crude product was dried in vacuo, taken up with dichloromethane, and extracted with 10% NaOH solution (1×), followed by washing with brine and water. The organic phase was dried over Na_2_SO_4_, concentrated under reduced pressure, and washed with ethanol to give the final product **82** as white crystals in a 20% yield (0.25 g). Mp 203–206 °C. ^1^H NMR (500 MHz, DMSO-*d*_6_; Figure S26) δ = 1.58 (s, 2H_aliph_, C4′*H*_2_), 2.01 (br s, 2H_aliph_, C3′*H*_2_), 3.30 (s, 2H_aliph_, C1′*H*_2_), 3.63 (s, 2H_aliph_, methylene C*H*_2_), 5.61 (s, 1H_arom_, *H-*5′), 5.66 (s, 1 H_arom_, *H-*8′), 6.56 (d, *J* = 8.5 *Hz*, 1H_arom_, *H-*5), 6.72 (dd, *J* = 8.2 *Hz*, 4.1 *Hz*, 1H_arom_, *H-*4), 6.90 (d, *J* = 8.5 *Hz*, 1H_arom_, *H-*6), 7.47 (d, *J* = 8.2 *Hz*, 1H_arom_, *H-*3), 8.00 (d, *J* = 2.9 *Hz*, 1H_arom_, *H-*2), 8.15 (br s, 1H, 8-OH). The two OH groups at C-6′ and C7′ are under a broad peak together with DMSO. ^13^C NMR (125 MHz, DMSO-*d*_6_; Figure S27): δ = 24.17 (*C-*4′), 48.53 (*C-*3′), 51.69 (*C-1*′), 52.66 (*C*H_2_, methylene), 112.21 (C-H_ar_, *C-*8′), 113.21 (C-H_ar_, *C-*5′), 115.00 (C-H_ar_, *C-*5), 117.58 (C-H_ar_, *C-*3), 118.44 (C_q,ar_, *C-*7), 121.59 (C_q,ar_, *C-*8*a*′), 122.78 (C_q,ar_, *C-*4*a*′), 129.15 (C_q,ar_, *C-*4*a*), 130.26 (C-H_ar_, *C-*6), 136.22 (C-H_ar_, *C-*4), 138.10 (C_q,ar_, *C-*8*a*), 144.33 (C_q,ar_, *C-*7′), 145.16 (C_q,ar_, *C-*6′), 148.66 (C-H_ar_, *C-*2), 153.20 (C_q,ar_, *C-*8). LCMS RT = 2.46 min, HPLC shown in Figure S9. ESI^+^*m*/*z*: 323.3 [M + H^+^].

##### 7-((Piperidine-1-yl)(pyridin-3-yl)methyl)quinolin-8-ol (**92**)

The mixture of piperidine (408 μL, 0.352 g, 4.13 mmol), 8-hydroxyquinoline (0.4 g, 2.75 mmol), and 3-pyridinecarboxaldehyde (388 μL, 0.442 g, 4.13 mmol) in ethanol (12 mL) was heated at 80 °C for 20 min under microwave conditions. The solvent was removed under reduced pressure, and the residue was crystallized with *n*-hexane (13 mL) and recrystallized with *i*-Pr_2_O (10 mL). Compound **92** was isolated as white crystals (0.572 g, 65%). Mp 178–179 °C. ^1^H NMR (500 MHz, CDCl_3_; Figure S28) δ = 1.50 (s, 2H, *H-*4″), 1.53–1.82 (m, 4H, *H-*3″, *H-*5″), 2.26–2.77 (m, 4H, *H-*6″, *H-*2″), 4.75 (s, 1H, (Ar)_2_-C*H*-N(CH_2_)_5_), 7.19–7.24 (m, 3H_ar_, *H-*3, *H-*5′, *H-*6), 7.35–7.38 (m, 1H_ar_, *H-*5), 7.88 (d, *J* = 7.8Hz, 1H_ar_, *H-*4), 8.04–8.05 (m, 1H_ar_, *H-*4), 8.48–8.49 (m, 1H_ar_, *H-*6′), 8.68 (s, 1H_ar_, *H-*2′), 8.86–8.87 (m, 1H_ar_, *H-*2), 12.02 (br s, 1H, O*H*). ^13^C NMR (126 MHz, CDCl_3_; Figure S29) δ = 24.33 (1 aliphatic CH_2_, *C-*4″), 26.22 (2 aliphatic CH_2_, *C-*3″, *C-*5″, 53.18 (2 aliphatic CH_2_, *C-*6″, *C-*2″)), 71.88 ((Ar)_2_-*C*H-N(CH_2_)_5_), 118.05 (C-H_ar_, *C-*5), 121.68 (C-H_ar_, *C-*3), 122.15 (C_q,ar_, *C-*7), 124.04 (C-H_ar_, *C-*5′), 127.38 (C-H_ar_, *C-*6), 128.33 (C_q,ar_, *C-*4*a*), 135.81 (C-H_ar_, *C-*4), 136.06 (C-H_ar_, *C-*4′), 136.39 (C_q,ar_, *C-*3′), 139.78 (C_q,ar_, *C-*8*a*), 149.08 (C-H_ar_, *C-*6′), 149.43 (C-H_ar_, *C-*2), 149.99 (C-H_ar_, *C-*2′), 152.14 (C_q,ar_, *C-*8). COSY-NMRs are shown in Figures S29 and S30, HSQC NMRs are shown in Figures S31 and S32, and HMBC NMRs are shown in Figures S33 and S34.

### Purchased Compounds

The following compounds were obtained from the indicated vendors.

Previously obtained/resynthesized NSC compounds:^21,46,50^**1**, **2**, **3**, **8**; newly obtained NSC compounds from NCI DTP drug repository: **4**, **5**, **6**, **7**, **9**, **10**, **11**; Asinex (North Carolina and Rijswijk, The Netherlands): **61**, **69**; ChemBridge (California): **60**, **65**, **66**, **73**, **74**; ChemDiv (California): **17**, **18**, **21**, **22**, **23**, **24**, **26**, **27**, **28**, **31**, **32**, **33**, **35**, **36**, **37**, **39**, **40**, **41**, **42**, **59**, **75**, **95**; Enamine Ltd. (Latvia): **38**, **45**, **49**, **58**, **62**, **64**, **67**, **76**, **79**, **84**, **88**, **89**, **90**, **91**, **93**, **96**, **97**, **101**, **102**, **105**, **106**, **107**, **108**, **109**, **110**, **111**, **117**, **120**, **121**; InterBioScreen Ltd. (Russia): **14**; Life Chemicals Europe GmbH. (Munich, Germany): **5**, **87**, **114**; Otava Chemicals Ltd. (Kiew, Ukraine): **19**, **25**, **29**, **30**, **43**, **44**, **46**, **47**, **48**, **50**, **52**, **54**, **55**, **56**, **57**, **103**, **119**; Sigma-Aldrich (Hungary): **12**, **13**; and UkrOrgSyntez Ltd. (Ukraine): **53**, **63**, **68**, **77**, **78**, **80**, **85**, **86**, **94**, **98**, **99**, **100**, **104**, **112**, **113**, **115**, **116**, **118**.

### UV–Visible Spectrophotometric Titrations

Spectrophotometrical determination of p*K*_a_ values was performed as previously reported.^20,50^ An Agilent Cary 8454 diode array spectrophotometer was used to record the UV–visible spectra in the interval 200–800 nm. The path length was between 1 and 5 cm. The spectrophotometric titrations were performed in water with 0.2% (v/v) DMSO on samples containing the compounds at 2–50 μM in the pH range from 2 to 11.5 at 25.0 ± 0.1 °C at an ionic strength of 0.10 M (KCl). Proton dissociation constants and the individual spectra of the species in the different protonation states were calculated with the computer program PSEQUAD.^62^

### Pan-Assay Interference Compounds (PAINS)

As chelators and Mannich bases, compounds described here fall into the category of pan-assay interfering compounds (PAINs), which have been reported to be problematic in a wide range of target-based assays, covering ion channels, enzymes, and protein–protein interactions due to their reactivity, spectroscopic properties, and the ability to form metal complexes as well as aggregates.^63,64^ Redox-active compounds might interfere with proteins, and by inactivating the target, they lead to false-positive results.^64^ Still in the areas of oncology, microbiology, and parasitology, reactive, photosensitive, and redox-active compounds may be particularly suited for therapeutic uses.^63^ Often, in these areas, the exact target of chelators is not known, and therefore the phenotypic drug discovery strategy is applied, where little assumptions are made concerning the participation of specific molecular targets and/or signaling pathways. Instead, compounds are investigated in complex biological systems and compound-induced physiological responses or phenotypes are monitored in cells, tissues, or whole organisms.^65,66^ The induction of cell death upon treatment with a certain compound can be seen as a phenotypic effect.^66^ To exclude artifacts related to PAINs, the results were confirmed by an independent cell line pair and also using an independent assay using cells expressing the fluorescent mCherry protein.^67^ As apparent from Figure S4, the assays give comparable results.

### Cell Lines

The human uterine sarcoma cell lines MES-SA and the doxorubicin-selected MES-SA/Dx5 were obtained from ATCC (MES-SA: No. CRL-1976, MES-SA/Dx5: no. CRL-1977) and cultivated in Dulbecco’s modified Eagle’s medium (DMEM, Sigma-Aldrich, Hungary) supplemented with 10% fetal bovine serum, 5 mmol/L glutamine, and 50 unit/mL penicillin and streptomycin (Life Technologies, Hungary).^51,68^ A431-ABCB1 cells were engineered by retroviral transduction, as described in.^46^ A431 cells were maintained in DMEM (Life Technologies) supplemented as above.

### PrestoBlue Viability Assay

Cell viability was determined by the resazurin-based PrestoBlue assay according to the manufacturer’s instructions.^54,69^ Briefly, cells were seeded into 96-well tissue culture plates in a density of 5000 cells per well and allowed to attach for 24 h before serial dilutions of the test compounds were added. After 72 h of incubation with the test compounds, supernatants were removed, and a 5% solution of the PrestoBlue reagent (Thermo Fisher Scientific) was added to each well. Emission was detected by a PerkinElmer EnSpire multimode plate reader at 585 nm (excitation at 555 nm) after 1 h incubation at 37 °C.

### Viability Assay Using mCherry-Transfected MES-SA and MES-SA/Dx5 Cells^67^

Cells were seeded either on 96- or 384-well plates (Greiner bio-one, Hungary), using a volume of 100 or 40 μL and a density of 5000 or 2500 cells per well, respectively, and allowed to attach for 24 h. Dilutions of the test compounds were added to achieve the required final concentration in a final volume of 200 μL per well for 96- and 60 μL for 384-well plates. After a 72 h incubation period, fluorescence was measured using a PerkinElmer EnSpire Multimode Plate Reader at 585 nm excitation and 610 nm emission wavelengths.

## Supporting Information Available

Test results of 8-OHQ derivatives obtained from the NCI DTP drug repository in further cancer cell lines (Table S1); IC_50_ values for compounds from Figure 1 (Table S2); IC_50_ values for compounds from Figure 3 (Table S3); measured and computed proton dissociation constants (p*K*a) (Table S4); effect of aromatic aldehyde moieties in comparison to formaldehyde-derived Mannich bases with tertiary amines (extended version of Figure 7) (Table S5); representative UV–visible spectra and molar absorbance spectra of species for compounds **38** and **9** (Figure S1); three-dimensional (3D) alignment of ligands (Figure S2); comparing the toxicity (A) and selectivity (B) of MMPs bearing an aromatic ring at the methylene carbon with and without a chloro-substituent in R5 (Figure S3); correlation of toxicity and p*K*a values with color coding scheme representing structural modifications (Figure S4); impact of the calculated chemical properties on toxicity (Figure S5); comparing the effect of 80 investigated chelators obtained in different assays in MES-SA (A, C) and MES-SA/Dx5 (B, D) cells (Figure S6); exemplary HPLC traces of compounds **34**, **16**, and **82** (Figures S7–S9); NMR spectra (^1^H,^13^C) (Figures S10–S29); structure of compound 92 with enumeration (Figure S30); and representative 2D NMRs for compound **92** (COSY, HSQC, HMBC) (Figures S31–S36) (PDF)
Molecular Formula Strings (spreadsheet with biodata.csv) (CSV)

## Abbreviations Used

8-OHQ: 8-hydroxyquinoline (8-OHQ)
ABC: ATP-binding cassette (ABC)
DTP: Developmental Therapeutics Program of National Cancer institute
MDR: multidrug resistance
MMP: matched molecular pair
P-gp: P-glycoprotein (ABCB1)
SARM: structure–activity matrix
SR: selectivity ratio
TQ: tariquidar
