# Supplementary material for: Structure–Activity Relationships of 8-Hydroxyquinoline-Derived Mannich Bases with Tertiary Amines Targeting Multidrug-Resistant Cancer
Source: J Med Chem. 2022 May 25;65(11):7729–45. doi: 10.1021/acs.jmedchem.2c00076 (PMC9189845; doi:10.1021/acs.jmedchem.2c00076)
Supplement: Supplementary file 1 — jm2c00076_si_001.pdf [file jm2c00076_si_001.pdf]

Supporting information

Structure activity relationships of 8-hydroxyquinoline derived Mannich bases with tertiary amines targeting multidrug resistant cancer

Veronika F.S. Pape[a,b*], Roberta Palkó[c], Szilárd Tóth[a], Miklós J. Szabó[d], Judit Sessler[a], György Dormán[e], Éva A. Enyedy[f], Tibor Soós[c], István Szatmári[g], Gergely Szakács[a,h*]

[a] Institute of Enzymology, Research Centre for Natural Sciences, Eötvös Loránd Research Network, Magyar Tudósok körútja 2, H-1117 Budapest, Hungary

[b] Department of Physiology, Semmelweis University, Faculty of Medicine, Tűzoltó utca 37-47, H-1094 Budapest, Hungary

[c] Institute of Organic Chemistry, Research Centre for Natural Sciences, Eötvös Loránd Research Network, Magyar Tudósok körútja 2, H-1117 Budapest, Hungary

[d] ChemAxon Ltd., Váci út 133, H-1138 Budapest, Hungary

[e] TargetEx Ltd., Madách Imre u 31/2., H-2120 Dunakeszi, Hungary

[f] MTA-SZTE Lendület Functional Metal Complexes Research Group, Department of Inorganic and Analytical Chemistry, University of Szeged, Dóm tér 7, H-6720 Szeged, Hungary

[g] Institute of Pharmaceutical Chemistry and Stereochemistry Research Group of Hungarian Academy of Sciences, University of Szeged, Eötvös u. 6, H-6720 Szeged, Hungary

[h] Institute of Cancer Research, Medical University of Vienna, Borschkegasse 8a, A-1090 Vienna, Austria

To whom correspondence should be addressed:

* Gergely Szakács: gergely.szakacs@meduniwien.ac.at

* Veronika F.S. Pape: veronika.pape@med.semmelweis-univ.hu

Contents

Supplementary Tables

Table S1: Test results of 8OHQ derivatives obtained from the NCI DTP drug repository in further cancer cell lines ____ S3

Table S2: IC_{50} values for compounds from Figure 1 ____ S4

Table S3: IC_{50} values for compounds from Figure 3 ____ S6

Table S4: measured and computed proton dissociation constants (pK_a) ____ S8

Table S5: Effect of aromatic aldehyde moieties in comparison to formaldehyde derived Mannich bases with tertiary amines (extended version of Figure 7) ____ S14

Supplementary discussion

Analysis of computed vs experimentally determined pK_a values ____ S11

Supplementary Figures

Figure S1: Representative UV-visible spectra and molar absorbance spectra of species for compounds **38** and **9** S7

Figure S2: 3D alignment of ligands S12

Figure S3: comparing the toxicity (A) and selectivity (B) of MMPs bearing an aromatic ring at the methylene carbon with and without chloro-substituent in R5 S13

Figure S4: Correlation of toxicity and pK_a values with color coding scheme representing structural modificaitons S13

Figure S5: Impact of the calculated chemical properties on toxicity S15

Figure S6: Comparing the effect of 80 investigated chelators obtained in different assays in MES-SA (A, C) and MES-SA/Dx5 (B, D) cells S16

Figures S7-S9: Exemplary HPLC traces of compounds **34**, **16** and **82** S17

Figures S10-S29: NMR spectra (^{1}H, ^{13}C) S18

Figure S30: structure of compound **92** with enumeration S28

Figures S31-S36: representative 2D NMRs for compound **92** (COSY, HSQC, HMBC) S28

Table S1: Test results of 8OHQ derivatives obtained from the NCI DTP drug repository in further cancer cell lines

			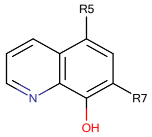				
	**NSC**	**R5**	**R7**		**IC_{50} / µM A431**	**IC_{50} / µM A431/B1**	**SR**
**1**	693872	H	-diethylamino-methyl	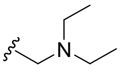	14.84 ± 0.41	4.51 ± 0.73	3.29
**2**	693871	H	-pyrrolidin-1-yl-methyl	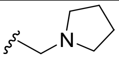	18.19 ± 1.02	6.60 ± 1.10	2.75
**3**	57969	H	-piperidin-1-ylmethyl	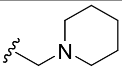	7.30 ± 0.98	1.62 ± 0.92	4.52
**4**	92559	H	-[bis(2-chloroethyl)-amino]methyl	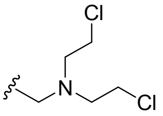	106.69 ± 0.48	> 200	
**5**		Cl	-diethylamino-methyl	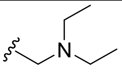	9.29 ± 1.55	1.58 ± 1.11	5.89
**6**	130803	Cl	-[bis(2-hydroxyethyl)-amino]methyl	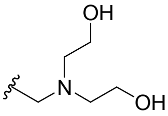	73.63 ± 0.75	20.47 ± 0.95	3.60
**7**	376461	NO_2	-[bis(2-hydroxyethyl)-amino]methyl	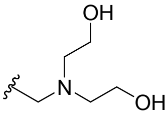	9.79 ± 1.13	9.18 ± 0.58	1.07
**8**	662298	H	-morpholin-1-ylmethyl	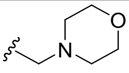	14.49 ± 0.32	6.51 ± 0.65	2.23
**9**	130821	Cl	-methyl-piperazin-1-ylmethyl	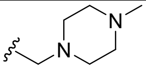	16.99 ± 1.04	3.55 ± 0.50	4.79
**10**	20514	$-CH_2O(CH_2)_3CH_3$	-methyl-piperazin-1-ylmethyl	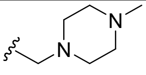	11.25 ± 0.37	4.57 ± 0.20	2.46
**11**	130807	$-NCOCH_3$	-piperidin-1-ylmethyl	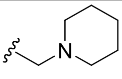	> 100	58.21 ± 0.65	

Table S2: IC_{50} values for compounds from Figure 1 (SARM for R5R7 substitution). Data represent mean values with standard deviation obtained from 2 to 53 independent PrestoBlue assays for MES-SA and MES-SA/Dx5 cells in absence and presence (values in brackets) of 1µM of the P-gp inhibitor TQ; and for A431 and A431-B1 cells. MDR-selectivity ratio (SR) is defined as the fraction of IC_{50} values obtained in P-gp negative vs. positive cells.

**ENZ-Code**	**R5**	**R7**	**IC_{50} / µM MES-SA**	**IC_{50} / µM MES-SA/Dx5**	**SR (MES-SA / MES-SA/Dx5)**	**IC_{50} / µM A431**	**IC_{50} / µM A431-B1**	**SR (A431 / A431-B1)**
**12**	H	H	5.04 ± 0.51 (5.13 ± 0.05)	3.04 ± 0.35 (2.20 ± 0.18)	1.66 (2.34)			
**13**	Cl	H	8.26 ± 0.10	5.15 ± 0.48	1.60			
**14**	$-CH_2OCH_3$	H	2.51 ± 0.13	1.79 ± 0.09	1.40			
**2**	H	-pyrrolidin-1-yl-methyl	4.64 ± 0.82 (3.98 ± 0.17)	1.47 ± 0.25 (2.82 ± 0.19)	3.16 (1.41)	17.12 ± 4.65	3.24 ± 0.80	5.28
**15**	Cl	-pyrrolidin-1-yl-methyl	3.46 ± 0.51 (4.34 ± 0.45)	0.30 ± 0.06 (3.21 ± 0.44)	11.53 (1.35)	7.45 ± 0.15	1.97 ± 0.25	3.79
**16**	Br	-pyrrolidin-1-yl-methyl	3.90 ± 0.87 (4.58 ± 1.05)	0.41 ± 0.14 (3.14 ± 0.89)	9.45 (1.46)	4.32 ± 0.39	1.54 ± 0.51	2.81
**17**	$-CH_2OCH_3$	-pyrrolidin-1-yl-methyl	11.10 ± 1.78 (10.47 ± 0.17)	1.37 ± 0.26 (3.59 ± 0.53)	8.09 (2.92)	53.24 ± 6.49	4.05 ± 0.07	13.16
**18**	$-CH_2OCH_2CH_3$	-pyrrolidin-1-yl-methyl	8.61 ± 1.31 (8.73 ± 0.54)	0.93 ± 0.18 (5.25 ± 0.18)	9.27 (1.6)	35.24 ± 11.52	5.92 ± 2.14	5.95
**3**	H	-piperidin-1-yl-methyl	5.21 ± 0.95 (4.18 ± 0.97)	0.85 ± 0.18 (2.69 ± 0.82)	6.13 (1.56)	10.13 ± 2.95	2.48 ± 0.70	4.09
**19**	Cl	-piperidin-1-yl-methyl	2.49 ± 0.39 (3.38 ± 0.36)	0.29 ± 0.05 (2.58 ± 0.39)	8.71 (1.31)	6.67 ± 1.69	1.69 ± 0.27	3.96
**20**	Br	-piperidin-1-yl-methyl	1.79 ± 0.16 (1.71 ± 0.16)	0.17 ± 0.02 (1.32 ± 0.08)	10.67 (1.29)	6.91 ± 1.37	2.16 ± 0.35	3.20
**21**	$-CH_2OCH_3$	-piperidin-1-yl-methyl	9.35 ± 0.61 (9.56 ± 0.39)	0.92 ± 0.11 (7.59 ± 0.08)	10.19 (1.26)	22.58 ± 4.32	3.53 ± 0.14	6.39
**22**	$-CH_2OCH_2CH_3$	-piperidin-1-yl-methyl	8.77 ± 1.07 (9.13 ± 0.32)	0.79 ± 0.14 (7.08 ± 0.23)	11.12 (1.29)	25.15 ± 8.88	4.17 ± 1.49	6.03
**23**	$-CH_2O(CH_2)_2CH_3$	-piperidin-1-yl-methyl	3.72 ± 0.25 (3.55 ± 0.08)	0.34 ± 0.02 (2.40 ± 0.04)	10.97 (1.48)	6.72 ± 0.57	1.53 ± 0.55	4.38
**24**	$-CH_2OCH(CH_3)_2$	-piperidin-1-yl-methyl	6.03 ± 0.29 (5.76 ± 0.15)	0.48 ± 0.04 (4.37 ± 0.24)	12.57 (1.32)	7.53 ± 1.00	1.91 ± 0.18	3.94
**25**	H	-4-Methylpiperidin-1-yl-methyl	2.70 ± 0.21 (2.88 ± 0.06)	0.26 ± 0.04 (1.59 ± 0.10)	10.37 (1.82)	8.93 ± 1.44	1.46 ± 0.08	6.12
**26**	$-CH_2OCH_3$	-4-Methylpiperidin-1-yl-methyl	6.20 ± 0.66 (6.47 ± 0.41)	0.58 ± 0.05 (4.68 ± 0.16)	10.73 (1.38)	14.45 ± 3.84	2.29 ± 0.28	6.30
**27**	$-CH_2OCH_2CH_3$	-4-Methylpiperidin-1-yl-methyl	4.09 ± 0.42 (4.48 ± 0.37)	0.47 ± 0.05 (3.05 ± 0.47)	8.71 (1.47)	11.79 ± 4.34	2.37 ± 0.87	4.97
**28**	$-CH_2OCH(CH_3)_2$	-4-Methylpiperidin-1-yl-methyl	2.84 ± 0.35 (3.02 ± 0.09)	0.36 ± 0.04 (2.15 ± 0.22)	7.96 (1.41)	7.24 ± 1.78	1.72 ± 0.15	4.21
**8**	H	-morpholin-1-yl-methyl	7.55 ± 0.91	4.33 ± 0.31	1.75			
**29**	Cl	-morpholin-1-yl-methyl	1.52 ± 0.07 (1.66 ± 0.02)	0.82 ± 0.06 (1.17 ± 0.01)	1.86 (1.41)	6.60 ± 3.57	2.42 ± 0.29	2.73
**30**	Br	-morpholin-1-yl-methyl	1.13 ± 0.13 (1.32 ± 0.01)	0.46 ± 0.04 (0.85 ± 0.06)	2.46 (1.54)	9.30 ± 3.05	2.89 ± 0.25	3.22
**31**	$-CH_2OCH_3$	-morpholin-1-yl-methyl	9.89 ± 1.52 (8.97 ± 1.01)	5.64 ± 0.45 (7.77 ± 0.31)	1.75 (1.15)	34.41 ± 8.42	12.08 ± 1.47	2.85
**32**	$-CH_2OCH_2CH_3$	-morpholin-1-yl-methyl	5.85 ± 0.54	3.00 ± 0.30	1.95	24.70 ± 10.90	5.58 ± 0.58	4.43
**33**	$-CH_2OCH(CH_3)_2$	-morpholin-1-yl-methyl	3.12 ± 0.60	1.74 ± 0.29	1.79	13.23 ± 6.23	4.76 ± 0.23	2.78
**34**	H	-methyl-piperazin-1-yl-methyl	16.70 ± 1.86 (15.49 ± 0.19)	8.67 ± 1.69 (14.13 ± 0.00)	1.93 (1.10)	25.54 ± 1.98	11.34 ± 0.95	2.25
**9**	Cl	-methyl-piperazin-1-yl-methyl	5.39 ± 0.47 (5.14 ± 0.27)	1.90 ± 0.37 (4.30 ± 0.57)	2.84 (1.19)	15.92 ± 7.44	6.67 ± 2.40	2.38

**35**	$-CH_2OCH_3$	-methyl-piperazin-1-yl-methyl	24.77 ± 3.36 (30.21 ± 0.64)	8.82 ± 1.41 (27.54 ± 0.13)	2.81 (1.10)	77.23 ± 11.55	26.49 ± 0.12	2.92
**36**	$-CH_2OCH_2CH_3$	-methyl-piperazin-1-yl-methyl	22.96 ± 1.46 (22.93 ± 1.08)	7.43 ± 0.46 (17.12 ± 2.19)	3.09 (1.34)	66.56 ± 20.78	24.11 ± 10.10	2.76
**37**	$-CH_2O(CH_2)_2CH_3$	-methyl-piperazin-1-yl-methyl	13.52 ± 0.86 (12.31 ± 0.28)	3.24 ± 0.19 (7.41 ± 0.01)	4.17 (1.66)	19.85 ± 9.14	6.19 ± 1.73	3.21
**10**	$-CH_2O(CH_2)_3CH_3$	-methyl-piperazin-1-yl-methyl	7.23 ± 0.14 (5.00 ± 0.08)	2.12 ± 0.15 (3.92 ± 0.91)	3.40 (1.28)	11.15 ± 0.83	4.57 ± 0.17	2.44
**38**	Cl	-phenyl-piperazin-1-yl-methyl	3.42 ± 1.17 (3.57 ± 1.18)	0.59 ± 0.26 (1.87 ± 0.75)	5.84 (1.91)	7.38 ± 1.23	6.31 ± 2.64	1.17
**39**	$-CH_2OCH_3$	-phenyl-piperazin-1-yl-methyl	1.08 ± 0.15 (1.20 ± 0.05)	0.48 ± 0.07 (0.85 ± 0.08)	2.23 (1.41)	9.99 ± 4.76	2.33 ± 0.35	4.29
**40**	$-CH_2OCH_2CH_3$	-phenyl-piperazin-1-yl-methyl	1.27 ± 0.15 (1.62 ± 0.005)	0.34 ± 0.04 (1.05 ± 0.10)	3.72 (1.54)	6.24 ± 1.95	1.94 ± 0.19	3.22
**41**	$-CH_2O(CH_2)_2CH_3$	-phenyl-piperazin-1-yl-methyl	1.45 ± 0.13 (1.45 ± 0.07)	0.24 ± 0.03 (0.83 ± 0.01)	6.01 (1.74)	5.69 ± 0.76	2.41 ± 0.37	2.36
**42**	$-CH_2OCH(CH_3)_2$	-phenyl-piperazin-1-yl-methyl	1.06 ± 0.19 (1.01 ± 0.16)	0.25 ± 0.05 (0.66 ± 0.13)	4.24 (1.54)	7.35 ± 2.49	1.84 ± 0.48	4.00
**43**	Cl	-2-pyridin-piperazin-1-yl-methyl	2.19 ± 0.12 (2.19 ± 0.17)	0.33 ± 0.05 (1.23 ± 0.05)	6.55 (1.78)	9.41 ± 1.41	8.06 ± 2.89	1.17
**44**	Cl	-4-fluorophenyl-piperazin-1-yl-methyl	1.61 ± 0.30 (1.75 ± 0.16)	0.25 ± 0.01 (0.65 ± 0.05)	6.56 (2.70)	4.04 ± 0.39	3.25 ± 0.94	1.24

Table S3: IC_{50} values for compounds from Figure 3 (SARM for shift from R7 to R5). Data represent mean values with standard deviation obtained from 2 to 53 independent PrestoBlue assays for MES-SA and MES-SA/Dx5 cells in absence and presence (values in brackets) of 1µM of the P-gp inhibitor TQ; and for A431 and A431-B1 cells. MDR-selectivity ratio (SR) is defined as the fraction of IC_{50} values obtained in P-gp negative vs. positive cells.

**comp**	**R5**	**R7**	**IC_{50} / µM MES-SA**	**IC_{50} / µM MES-SA/Dx5**	**SR (MES-SA / MES-SA/Dx5)**	**IC_{50} / µM A431**	**IC_{50} / µM A431-B1**	**SR (A431 / A431-B1)**
**45**	-diethylamino-methyl	H	4.50 ± 0.04	5.02 ± 0.26	0.90			
**1**	H	-diethylamino-methyl	7.35 ± 1.28 (5.15 ± 0.48)	1.64 ± 0.24 (3.04 ± 0.38)	4.49 (1.69)	13.37 ± 0.18	4.99 ± 0.52	2.68
**5**	Cl	-diethylamino-methyl	2.97 ± 0.33 (3.25 ± 0.31)	0.35 ± 0.06 (1.82 ± 0.13)	8.44 (1.78)	10.03 ± 4.20	1.52 ± 0.32	6.60
**46**	-pyrrolidin-1-yl-methyl	H	7.81 ± 0.50	5.31 ± 0.52	1.47			
**2**	H	-pyrrolidin-1-yl-methyl	4.64 ± 0.82 (3.98 ± 0.17)	1.47 ± 0.25 (2.82 ± 0.19)	3.16 (1.41)	17.12 ± 4.65	3.24 ± 0.80	5.28
**15**	Cl	-pyrrolidin-1-yl-methyl	3.46 ± 0.51 (4.34 ± 0.45)	0.30 ± 0.06 (3.21 ± 0.44)	11.53 (1.35)	7.45 ± 0.15	1.97 ± 0.25	3.79
**47**	-piperidin-1-ylmethyl	H	7.45 ± 0.06	4.36 ± 0.40	1.71			
**3**	H	-piperidin-1-ylmethyl	5.21 ± 0.95 (4.18 ± 0.97)	0.85 ± 0.18 (2.69 ± 0.82)	6.13 (1.56)	10.13 ± 2.95	2.48 ± 0.70	4.09
**19**	Cl	-piperidin-1-ylmethyl	2.49 ± 0.39 (3.38 ± 0.36)	0.29 ± 0.05 (2.58 ± 0.39)	8.71 (1.31)	6.67 ± 1.69	1.69 ± 0.27	3.96
**48**	-4-[ethoxy(oxo)methane]-piperidin-1-yl-methyl	H	4.62 ± 1.03	3.54 ± 0.72	1.31			
**49**	Cl	-4-[ethoxy(oxo)methane]-piperidin-1-yl-methyl	1.78 ± 0.12 (1.70 ± 0.07)	0.18 ± 0.03 (0.98 ± 0.09)	9.91 (1.73)	8.57 ± 2.94	1.99 ± 0.25	4.30
**50**	-3,4-benzo-piperidin-1-ylmethyl	H	16.74 ± 1.50	16.99 ± 0.49	0.99			
**51**	H	-3,4-benzo-piperidin-1-ylmethyl	3.16 ± 0.41 (2.97 ± 0.31)	1.04 ± 0.10 (2.16 ± 0.31)	3.03 (1.37)	4.74 ± 0.04	2.39 ± 0.02	1.99
**52**	-azepan-1-ylmethyl	H	7.15 ± 0.27	5.84 ± 0.69	1.23			
**53**	Cl	-azepan-1-ylmethyl	2.76 ± 0.14 (2.64 ± 0.27)	0.33 ± 0.06 (2.00 ± 0.19)	8.40 (1.32)	5.57 ± 1.08	2.94 ± 1.69	1.90
**54**	-morpholin-1-ylmethyl	H	6.99 ± 0.31	9.58 ± 0.33	0.73			
**8**	H	-morpholin-1-ylmethyl	7.55 ± 0.91	4.33 ± 0.31	1.75			
**29**	Cl	-morpholin-1-ylmethyl	1.52 ± 0.07 (1.66 ± 0.02)	0.82 ± 0.06 (1.17 ± 0.01)	1.86 (1.41)	6.60 ± 3.57	2.42 ± 0.29	2.73
**34**	H	-methyl-piperazin-1-ylmethyl	16.70 ± 1.86 (15.49 ± 0.19)	8.67 ± 1.69 (14.13 ± 0.00)	1.93 (1.10)	25.54 ± 1.98	11.34 ± 0.95	2.25
**9**	Cl	-methyl-piperazin-1-ylmethyl	5.39 ± 0.47 (5.14 ± 0.27)	1.90 ± 0.37 (4.30 ± 0.57)	2.84 (1.19)	15.92 ± 7.44	6.67 ± 2.40	2.38
**55**	-ethyl-piperazin-1-ylmethyl	H	17.69 ± 3.14	21.38 ± 0.10	0.83			
**56**	Cl	-ethyl-piperazin-1-ylmethyl	3.33 ± 0.38 (3.32 ± 0.22)	1.18 ± 0.11 (2.46 ± 0.15)	2.82 (1.35)	8.65 ± 0.11	5.14 ± 0.00	1.68
**57**	-phenyl-piperazin-1-ylmethyl	H	3.67 ± 0.35	4.19 ± 0.15	0.88			

**38**	Cl	-phenyl-piperazin-1-ylmethyl	3.42 ± 1.17 (3.57 ± 1.18)	0.59 ± 0.26 (1.87 ± 0.75)	5.84 (1.91)	7.38 ± 1.23	6.31 ± 2.64	1.17
**58**	-2-methylphenyl-piperazin-1-ylmethyl	H	5.84 ± 1.02 (5.78 ± 0.49)	3.01 ± 0.58 (3.02 ± 0.01)	1.94 (1.91)	23.33 ± 0.00	31.19 ± 0.00	0.75

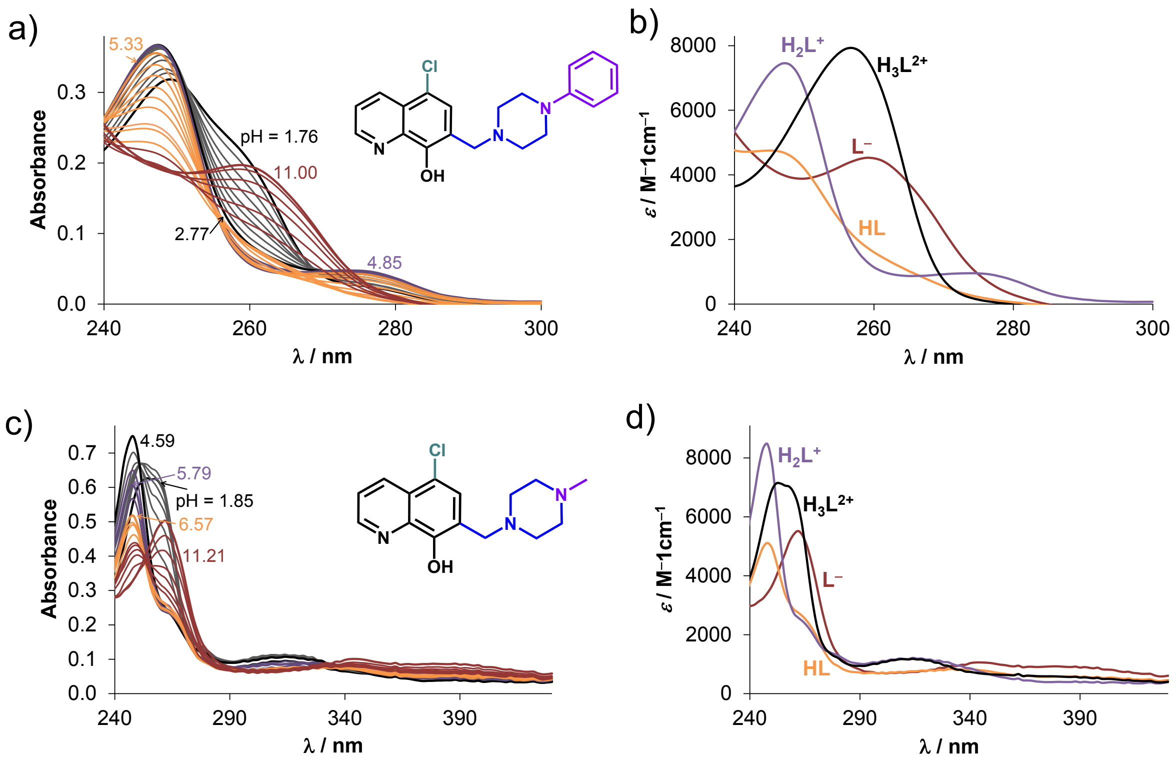

Figure S1: Representative UV-visible spectra recorded at various pH values for compound (a) **38** and (b) **9**, and the molar absorbance spectra of their species in the different protonation state for (c) **38** and (d) **9**. HL denotes the neutral form of the compound. ($c_{[38]}$ = 25 µM, l = 2 cm; $c_{[9]}$ = 19 µM, l = 5 cm, ionic strength: 0.1 M (KCl); T = 25 °C). On the basis of the spectral changes, the pK_a values (Table S4) were calculated using the program PSEQUAD [L. Zékány and I. Nagypál, in Computational Methods for the Determination of Stability Constants, ed. D. L. Leggett, Plenum Press, New York, 1985, pp. 291–353.] in addition to the individual molar absorbance spectra.

Table S4: Proton dissociation constants (pK_a) of compounds displayed in Figure 6, as computed by the ChemAxon software and determined experimentally for the 8-hydroxyquinoline derivatives in water {T = 25°C}. Standard error is given in cases, for which data were determined in this study.

**N°**	**Structure**	Computed			Experimentally determined			**Remark**
		**pK_a (OH)**	**pK_a ($N_{quin}H^+$)[a]**	**pK_a (other)**	**pK_a (OH)**	**pK_a ($N_{quin}H^+$)[a]**	**pK_a (other)**	
**12**	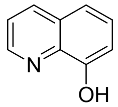	9.36	4.83	–	9.51 [b]	4.99 [b]	–	core structure
**13**	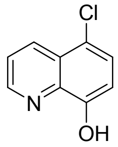	8.37	4.01	–	7.6 [d]	3.8 [d]	–	5-chlorinated core structure
**3**	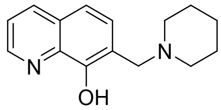	7.45	3.18	10.12	6.99 [b]	2.69 [b]	> 11 [b] ($N_{piperidinium}H^+$)	
**19**	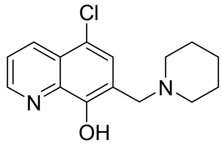	7.22	2.44	8.98	5.80±0.03	< 2	> 11 ($N_{piperidinium}H^+$)	halogen in R5 (chlorine)
**20**	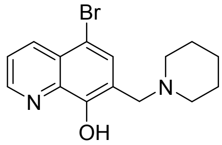	7.23	2.54	8.61	7.16±0.03	1.8±0.1	> 11 ($N_{piperidinium}H^+$)	halogen in R5 (bromine)
**8**	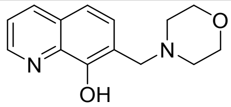	8.23	3.19	7.15	10.37 [a]	2.59 [a]	6.25 [a] ($N_{morpholinium}H^+$)	Introduction of additional heteroatom in R7 ring
**29**	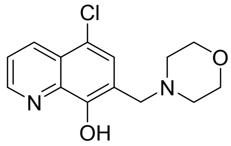	7.66	2.44	6.38	9.68 [c]	< 2 [c]	5.83 [c] ($N_{morpholinium}H^+$)	Introduction of additional heteroatom and halogen in R5
**9**	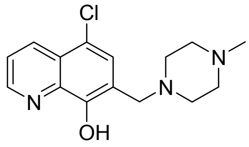	8.08	2.73	$N_{piperazinium}H^+$ 1.36 (closer to quinoline ring) / 7.21 (at methyl)	9.63±0.03	3.56±0.03	5.61±0.03 ($N_{piperazinium}H^+$ at methyl)	Introduction of additional heteroatom and halogen in R5
**38**	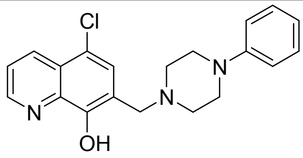	7.13	2.47	$N_{piperazinium}H^+$ 8.23 (closer to quinoline ring) / -0.62 (at phenyl)	6.21±0.03	1.7±0.1	10.10±0.03 ($N_{piperazinium}H^+$ closer to quinolone ring)	Introduction of additional heteroatom plus aromatic ring and halogen in R5

**14**	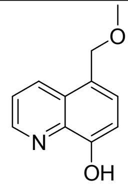	8.81	4.43	–	9.43±0.03	4.64±0.03	–	Alkoxyalkyl in R5
**18**	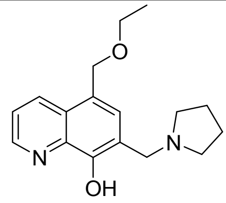	7.66	2.83	9.71	7.89±0.03	2.49±0.03	11.21±0.03 ($N_{pyrrolidinium}H^{+}$)	Alkoxyalkyl in R5
**24**	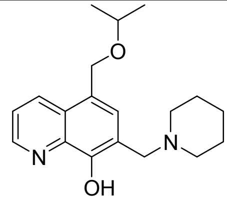	7.42	2.83	9.83	7.66±0.03	2.43±0.03	> 11 ($N_{piperidinium}H^{+}$)	Alkoxyalkyl in R5
**47**	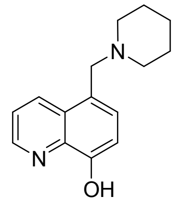	8.33	3.14	9.52	8.45±0.03	3.83±0.03	10.23±0.03 ($N_{piperidinium}H^{+}$)	R7 to R5
**55**	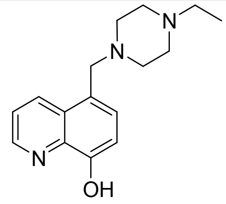	8.93	3.56	$N_{piperazinium}H^{+}$: 1.75 (closer to quinoline ring) / 8.12 (N-ethyl)	9.67±0.03	4.94±0.03	$N_{piperazinium}H^{+}$: 2.60±0.03 (closer to quinoline ring) / 5.48±0.03 (N-ethyl)	R7 to R5
**57**	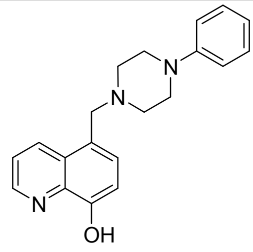	8.90	3.20	$N_{piperazinium}H^{+}$: 7.97 (closer to quinolone ring) / -0.69 (N-phenyl)	8.88±0.03	3.63±0.03	$N_{piperazinium}H^{+}$: 6.69±0.03 (closer to quinolone ring)	R7 to R5
**51**	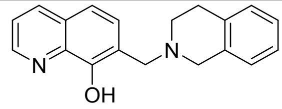	7.70	3.20	9.16	6.24±0.03	3.07±0.03	10.68±0.03 ($N_{piperidinium}H^{+}$)	Introduction of annulated aromatic ring in R7
**79**	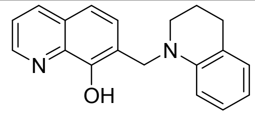	8.60	4.50	2.85	9.98±0.03	4.45±0.03	1.7±0.1 ($N_{piperidinium}H^{+}$)	Introduction of annulated aromatic ring in R7

**97**	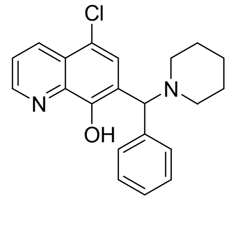	6.95	2.42	9.50	6.46±0.03	1.7±0.1	10.75±0.03 ($N_{piperidinium}H^+$)	Introduction of additional aromatic ring at methylene bridge
**108**	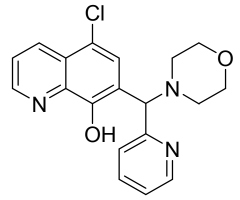	7.55	2.67	5.97 ($N_{morpholinium}H^+$) / 2.06 ($N_{pyridinium}H^+$)	9.42±0.03	1.89±0.03	5.53±0.03 ($N_{morpholinium}H^+$) / < 2 ($N_{pyridinium}H^+$)	Introduction of additional aromatic ring at methylene bridge
**De-Cl-Q4**	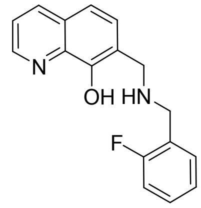	7.63	2.68	8.77 ($N_{benzylamine}H_2^+$)	6.42 [c]	3.21 [c]	10.69 [c] ($N_{benzylamine}H_2^+$)	Previously reported (with secondary amine in R7)
**Q4**	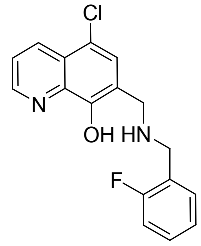	7.19	1.88	8.58 ($N_{benzylamine}H_2^+$)	5.16 [b]	< 2 [b]	8.54 [b] ($N_{benzylamine}H_2^+$)	Previously reported (with secondary amine in R7) with halogen in R5 (chlorine)

[a] quin: quinolinium

[b] Data taken from: Pape, V. F. S.; May, N. V.; Gál, G. T.; Szatmári, I.; Szeri, F.; Fülöp, F.; Szakács, G.; Enyedy, É. A. Impact of Copper and Iron Binding Properties on the Anticancer Activity of 8-Hydroxyquinoline Derived Mannich Bases. *Dalton Trans.* **2018**, *47* (47), 17032–17045. https://doi.org/10.1039/C8DT03088J.

[c] Data taken from: Pape, V. F. S.; Gaál, A.; Szatmári, I.; Kucsma, N.; Szoboszlai, N.; Streli, C.; Fülöp, F.; Enyedy, É. A.; Szakács, G. Relation of Metal-Binding Property and Selective Toxicity of 8-Hydroxyquinoline Derived Mannich Bases Targeting Multidrug Resistant Cancer Cells. *Cancers* **2021**, *13* (1), 154. https://doi.org/10.3390/cancers13010154.

[d] Data taken from: Mészáros, J. P.; Poljarević, J.; Szatmári, I.; Csuvik, O.; Fulop, F.; Szoboszlai, N.; Spengler, G.; Enyedy, E. A. An 8-Hydroxyquinoline-Proline Hybrid with Multidrug Resistance Reversal Activity and Solution Chemistry of Its Half-Sandwich Organometallic Ru and Rh Complexes. *Dalton Trans.* **2020**. https://doi.org/10.1039/D0DT01256D.

Analysis of computed vs *experimentally determined pK_a values*

Analysis of the discrepancies between computed and experimentally determined data is very important, since there is an urgent need for trustable predicted data, when relationships are probable between structure, solution chemical properties (such as pK_a) and biological activity.

It should be noted that the prediction of the pK_a values works well when the additional moiety (with dissociative proton) is located at other positions than R7, thus far from the 8-hydroxquinoline-OH group (*e.g.* compounds **12**, **13**, **14**, **47**, **57**).

Having a closer look at the two compounds displayed in Figure S1, it can be seen that in the case of compound **38**, the possible intramolecular hydrogen bond between the protonated piperazinium nitrogen (NH^+, closer to the quinoline ring) and the deprotonated phenolate ($O^–$) decreases the pK_a (OH) value due to the stabilization of the conjugate base, whereas the pK_a of the $N_{piperazinium}H^+$ group is higher due to the same reason. For compound **9** as a consequence of the low pK_a values of piperazinium nitrogens the N-methyl nitrogen deprotonate at lower pH than the OH, and the possible OH…N hydrogen bond brings higher pK_a (OH) than computed one.

Like for the piperazinium in case of e.g. compound **38**, also in case of the piperidine moiety at R7 (compounds **3**, **19**, **20**, **24**, **97**), the experimentally determined pK_a (OH) is significantly lower than the computed one. Most probably the possible intramolecular hydrogen bond between the protonated piperidinium nitrogen (NH^+) and the deprotonated phenolate ($O^–$) results in the diminished pK_a (OH) value due to the stabilization of the conjugate base, while the pK_a of the NH^+ group is higher due to the same reason. The effect of the tetrahydroquinoline moiety at R7 position depends on the location of the benzene annulation. For the 2,3-benzo-annulation in compound **51**, the same is observed as in the case of the compounds with the piperidine moiety at R7; whereas the 3,4-benzo-annulation in compound **79** results in higher pK_a (OH) value than the computed one due to the low pK_a of the piperidinium-NH^+ (1.69). Since the intramolecular hydrogen bond is possibly formed between the deprotonated piperidine nitrogen and the protonated phenol group resulting in a more difficult release of the OH proton. As both the NH^+ groups in the N-methyl piperazine moiety at R7 position of compound **9** deprotonate at lower pH than the OH, the possible OH…N hydrogen bond results in higher pK_a (OH) than computed one. Similarly, the moderate pK_a value of the morpholinium nitrogen (compounds **8** (6.25), **29** (5.83), **108** (5.53)) has the same effect.

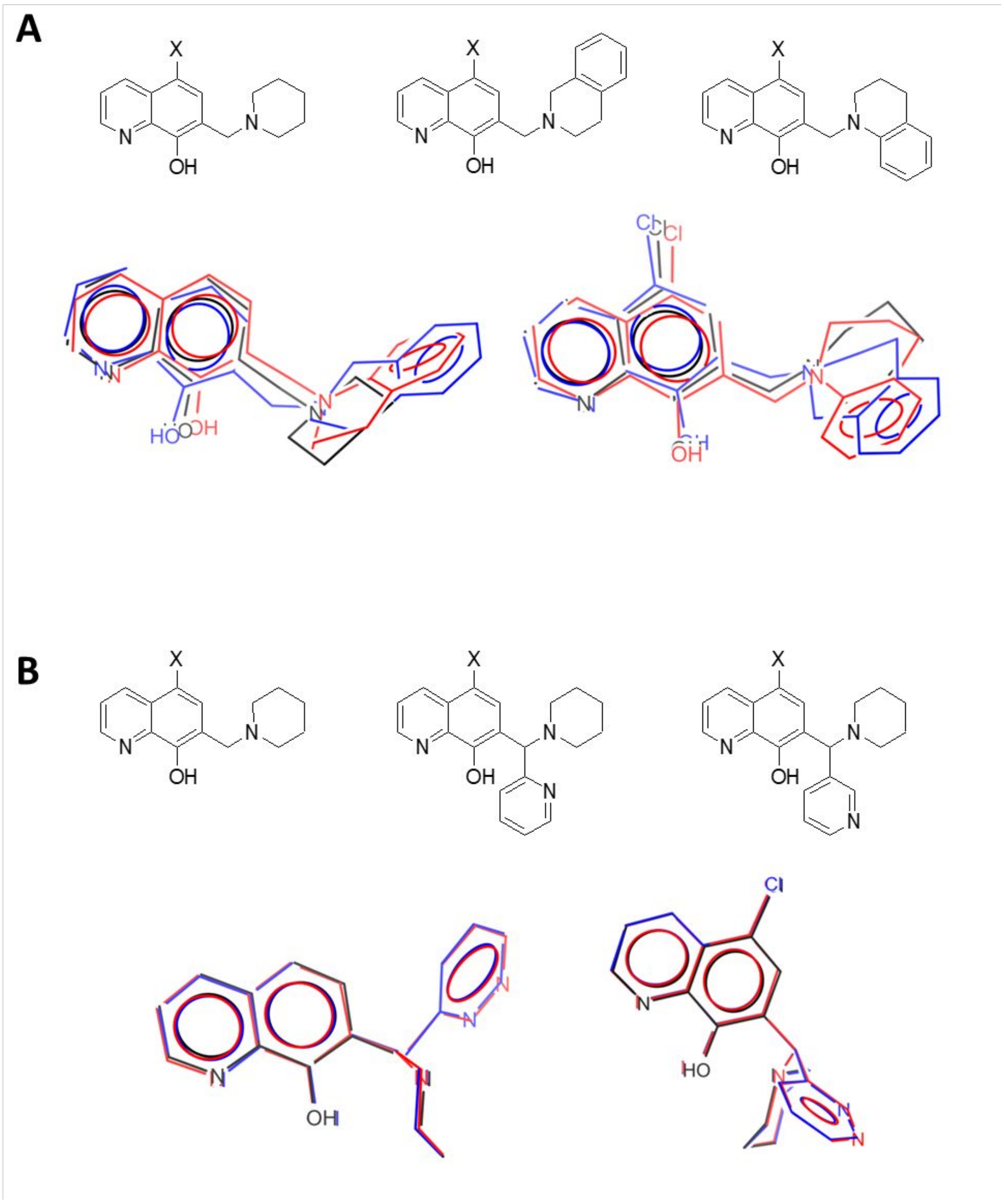

Figure S2: 3D alignment of (A) ligands **3** (black), **51** (blue) and **79** (red) and the R5-chloro substituted ligands **19** (black), **80** (blue) and (**83**) (red); and (B) of ligands **3** (black), (**122**) (blue) and **92** (red) and the R5-chloro substituted ligands **19** (black), **98** (blue) and 5-chloro-7-((piperidin-1-yl)(pyridin-3-yl)methyl)quinolin-8-ol (**123**) (red). Notably, compounds (**83**), (**122**) and (**123**) were not obtained and tested for biological activity, but merely involved in the chemoinformatic analysis. While ring annulation does not have a major steric effect on the chelation moiety, introduction of aromatic rings at the methylene carbon shields the donor atoms from two sides. Of note, 3D alignments are calculated in gas phase and do not take the protonation state of the respective compounds in solution into consideration.

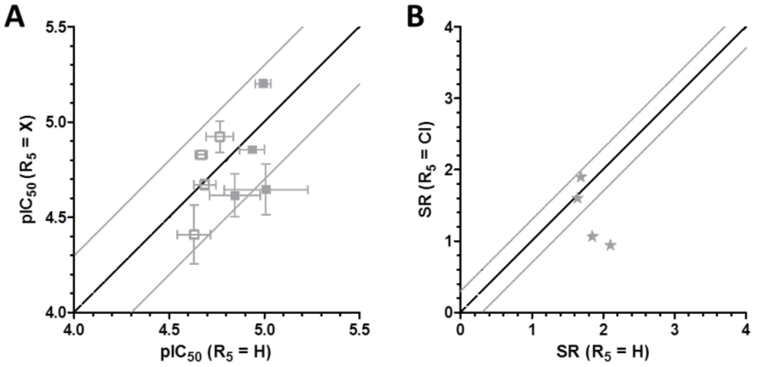

Figure S3: comparing the toxicity (A) and selectivity (B) of MMPs bearing an aromatic ring at the methylene carbon with and without chloro-substituent in R5. Bisecting lines reflect values with equal potency of compounds with and without substituents in R5. Toxicity is shown as pIC_{50} values of MMPs with different substituents in R5 (substituents on y-axis, H on x-axis) against MES-SA (open symbols) and MES-SA/Dx5 cells (filled symbols).

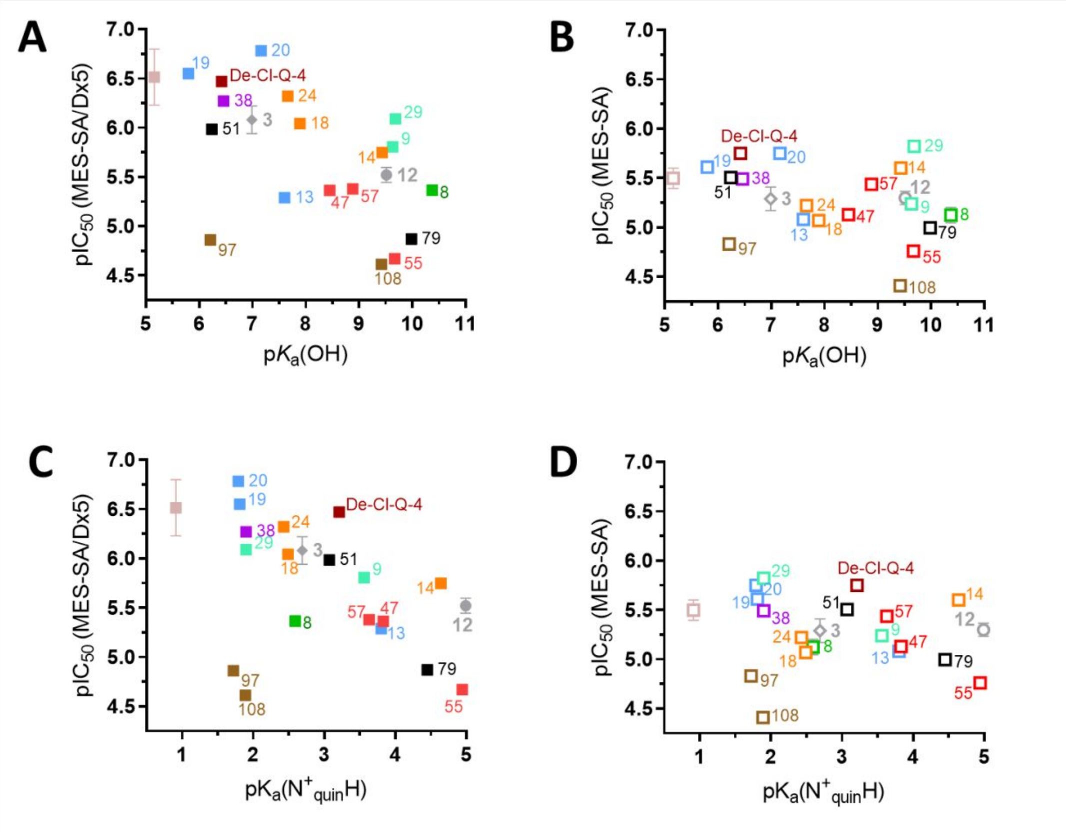

Figure S4: Correlation of toxicity, displayed as pIC_{50} values obtained in MDR MES-SA/Dx5 (A, C, filled symbols), and parental MES-SA cells (B,D, open symbols) with pK_a values of the hydroxyl group (A,B) and the quinolinium nitrogen (C,D). Color coding represents the following structural modifications presented in the manuscript: introduction of halogen atom in R5: light blue; introduction of a hetero-atom in R7: green; introduction of a hetero-atom in R7 and a halogen in R5: cyan; introduction of a heter-atom in R7 as a linker connecting an aromatic ring: purple; introduction of an alkoxyalkyl residue in R5: orange; substitution in R5 instead of R7: red; introduction of an annulated aromatic ring: black; introduction of an aromatic ring at the methylene bridge: brown; benzylamine substituent in R7 (secondary amine from previous study): bordeaux.

Table S5: Effect of aromatic aldehyde moieties in comparison to formaldehyde derived Mannich bases with tertiary amines (extended version of Figure 7).

compound	R^5	R aldehyde	R amine	IC_{50} / µM MES-SA	IC_{50} / µM MES-SA/Dx5	SR
**2**	-H	-H	-pyrrolidine	4.64 ± 0.82	1.47 ± 0.25	3.16
**84**	-H	-2-pyridyl	-pyrrolidine	17.24 ± 1.86	10.18 ± 0.60	1.69
**85**	-H	-3-pyridyl	-pyrrolidine	41.02 ±	23.28 ±	1.76
**86**	-H	-4-pyridyl	-pyrrolidine	5.99 ± 0.88	3.66 ± 0.45	1.64
**87**	-H	-4-chlorophenyl	-pyrrolidine	19.72 ± 2.25	6.58 ± 0.53	3.00
**88**	-H	-2-bromophenyl	-pyrrolidine	13.04 ± 1.35	6.60 ± 0.21	1.98
**89**	-H	-4-trifluormethylphenyl	-pyrrolidine	11.08 ± 3.57	2.83 ± 0.61	3.91
**15**	-Cl	-H	-pyrrolidine	3.46 ± 0.51	0.30 ± 0.06	11.53
**90**	-Cl	-2-pyridyl	-pyrrolidine	12.03 ± 1.50	6.30 ± 0.15	1.91
**91**	-Cl	-4-tolyl	-pyrrolidine	13.23 ± 3.09	6.06 ± 2.21	2.18
**3**	-H	-H	-piperidine	5.21 ± 0.95	0.85 ± 0.18	6.13
**92**	-H	-3-pyridyl	-piperidine	13.65 ± 0.12	8.58 ± 0.38	1.59
**93**	-H	-4-pyridyl	-piperidine	16.22 ± 4.33	8.39 ± 1.09	1.93
**94**	-H	-2-thiophenyl	-piperidine	8.88 ± 0.93	11.63 ± 0.94	0.76
**95**	-H	-2-chlorophenyl	-piperidine	17.48 ± 4.45	9.45 ± 2.11	1.85
**96**	-H	-4-fluorophenyl	-piperidine	22.71 ± 0.86	14.04 ± 1.50	1.62
**19**	-Cl	-H	-piperidine	2.49 ± 0.39	0.29 ± 0.05	8.71
**97**	-Cl	-phenyl	-piperidine	14.86 ± 0.18	13.96 ± 0.15	1.06
**98**	-Cl	-2-pyridyl	-piperidine	31.88 ± 1.55	16.58 ± 0.88	1.92
**99**	-H	-phenyl	-4-methyl-piperidine	8.77 ± 0.24	11.48 ± 1.75	0.76
**100**	-H	-4-pyridyl	-4-methyl-piperidine	17.10 ± 3.89	6.24 ± 1.17	2.74
**101**	-H	-2-thiophenyl	-4-methyl-piperidine	10.79 ± 0.18	16.58 ± 0.81	0.65
**8**	-H	-H	-morpholine	7.55 ± 0.91	4.33 ± 0.31	1.75
**102**	-H	-phenyl	-morpholine	21.45 ± 1.01	11.67 ± 1.15	1.84
**103**	-H	-2-pyridyl	-morpholine	23.59 ± 3.13	14.58 ± 2.97	1.62
**104**	-H	-4-pyridyl	-morpholine	15.17 ± 2.74	7.14 ± 1.26	2.12
**105**	-H	-2-chlorophenyl	-morpholine	18.45 ± 0.14	16.36 ± 1.02	1.13
**106**	-H	-4-fluorophenyl	-morpholine	33.44 ± 13.16	14.90 ± 6.17	2.24
**107**	-H	-4-methoxyphenyl	-morpholine	20.30 ± 1.70	15.76 ± 2.97	1.29
**29**	-Cl	-H	-morpholine	1.52 ± 0.07	0.82 ± 0.06	1.86
**108**	-Cl	-2-pyridyl	-morpholine	40.00 ± 9.54	24.68 ± 4.26	1.62
**109**	-Cl	-3pyridyl	-morpholine	50.99 ± 2.48	32.87 ± 7.06	1.55
**110**	-Cl	-2-thiophenyl	-morpholine	7.97 ± 1.01	13.97 ± 0.42	0.57
**34**	-H	-H	-methyl-piperazine	16.70 ± 1.86	8.67 ± 1.69	1.93
**111**	-H	-3-pyridyl	-methyl-piperazine	54.82 ± 3.66	35.90 ± 4.93	1.53
**112**	-H	-4-pyridyl	-methyl-piperazine	22.14 ± 2.70	10.96 ± 2.12	2.02
**113**	-H	-2-thiophenyl	-methyl-piperazine	12.82 ± 1.72	16.79 ± 0.08	0.76
**9**	-Cl	-H	-methyl-piperazine	5.86 ± 0.98	1.59 ± 0.27	3.69
**114**	-Cl	-2-pyridyl	-methyl-piperazine	16.01 ± 1.31	13.88 ± 0.46	1.15
**115**	-H	-3-pyridyl	-ethyl-piperazine	20.64 ± 1.85	10.35 ± 3.54	1.99
**116**	-H	-2-thiophenyl	-ethyl-piperazine	9.06 ± 0.23	14.16 ± 1.36	0.64
**56**	-Cl	-H	-ethyl-piperazine	3.33 ± 0.38	1.18 ± 0.11	2.82
**117**	-Cl	-2-pyridyl	-ethyl-piperazine	28.28 ± 2.32	24.41 ± 3.58	1.16
**118**	-Cl	-3-pyridyl	-ethyl-piperazine	21.35 ± 0.84	23.11 ± 4.67	0.92
**119**	-H	-phenyl	-4-(2-hydroxyethyl)-piperazine	20.68 ± 0.97	13.54 ± 2.55	1.53
**120**	-H	-4-fluorophenyl	-4-(2-hydroxyethyl)-piperazine	66.68 ± 4.56	29.08 ± 7.12	2.29
**121**	-H	-2-pyridyl	-4-(2-pyridyl)-piperazine	40.93 ± 2.86	22.57 ± 2.85	1.81

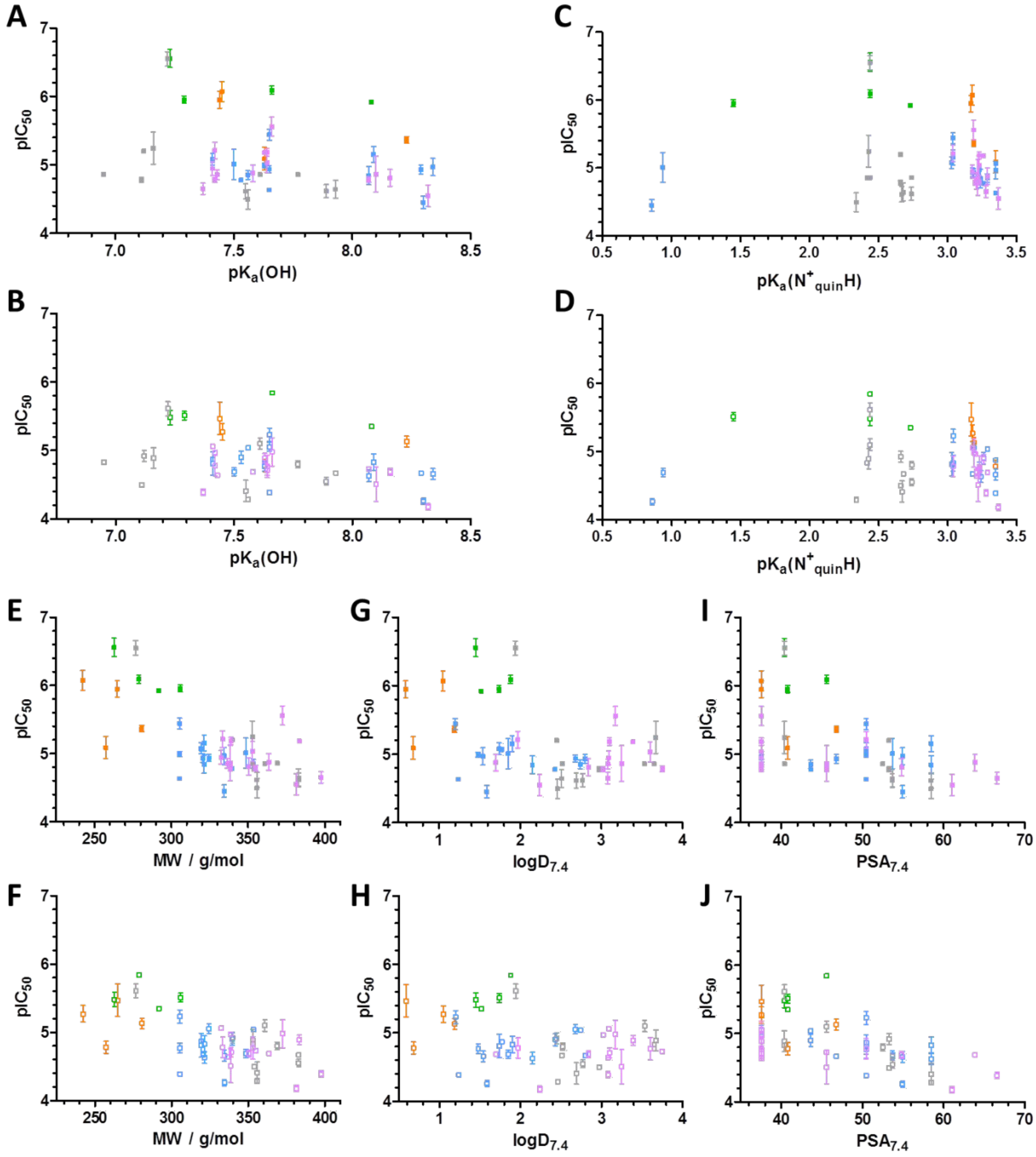

Figure S5: Impact of the calculated chemical properties pK_a(OH) (A,B), pK_a(N^+_{quin}H) (C,D), molecular weight MW (E,F), logD at pH 7.4 (G,H) and the polar surface area at pH 7.4 (I,J) on the toxicity profile of 47 8-hydroxyquinoline derivatives against MES-SA/Dx5 (A, C, E, G, I) and MES-SA (B, D, F, H, J) cells. A linear correlation was calculated over all values in case of panels A and B. Color coding distinguishes the following compound classes: derivatives without R5 substitution, obtained from formaldehyde (listed in Figure 7: orange), or from aromatic aldehydes (listed in Figure 7: blue, listed in Table S5: rose), as well as derivatives with Chloro-substituent in R5 obtained from formaldehyde (green), or aromatic aldehydes (grey).

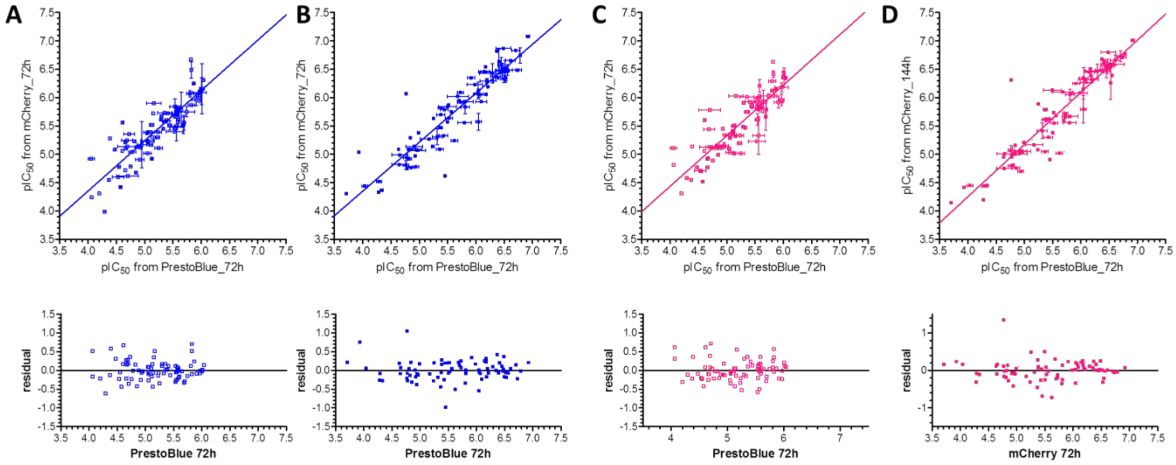

**Data status 20160303**	MES-SA_mCherry 72h	Dx5_mCherry 72h	MES-SA_mCherry_144h	Dx5_mCherry_144h
Best-fit values				
Slope	0.8877 ± 0.05732	0.8638 ± 0.04108	0.8917 ± 0.05955	0.9206 ± 0.04014
Y-intercept when X=0.0	0.8006 ± 0.2989	0.8946 ± 0.2320	0.8698 ± 0.3104	0.5695 ± 0.2268
X-intercept when Y=0.0	-0,9018	-1,036	-0,9754	-0,6187
1/slope	1,126	1,158	1,121	1,086
Goodness of Fit				
r^2	0,7546	0,8517	0,7444	0,8723
P value	< 0.0001	< 0.0001	< 0.0001	< 0.0001
Number of X values	80	79	79	79

Figure S6: Comparing the effect of 80 investigated chelators obtained in different assays in MES-SA (A, C) and MES-SA/Dx5 (B, D) cells. Displayed are results of PrestoBlue assay vs. mCherry measurements after 72 h (A, B) and 144h (C,D) respectively, together with the residual plots of the respective linear fits.

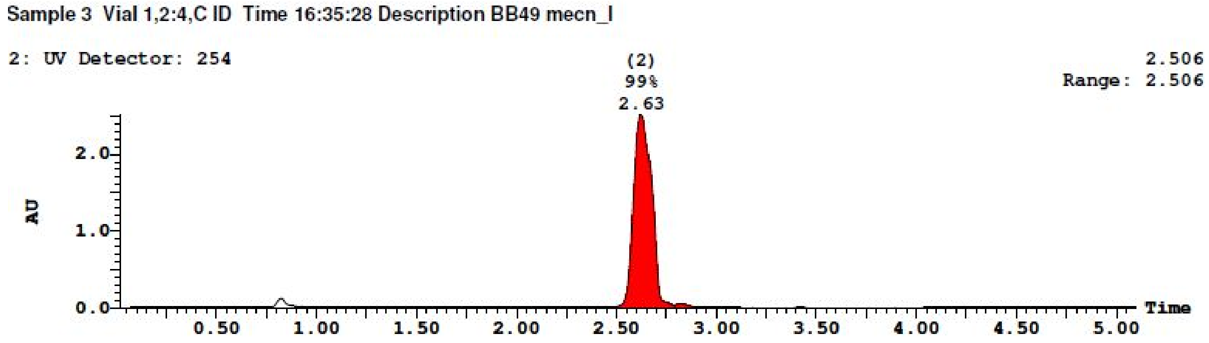

Figure S7: HPLC-trace of compound **34** (Experiment#: BB49), indicating a purity of 99 %.

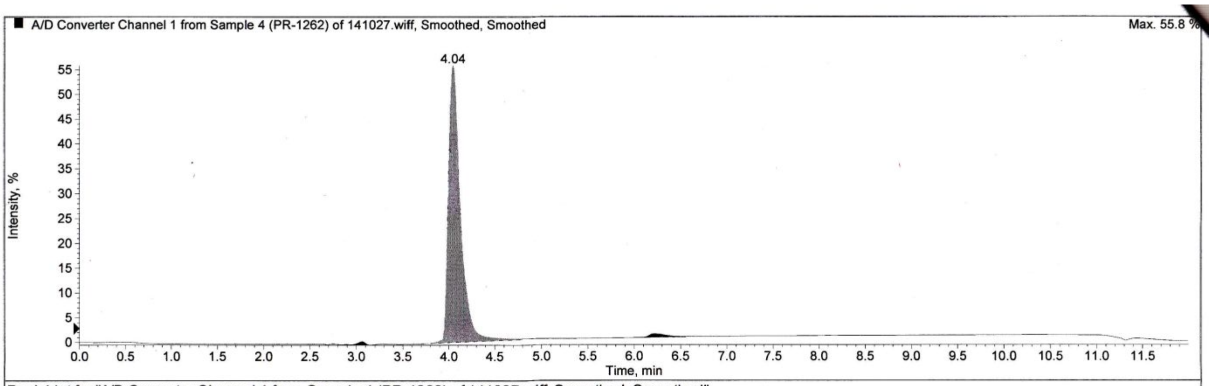

Peak List for "A/D Converter Channel 1 from Sample 4 (PR-1262) of 141027.wiff, Smoothed, Smoothed"

	Time (min)	Area (counts)	%Area	Height (%)	%Height	Width (min)	Baseline Type
1	3.0635	3.8009	0.7146	0.7038	1.2299	0.2000	Base to Base
2	4.0393	518.8708	97.5577	55.8219	97.5429	1.0900	Valley
3	6.2177	9.1888	1.7277	0.7023	1.2272	0.6100	Valley

Figure S8: HLPC trace of compound **16** (Experiment#: PR-1262), recorded at 254nm, indicating a purity of 97.56 %.

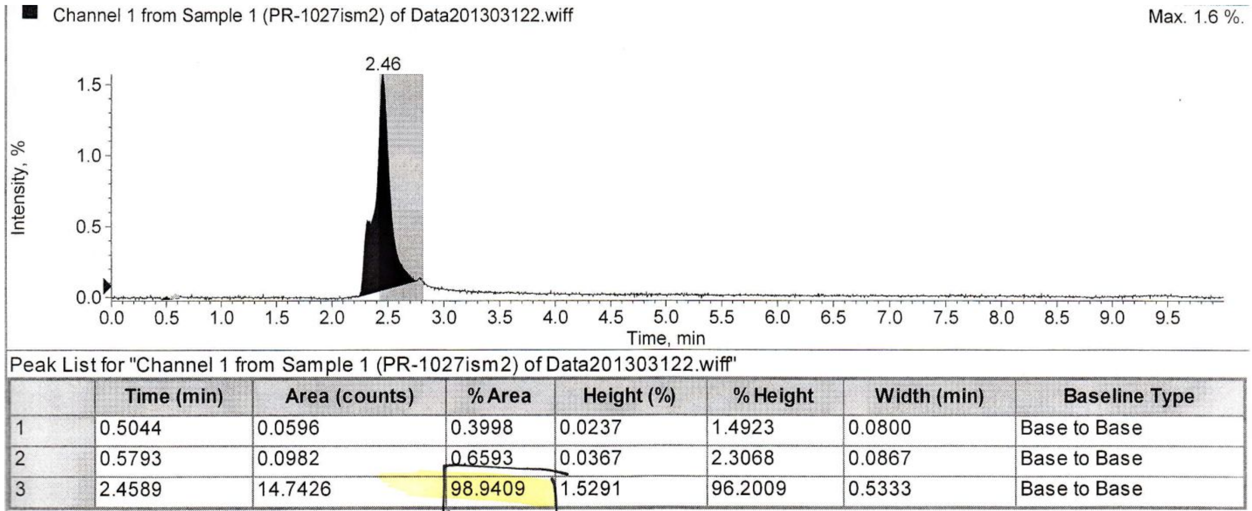

Peak List for "Channel 1 from Sample 1 (PR-1027ism2) of Data201303122.wiff"

	Time (min)	Area (counts)	%Area	Height (%)	%Height	Width (min)	Baseline Type
1	0.5044	0.0596	0.3998	0.0237	1.4923	0.0800	Base to Base
2	0.5793	0.0982	0.6593	0.0367	2.3068	0.0867	Base to Base
3	2.4589	14.7426	98.9409	1.5291	96.2009	0.5333	Base to Base

Figure S9: HLPC trace of compound **82** (Experiment#: PR-1027ism2), recorded at 254nm, indicating a purity of 98.94 %.

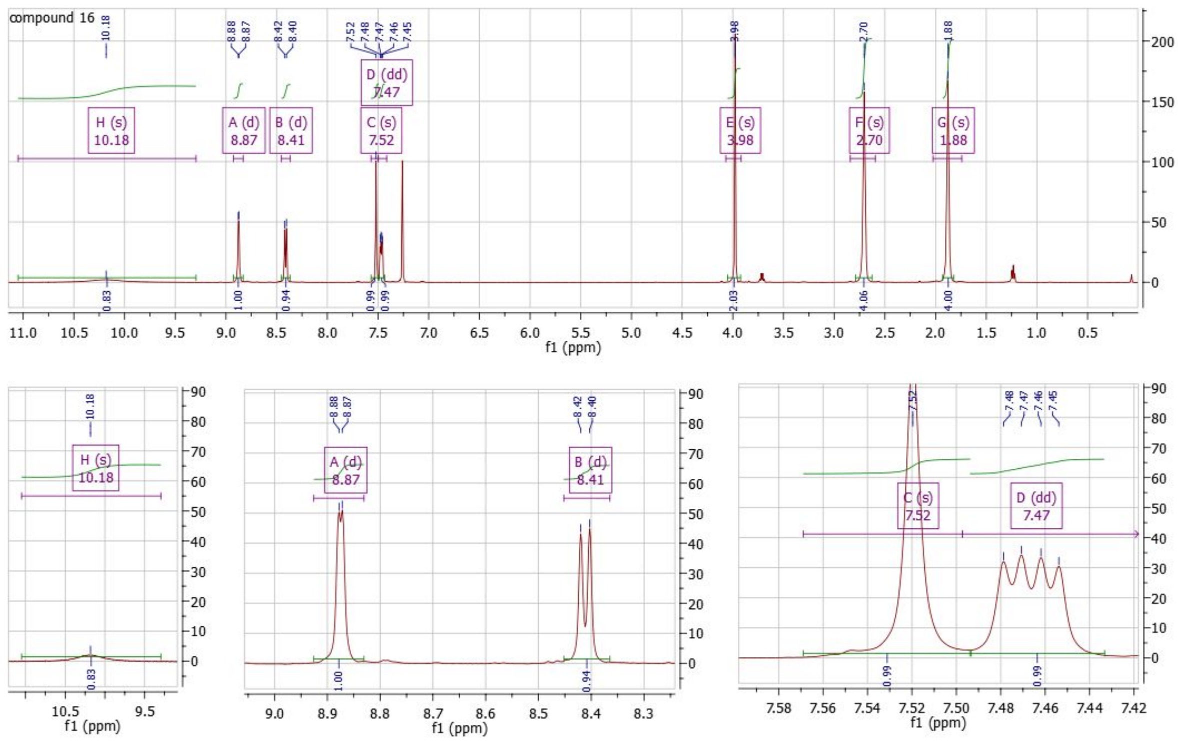

Figure S10: ^{1}H NMR spectrum of 5-bromo-7-(pyrrolidin-1-ylmethyl)quinolin-8-ol (**16**)

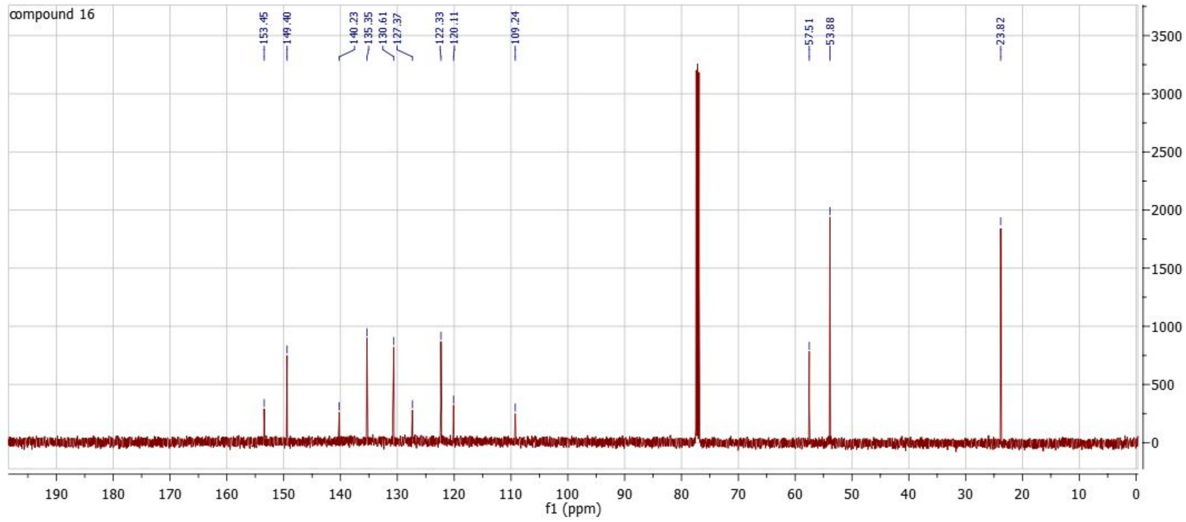

Figure S11: ^{13}C NMR spectrum of 5-bromo-7-(pyrrolidin-1-ylmethyl)quinolin-8-ol (**16**)

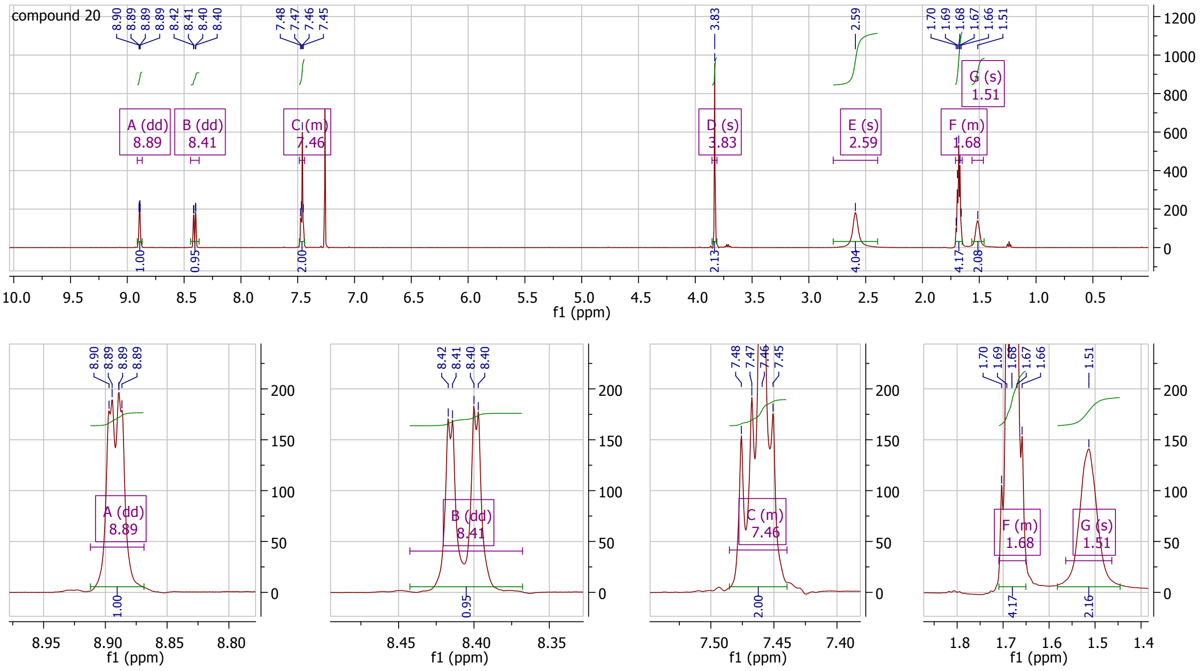

Figure S12: ^{1}H NMR spectrum of 5-bromo-7-(piperidin-1-ylmethyl)quinolin-8-ol (**20**).

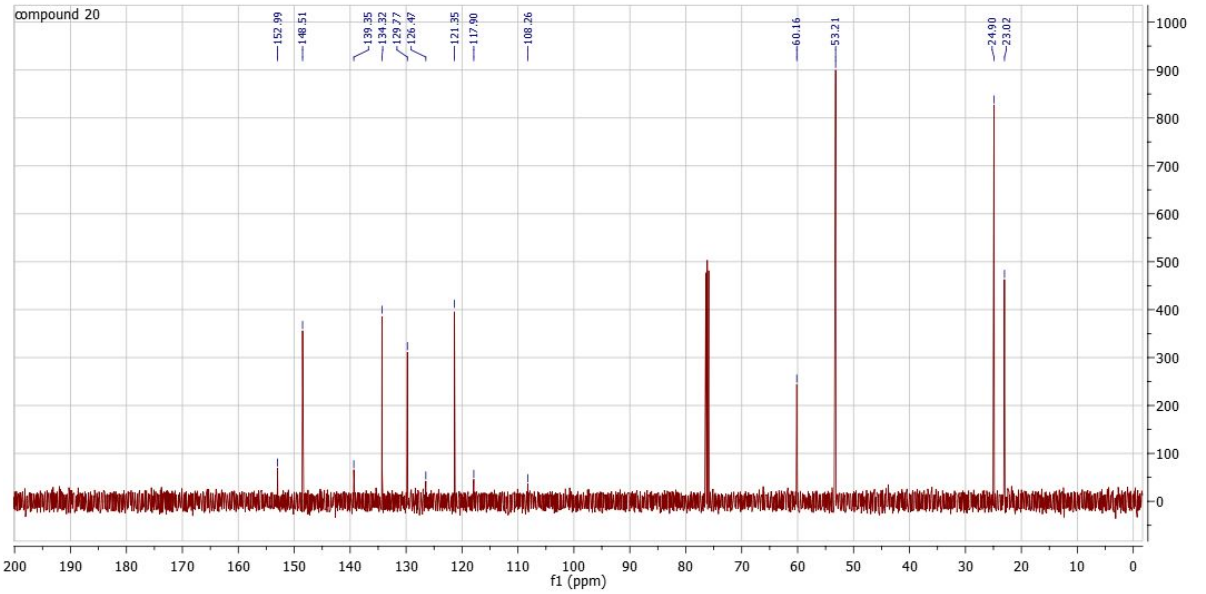

Figure S13: ^{13}C NMR spectrum of 5-bromo-7-(piperidin-1-ylmethyl)quinolin-8-ol (**20**)

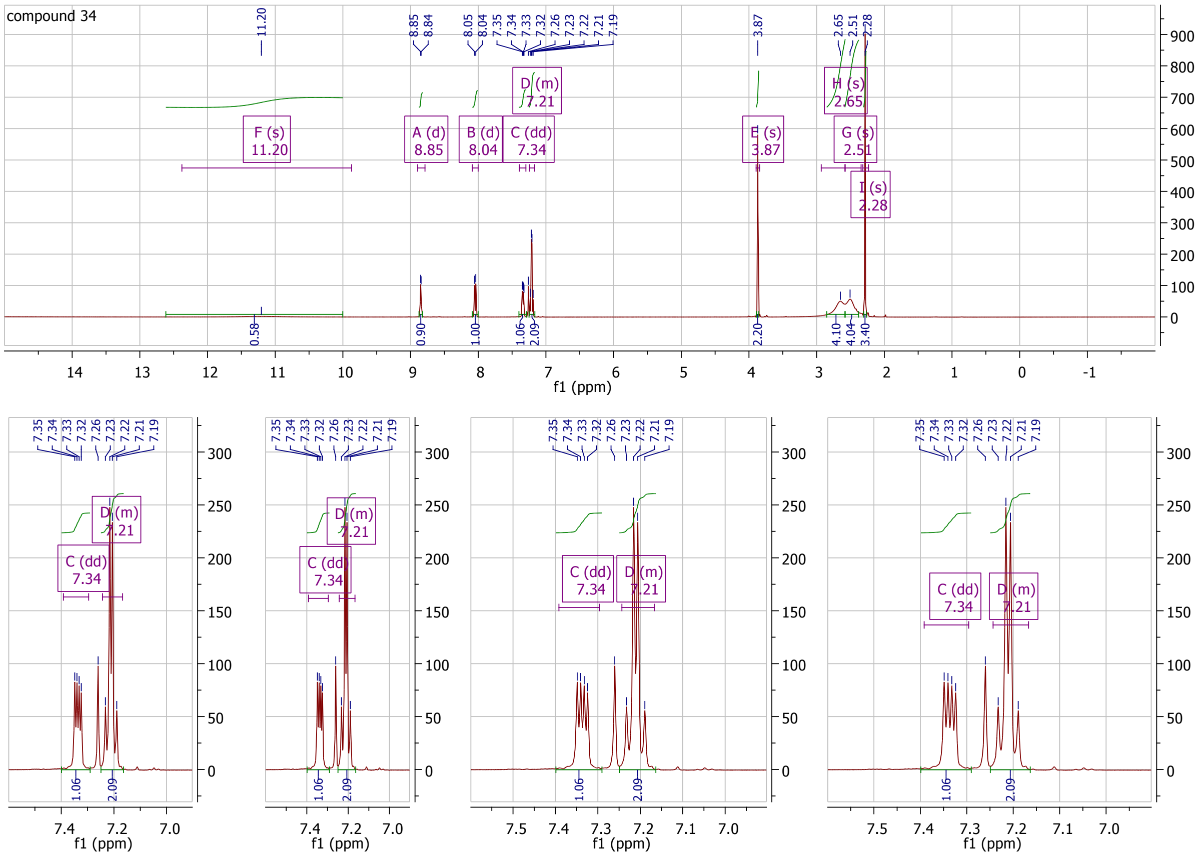

Figure S14: ^{1}H NMR spectrum of 7-((4-methylpiperazin-1-yl)methyl)quinolin-8-ol (**34**)

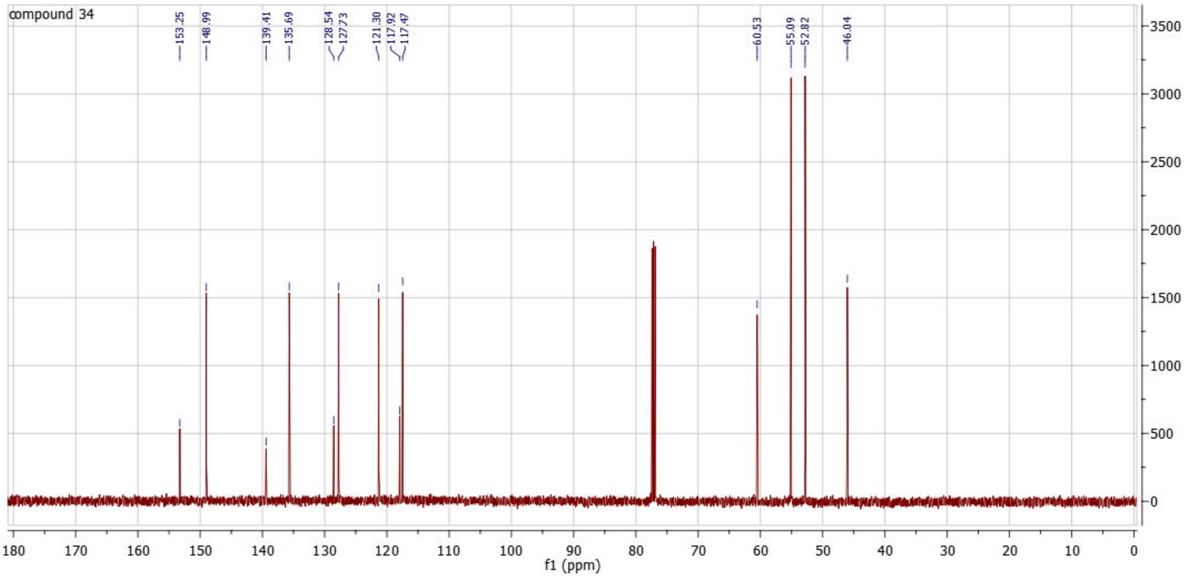

Figure S15: ^{13}C NMR spectrum of 7-((4-methylpiperazin-1-yl)methyl)quinolin-8-ol (**34**)

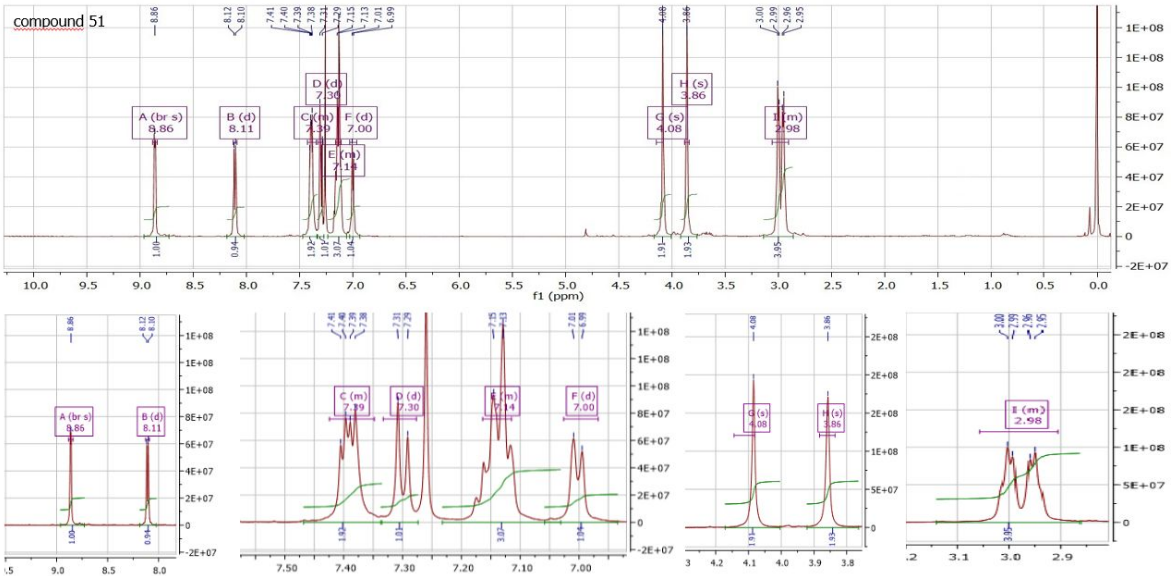

Figure S16: ^{1}H NMR spectrum of 7-((3,4-dihydroisoquinolin-2(1H)-yl)methyl)quinolin-8-ol (**51**)

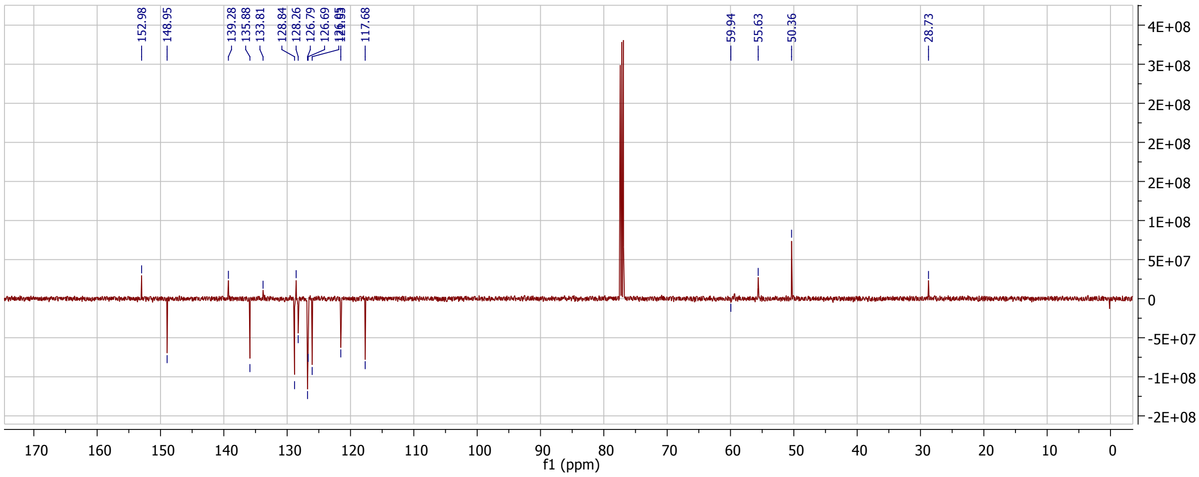

Figure S17: ^{13}C NMR spectrum of 7-((3,4-dihydroisoquinolin-2(1H)-yl)methyl)quinolin-8-ol (**51**)

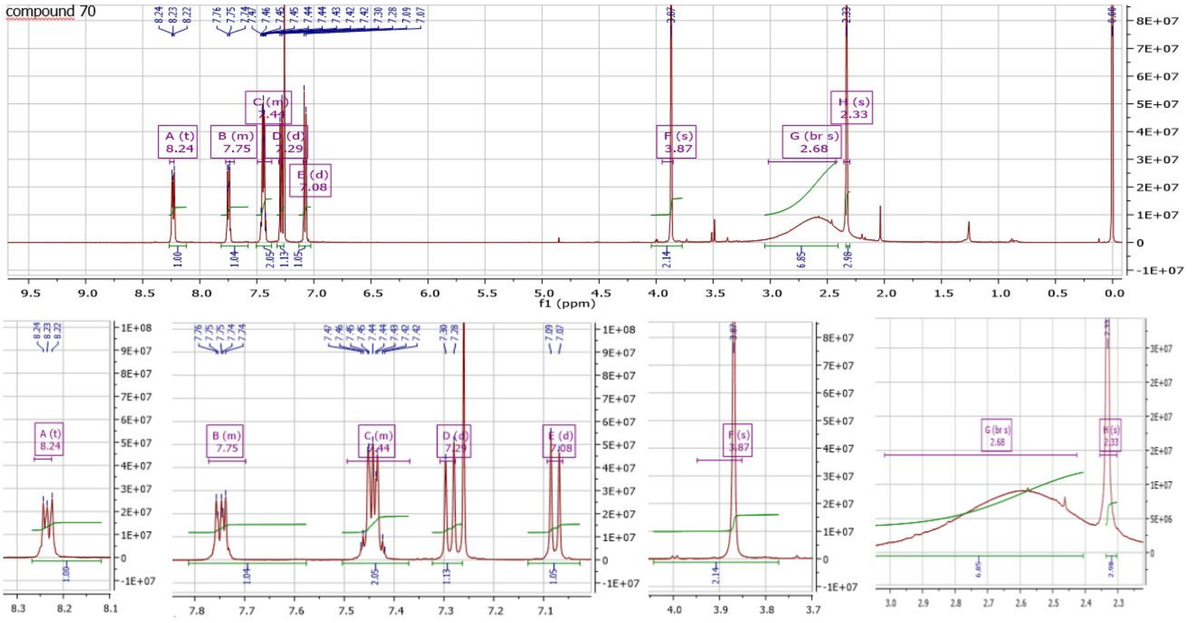

Figure S18: ^{1}H NMR spectrum of 2-((4-methylpiperazin-1-yl)methyl)naphthalen-1-ol (**70**)

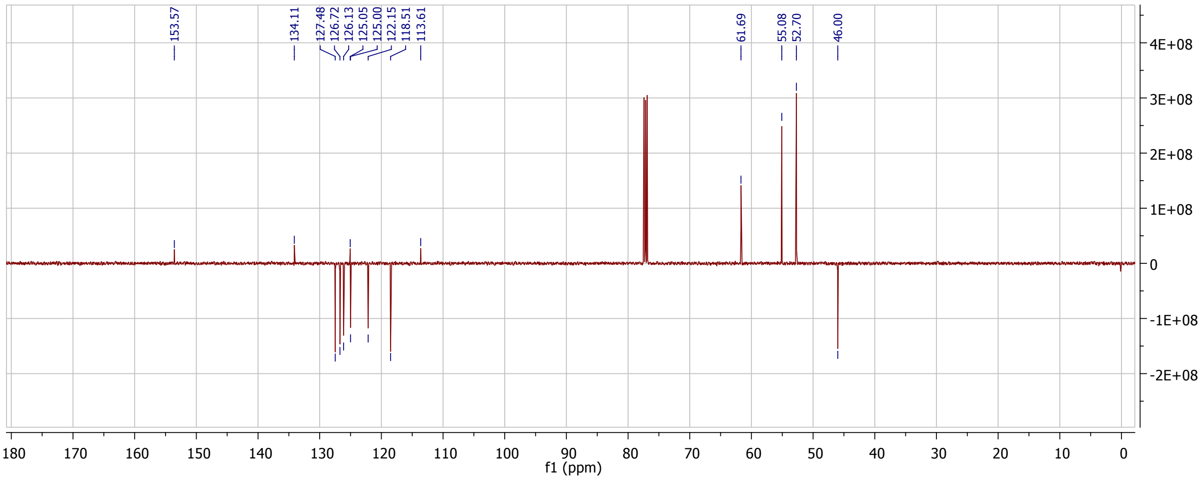

Figure S19: ^{13}C NMR spectrum of 2-((4-methylpiperazin-1-yl)methyl)naphthalen-1-ol (**70**)

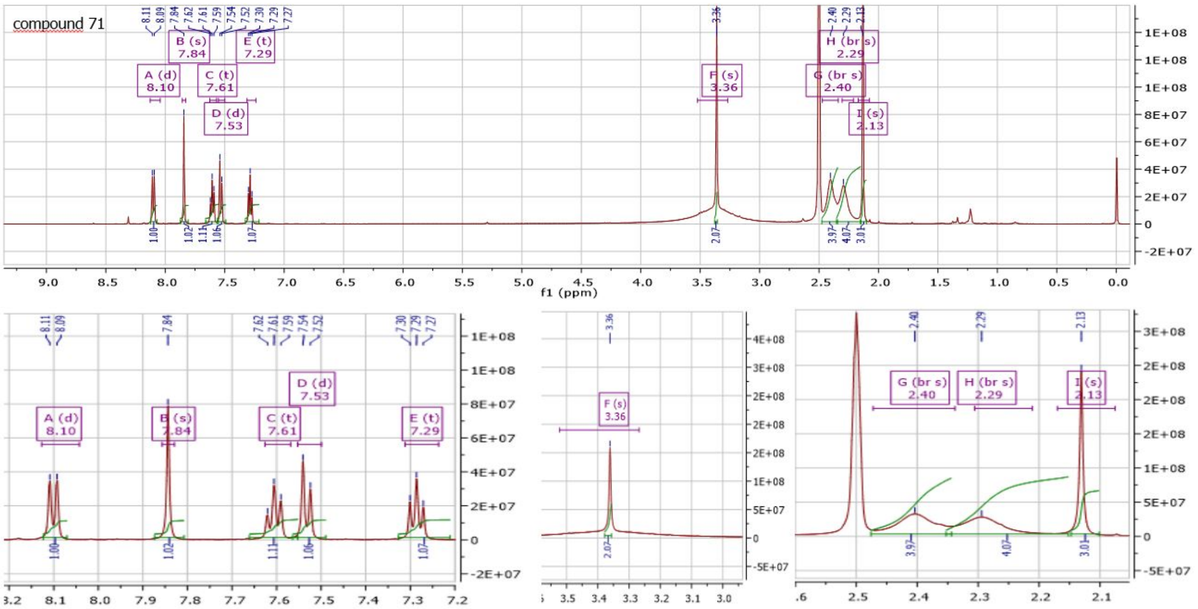

Figure S20: ^{1}H NMR spectrum of 3-((4-methylpiperazin-1-yl)methyl)quinolin-4-ol (**71**)

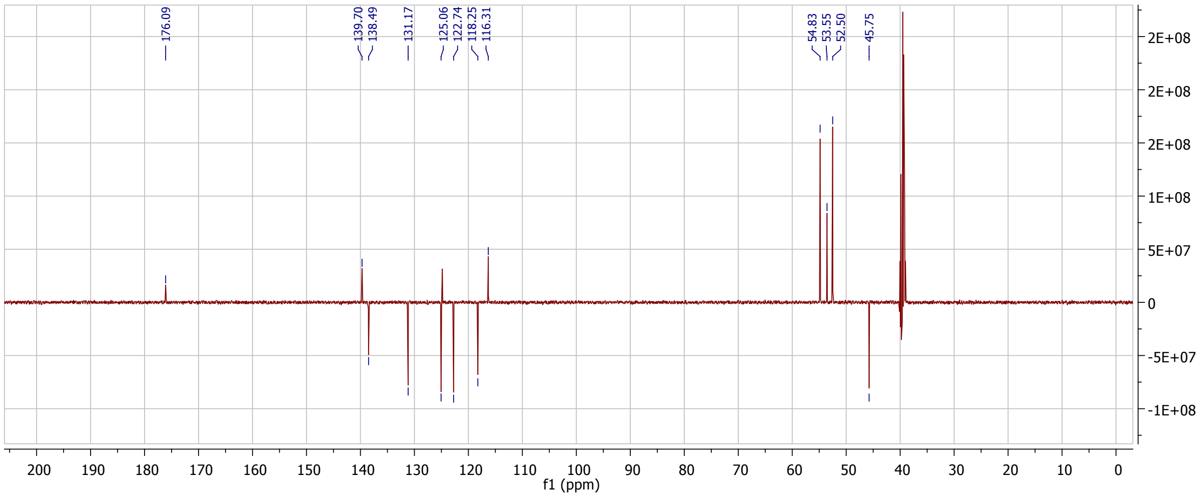

Figure S21: ^{13}C NMR spectrum of 3-((4-methylpiperazin-1-yl)methyl)quinolin-4-ol (**71**)

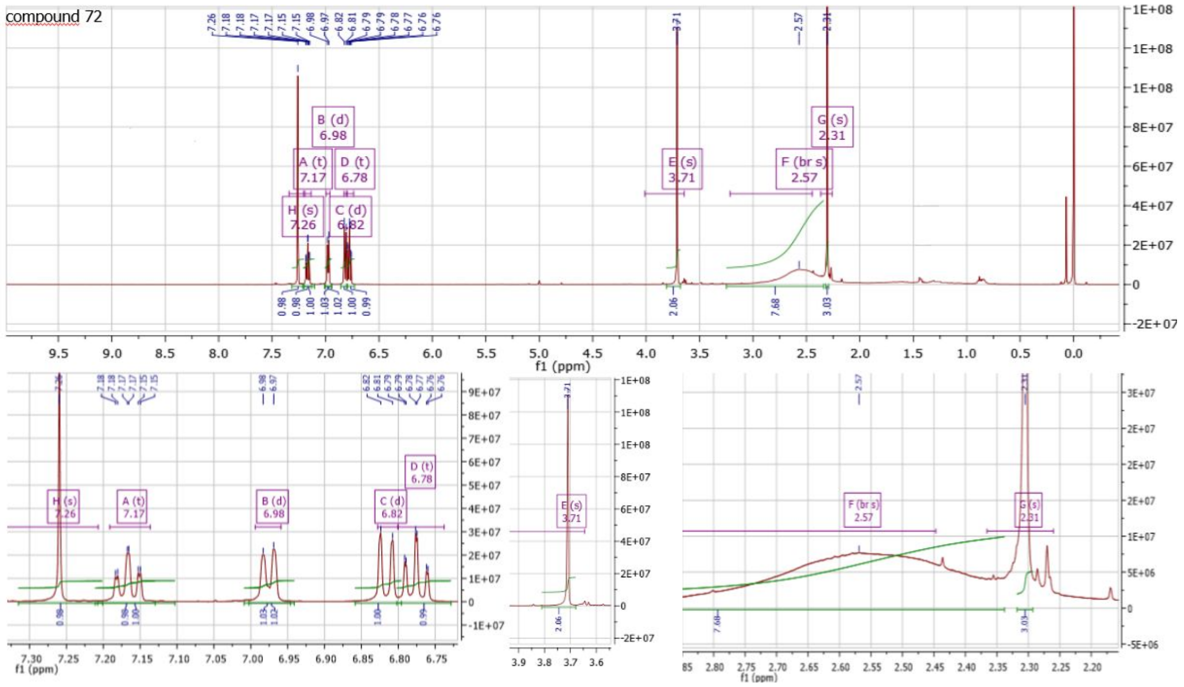

Figure S22: ^{1}H NMR spectrum of 2-((4-methylpiperazin-1-yl)methyl)phenol (**72**)

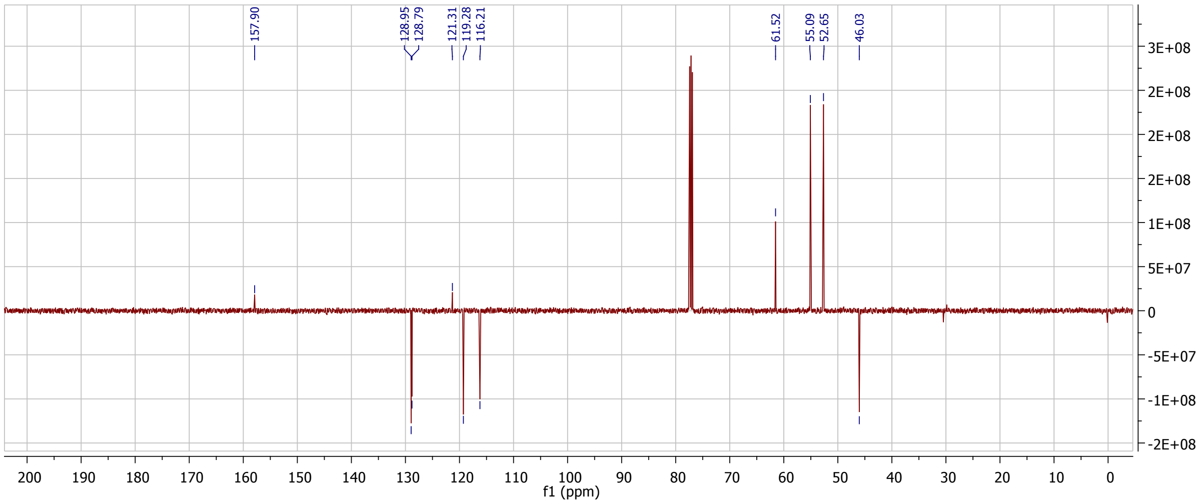

Figure S23: ^{13}C NMR spectrum of 2-((4-methylpiperazin-1-yl)methyl)phenol (**72**)

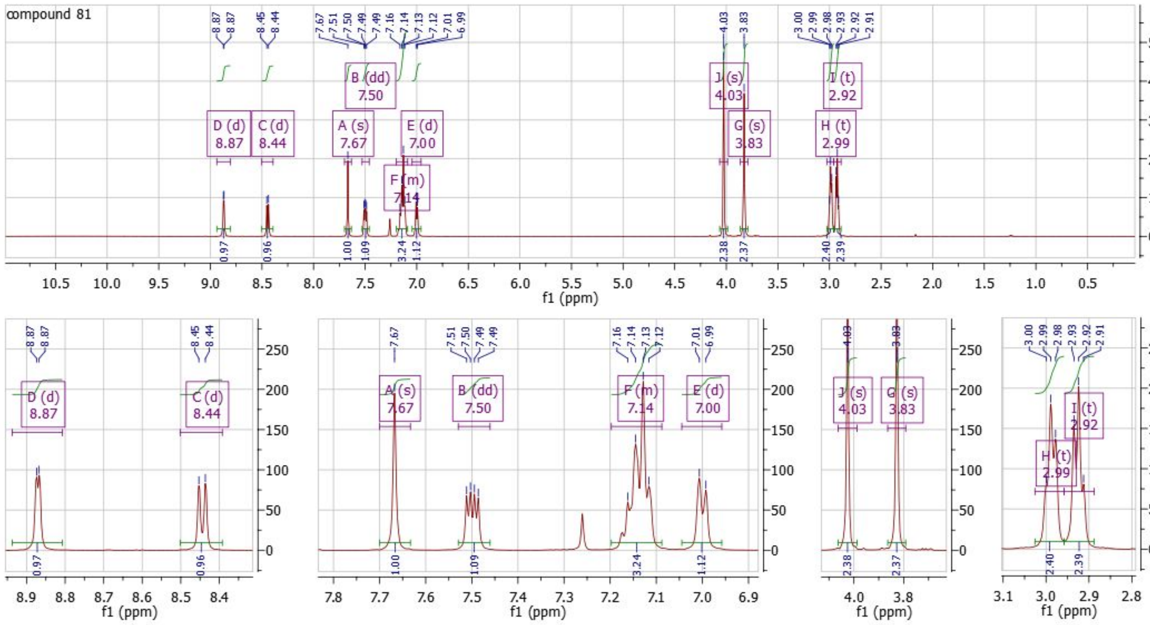

Figure S24: ^{1}H NMR spectrum of 5-bromo-7-((3’,4’-dihydroisoquinolin-2’(1’H)-yl)methyl)quinolin-8-ol (**81**)

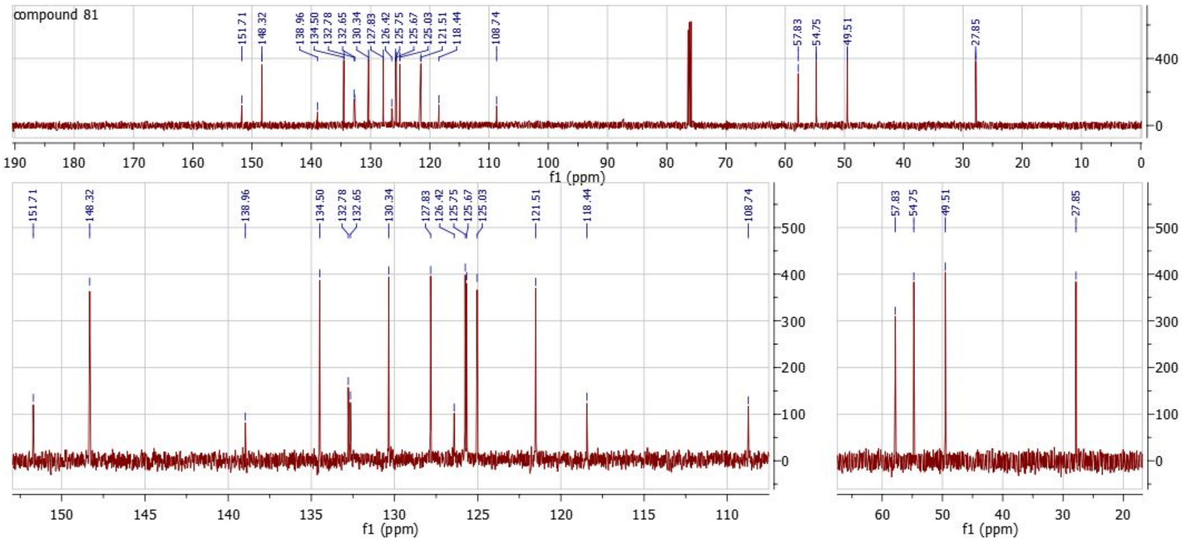

Figure S25: ^{13}C NMR spectrum of 5-bromo-7-((3’,4’-dihydroisoquinolin-2’(1’H)-yl)methyl)quinolin-8-ol (**81**)

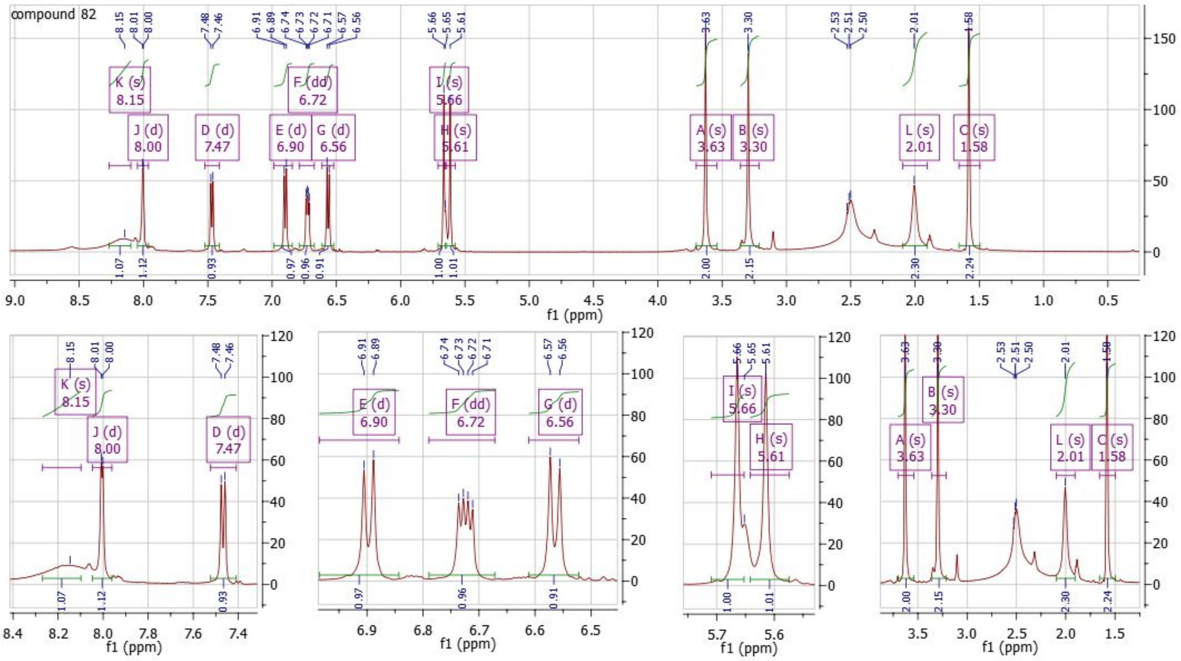

Figure S26: ^{1}H NMR spectrum of 2'-((8-hydroxy-quinolin-7-yl)methyl)-1',2',3',4'-tetrahydroisoquinoline-6',7'-diol hydrochloride (**82**)

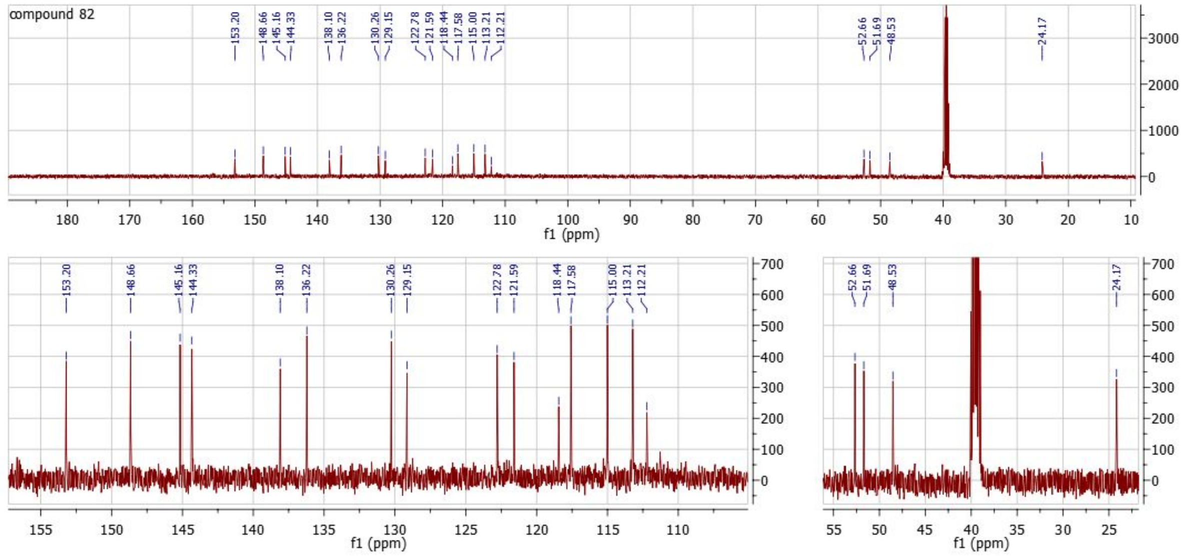

Figure S27: ^{13}C NMR spectrum of 2'-((8-hydroxy-quinolin-7-yl)methyl)-1',2',3',4'-tetrahydroisoquinoline-6',7'-diol hydrochloride (**82**)

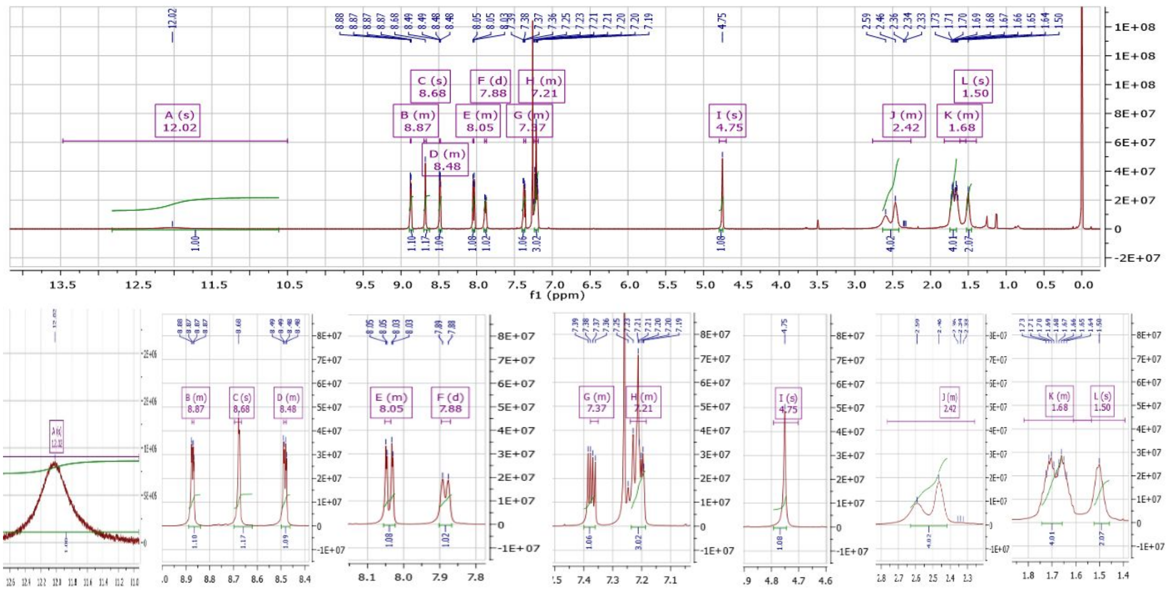

Figure S28: ^{1}H NMR spectrum of 7-(piperidine-1-yl)(pyridin-3-yl)methyl)quinolin-8-ol (**92**)

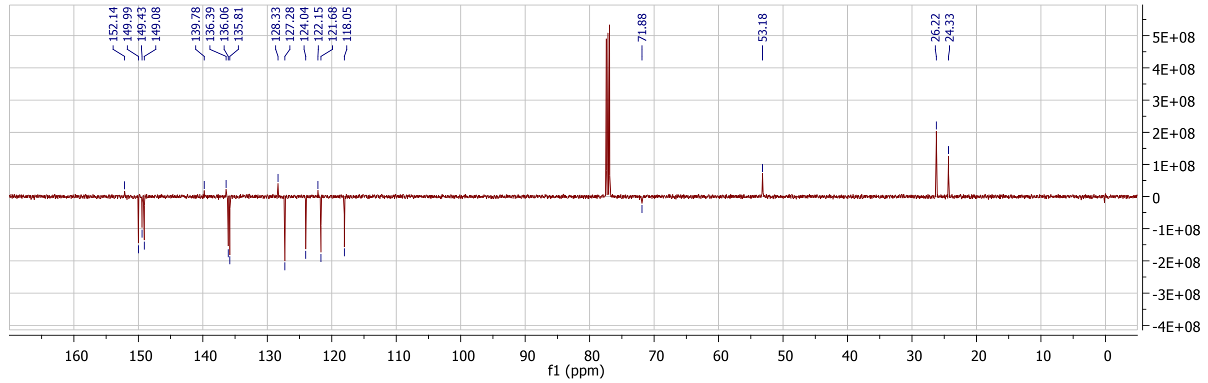

Figure S29: ^{13}C NMR spectrum of 7-(piperidine-1-yl)(pyridin-3-yl)methyl)quinolin-8-ol (**92**)

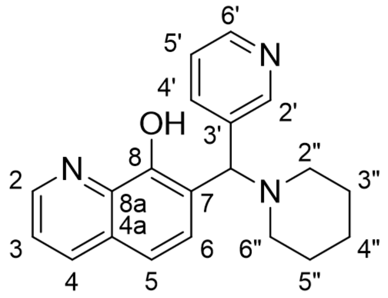

Figure S30: Structure of 7-(piperidine-1-yl)(pyridin-3-yl)methyl)quinolin-8-ol (**92**)

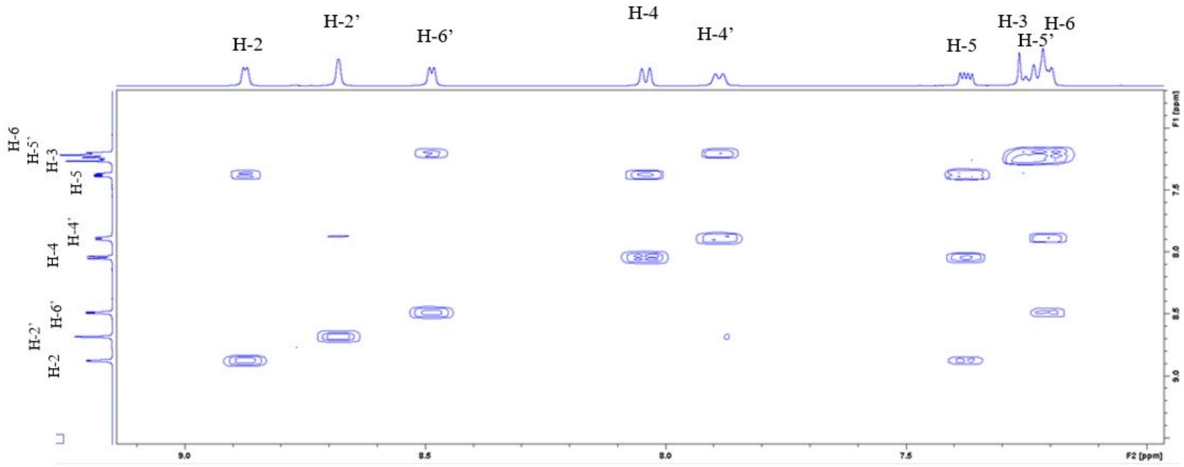

Figure S31: COSY NMR spectrum of 7-(piperidine-1-yl)(pyridin-3-yl)methyl)quinolin-8-ol (**92**)

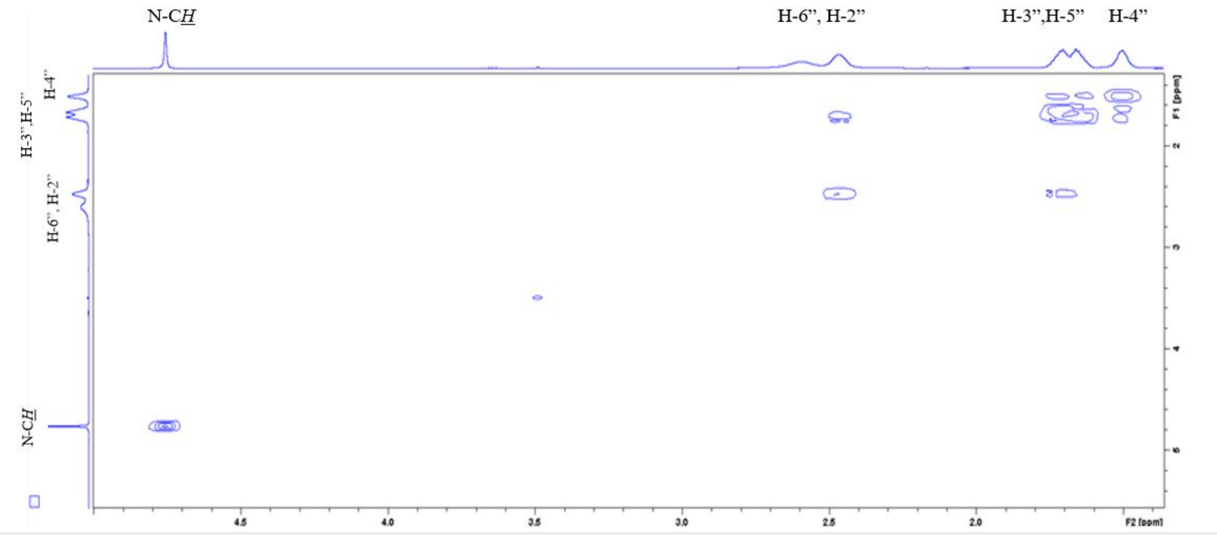

Figure S32: COSY NMR spectrum of 7-(piperidine-1-yl)(pyridin-3-yl)methyl)quinolin-8-ol (**92**)

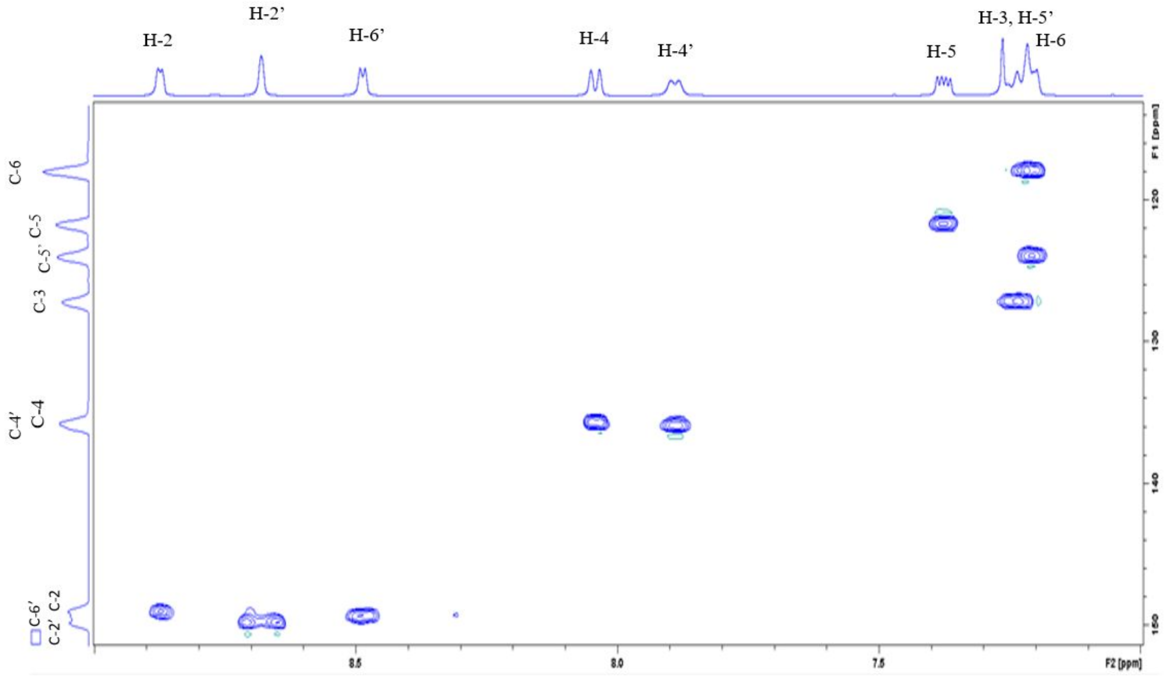

Figure S33: HSQC NMR spectrum of 7-(piperidine-1-yl)(pyridin-3-yl)methyl)quinolin-8-ol (**92**)

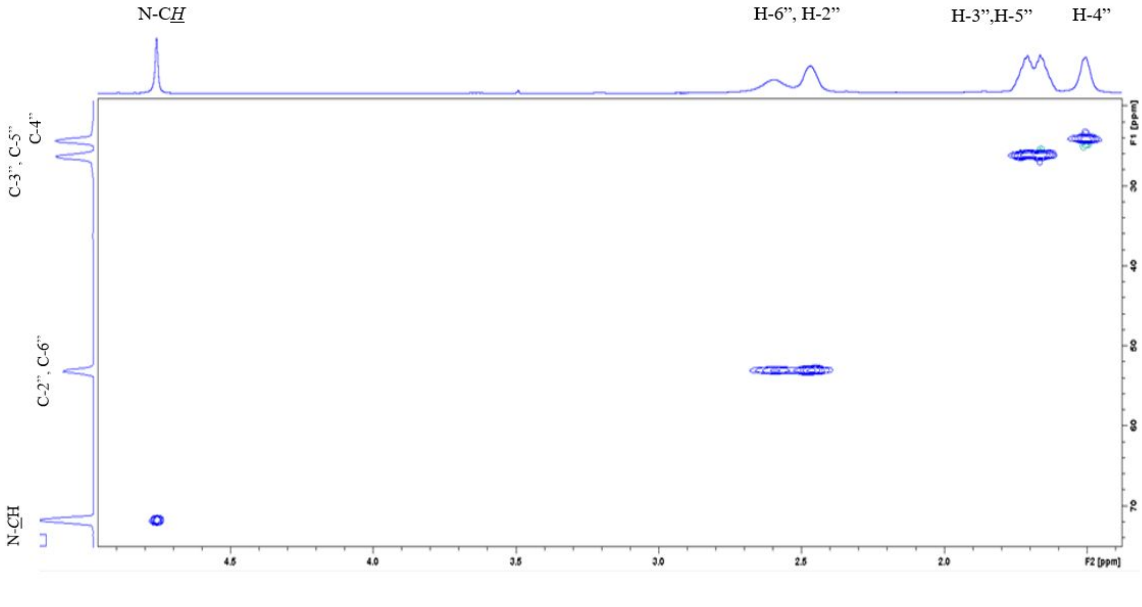

Figure S34: HSQC NMR spectrum of 7-(piperidine-1-yl)(pyridin-3-yl)methyl)quinolin-8-ol (**92**)

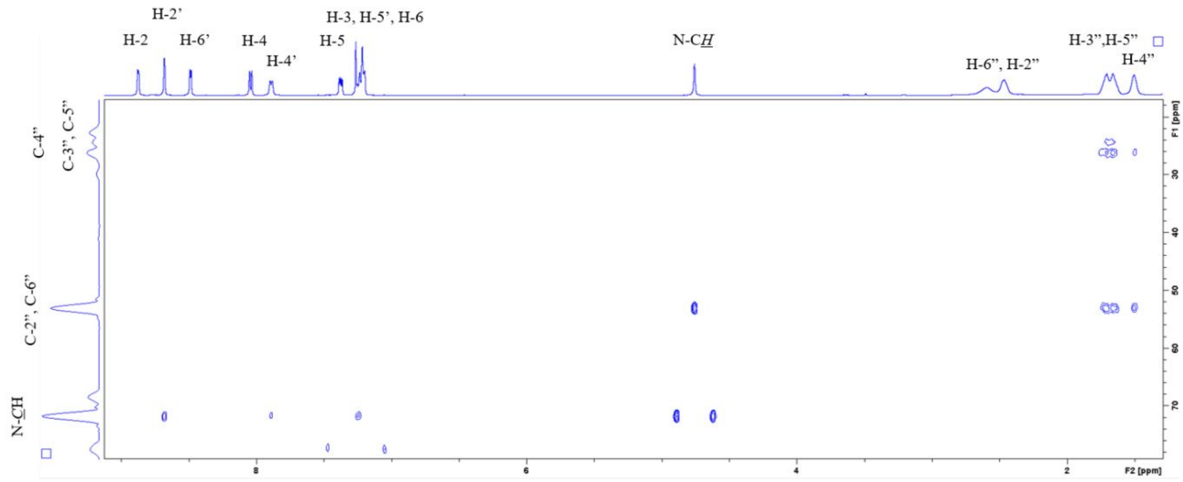

Figure S35: HMBC NMR spectrum of 7-(piperidine-1-yl)(pyridin-3-yl)methyl)quinolin-8-ol (**92**)

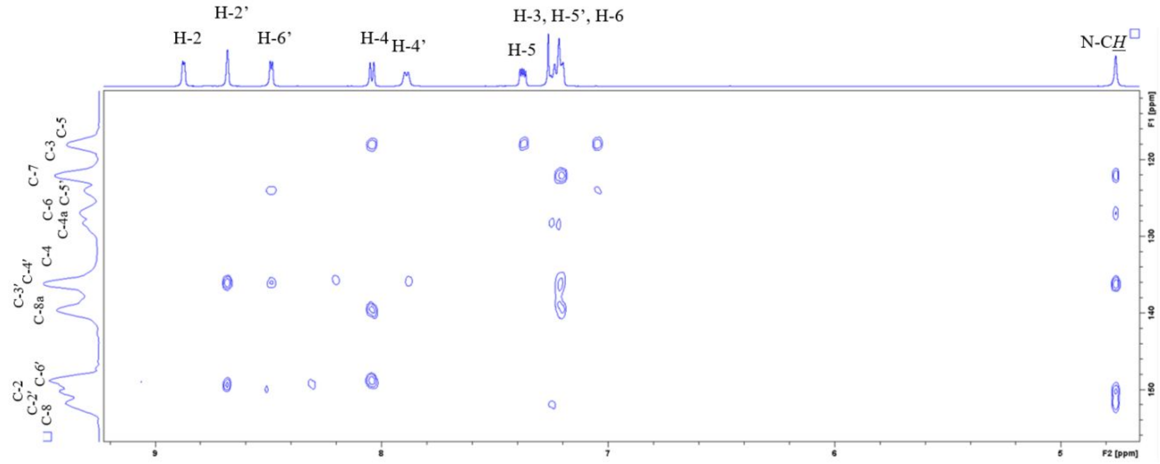

Figure S36: HMBC NMR spectrum of 7-(piperidine-1-yl)(pyridin-3-yl)methyl)quinolin-8-ol (**92**)
